# Household factors and under-five mortality in Bankass, Mali: results from a cross-sectional survey

**DOI:** 10.1186/s12889-021-10242-9

**Published:** 2021-01-29

**Authors:** David C. Boettiger, Emily Treleaven, Kassoum Kayentao, Mahamadou Guindo, Mama Coumaré, Ari D. Johnson, Caroline Whidden, Naimatou Koné, Amadou Beydi Cissé, Nancy Padian, Jenny Liu

**Affiliations:** 1 Kirby Institute, University of New South Wales, Sydney, 2252 Australia; 2 Population Studies Center, University of Michigan, Ann Arbor, USA; 3 Malaria Research and Training Center, University of Sciences, Techniques and Technologies of Bamako, Bamako, Mali; 4 Ministère de la Santé et des Affaires Sociales, Bamako, Mali; 5 Department of Medicine, University of California, San Francisco, USA; 6 Muso, Bamako, Mali; 7 School of Public Health, University of California, Berkeley, USA; 8 Institute for Health and Aging, University of California, San Francisco, USA

**Keywords:** Under-five mortality, Household, Survey, Resource-limited, Mali

## Abstract

**Background:**

Rural parts of Mali carry a disproportionate burden of the country’s high under-five mortality rate. A range of household factors are associated with poor under-five health in resource-limited settings. However, it is unknown which most influence the under-five mortality rate in rural Mali. We aimed to describe household factors associated with under-five mortality in Bankass, a remote region in central Mali.

**Methods:**

We analysed baseline household survey data from a trial being conducted in Bankass. The survey was administered to households between December 2016 and January 2017. Under-five deaths in the five years prior to baseline were documented along with detailed information on household factors and women’s birth histories. Factors associated with under-five mortality were analysed using Cox regression.

**Results:**

Our study population comprised of 17,408 under-five children from 8322 households. In the five years prior to baseline, the under-five mortality rate was 152.6 per 1000 live births (158.8 and 146.0 per 1000 live births for males and females, respectively). Living a greater distance from a primary health center was associated with a higher probability of under-five mortality for both males (adjusted hazard ratio [aHR] 1.53 for ≥10 km versus < 2 km, 95% confidence interval [CI] 1.25–1.88) and females (aHR 1.59 for ≥10 km versus < 2 km, 95% CI 1.27–1.99). Under-five male mortality was additionally associated with lower household wealth quintile (aHR 1.47 for poorest versus wealthiest, 95%CI 1.21–1.78), lower reading ability among women of reproductive age in the household (aHR 1.73 for cannot read versus can read, 95%CI 1.04–2.86), and living in a household with access to electricity (aHR 1.16 for access versus no access, 95%CI 1.00–1.34).

**Conclusions:**

U5 mortality is very high in Bankass and is associated with living a greater distance from healthcare and several other household factors that may be amenable to intervention or facilitate program targeting.

**Supplementary Information:**

The online version contains supplementary material available at 10.1186/s12889-021-10242-9.

## Background

Although global under-five (U5) mortality has declined substantially over the past 25 years, national estimates for Mali have consistently been among the highest in the world [1]. In 2018, the average estimated U5 mortality rate was 5 deaths per 1000 live births in high-income countries, among low-income countries it was 68 deaths per 1000 live births, and in Mali the estimated U5 mortality rate was 101 deaths per 1000 live births [2, 3]. Rural parts of Mali carry a disproportionate burden of the country’s high U5 mortality rate [3].

A range of household factors are known to be associated with poor U5 health in resource-limited settings. For example, long distances between the home and healthcare reduce the utilization of health services and increases child mortality [4–10], lower levels of maternal education and literacy are associated with increased probability of child mortality [11, 12], poor access to safe water and safe sanitation are leading risk factors for diarrhoea and diarrhoea-associated mortality in U5 children [13], unfinished housing is associated with a high prevalence of malaria and U5 mortality [14–17], and indoor air pollution substantially increases the risk of childhood pneumonia and mortality [18]. However, it is unknown which household factors most influence the high U5 mortality rate in rural Mali. Understanding these factors is important because the close connection individuals have with their household and limited government resources available in Mali make household-level policy interventions a better option than individual-level interventions in this setting.

The current analysis forms part of a larger study evaluating the effect of proactive case detection by community health workers on U5 mortality in the Bankass health district [19]. Bankass is situated in the Mopti region of Mali, an area that relies heavily on agriculture and that serves as an important crossroad between the country’s north, south and bordering countries [20]. It is one of the poorest regions of Mali and has one of the country’s highest burdens of U5 mortality, 130 deaths per 1000 live births in 2018 [3]. Prior research has documented improvements in early access to care and U5 mortality in peri-urban Mali after the roll-out of community and primary healthcare interventions to remove geographic and infrastructural barriers to care [21]. We, therefore, hypothesized we would find that living a greater distance from healthcare would be strongly associated with a higher rate of U5 mortality in rural Mali.

Our primary aim was to describe household factors associated with U5 mortality in Bankass. We examined these factors separately among girls and boys as there are important influences that could contribute to these factors differing by sex. Firstly, boys have a higher biological risk of mortality in early childhood [22, 23]. Secondly, in settings such as Mali, females are socially disadvantaged compared to their male counterparts leading to greater investment and resource allocation for male health [24]. This research will provide critical information for health policy makers in the region seeking to make long-term and sustainable investments into permanently reducing U5 mortality.

## Methods

### Household surveys

We analysed cross-sectionally collected baseline household survey data from a three-year cluster randomized controlled trial being conducted in 137 villages distributed across seven of the 22 health catchment areas in Bankass (Kanibonzon, Ende, Dimbal, Doundé, Soubala, Koulongon, and Lessagou). The study area has a population of approximately 100,000 people. The trial primarily aims to determine whether door-to-door proactive case detection by community health workers reduces U5 mortality compared to passive, site-based care offered under the standard Integrated Community Case Management protocol [25]. Further details on the trial protocol are available elsewhere [19].

As a part of the trial, a survey is administered to all households in the study area at baseline and every 12 months thereafter during the study period. All households were censused between December 2016 and January 2017, just before the launch of the intervention, to enumerate all permanent residents (present more than 50% of the time in the past year). The census included a household roster to collect the age, date of birth, and sex of permanent residents, as well as information about deaths in the household in the past five years. All women in the household aged 15–49 years (i.e., women of reproductive age) who provided written informed consent were then administered a baseline survey (see DEMOGRAPHICS SURVEY – Baseline in Supplementary Material). The survey was adapted from the Demographic and Health Surveys (DHS) [26], and included detailed information on household factors. Based on standard DHS modules, women were given a reading test to assess literacy and asked if they contribute to household decision-making, if their husband has more than one wife or partner, and if they felt their husband hitting or beating them was justified under certain circumstances. Geographical co-ordinates were collected using global positioning technology. We supplemented the baseline survey data with information from the year-one follow-up survey (see DEMOGRAPHICS SURVEY – Year One in Supplementary Material), administered from February to March 2018, which used the same structure as the baseline survey but added details on women’s birth histories (i.e., probes to distinguish between live and still births, clarification of multiple births, and greater precision on birth dates); this enabled us to correct any misreporting in the birth histories recorded at baseline. The survey instrument was designed in Open Data Kit, which permits real-time quality and completeness control on data collection. The final dataset was de-identified, cleaned and compiled by the data management team at Muso, a non-government organization based in Bamako, and the Malaria Research and Training Center, University of Sciences, Techniques and Technologies of Bamako.

### Inclusion criteria

Children were required to be living in a household that participated in the baseline and year-one surveys as the additional birth history data collected at year-one (see *Household Surveys above*) was required to calculate mortality rates. Children born in the five years prior to baseline were included in our analyses.

### Definitions

Household wealth was defined in quintiles using a principal components analysis of household possession of durable goods [27]. Women were considered to contribute to household decisions if they indicated participation in decision-making. Literacy was categorized based on the highest level of reading ability among women surveyed within the household. Schooling was categorized as having had any formal schooling. Polygamy was defined as a survey respondent in the household indicating that her husband/partner had more than one wife/partner. Women were considered to have tolerant views towards spousal violence if they indicated that their husband hitting or beating them was justified under any of the circumstances evaluated in the survey. We used the World Health Organization definitions of improved/unimproved water supply and sanitation [28]. Households were also asked if they treated their water to make it safer for drinking. Treatment included boiling, adding sterilizing chemicals, filtering, and solar disinfection. Roofing, wall, and flooring materials were defined as finished, rudimentary, or natural as per DHS definitions [26]. Distance to the nearest primary health center was defined as the Euclidean distance from the household village to the closest primary health center. All distance calculations were conducted in QGIS Version 3.4.6-Madeira (QGIS Development Team (2019), QGIS Geographic Information System, Open Source Geospatial Foundation Project, http://qgis.osgeo.org).

### U5 mortality rates

U5 mortality rates for the five years immediately preceding the baseline survey were calculated using full birth history data. Women reported all live births using a birth history module modelled on the DHS [26]. We used a synthetic cohort life table approach to estimate the number of deaths per 1000 live births [29]. Briefly, this method combines mortality probabilities for small age segments into the standard age segments (neonatal, infant, child, and U5). We adopted the age segments 0, 1–2, 3–5, 6–11, 12–23, 24–35, 36–47, 48–59 months. Where children were missing information about their date of birth, we used available data to narrow the range of possible values. First, we determined an unconstrained range from available information about the month and year of birth. Next, we narrowed the possible range of dates based on other available information from the child’s mother, such as age at marriage and sibling dates of birth. Then, if a range of dates still existed, we randomly assigned a date within that range. For children with missing age or date of death, we imputed age at death based on a hot deck technique, assigning data from the preceding child of the same birth order and form of reporting (day, month, and/or year). No children were missing information on the date of interview or vital status.

### Statistical analysis

The primary outcome was U5 mortality. Household-level factors analyzed in both boys and girls were ethnicity, wealth quintile, decision making contribution of women in household, highest level of reading ability among women in household, schooling among women in household, polygamy, domestic violence tolerance, water source, sanitation method, roofing material, wall material, flooring material, electricity access, primary cooking fuel, recent food shortage, livestock ownership, access to motorized transport, and distance of household from nearest healthcare center. Cox regression was used to evaluate factors associated with U5 mortality. Children were considered at risk of death from their date of birth. Follow-up was censored on the day children turned five-years-old or at the time their household completed the baseline survey, whichever came first. Children who died on their day of birth were considered to have one day of follow up so that they were not excluded from the analyses.

All regression analyses were adjusted for household clustering. Covariates were subject to univariate analysis and all were included in our multivariate analyses. Significance for hazard ratios (HRs) was defined as the lower bound of the 95% confidence interval (95%CI) being greater than 1 or the upper bound of the 95%CI being less than 1. Multicollinearity was evaluated by calculating variance inflation factors for each covariate. Children with missing covariate data were included in analyses but HRs for missing categories are not reported. Analyses were conducted with Stata 16 (Stata Corp., College Station, Texas).

## Results

Of 15,839 households censused at baseline, 268 (1.7%) were excluded because they did not have baseline and year-one survey data, 2632 (16.6%) did not house a woman of reproductive age, and 4617 (29.1%) did not have a birth in the past five years. This left a final sample of 8322 households and 17,408 children (8911 boys and 8497 girls). Most children in our sample were of Dogon ethnicity (93.2%). There was little difference between males and females in terms of household characteristics. Further details are presented in Table 1. In total, 2293 U5 deaths occurred in 1837 households (363 households reported more than one death) at a rate of 152.6 per 1000 live births (95%CI 145.2–159.2). Neonatal, infant, and child mortality rates are provided in Table 2.

**Table 1:** Household factors of children under-five in Bankass

Household factors		Children under five (***n*** = 17,408)	Males under five (***n*** = 8911)	Females under five (***n*** = 8497)
**Ethnicity**	Dogon	16,230 (93.2)	8317 (93.3)	7913 (93.1)
	Fulani	762 (4.4)	396 (4.4)	366 (4.3)
	Other	416 (2.4)	198 (2.2)	218 (2.6)
**Wealth quintile**	Wealthiest	3567 (20.5)	1865 (20.9)	1702 (20.0)
	Wealthy	3577 (20.5)	1805 (20.3)	1772 (20.9)
	Middle	3484 (20.0)	1772 (19.9)	1712 (20.1)
	Poor	3200 (18.4)	1638 (18.4)	1562 (18.4)
	Poorest	3525 (20.2)	1801 (20.2)	1724 (20.3)
	Unknown	55 (0.3)	30 (0.3)	25 (0.3)
**Decision making contribution of women in household**	Do not contribute	11,600 (66.6)	5961 (66.9)	5639 (66.4)
	Contribute	5071 (29.1)	2559 (28.7)	2512 (29.6)
	Unknown	737 (4.2)	391 (4.4)	346 (4.1)
**Highest level of reading ability among women in household**	Can read	714 (4.1)	356 (4.0)	358 (4.2)
	Can partly read	598 (3.4)	283 (3.2)	315 (3.7)
	Cannot read	15,295 (87.9)	7846 (88.0)	7449 (87.7)
	Unknown	801 (4.6)	426 (4.8)	375 (4.4)
**Schooling among women in household**	Any schooling	1917 (11.0)	934 (10.5)	983 (11.6)
	No schooling	14,755 (84.8)	7586 (85.1)	7169 (84.4)
	Unknown	736 (4.2)	391 (4.4)	345 (4.1)
**Polygamy**	Monogamous	8856 (50.9)	4518 (50.7)	4338 (51.1)
	Polygamous	7684 (44.1)	3938 (44.2)	3746 (44.1)
	Unknown	868 (5.0)	455 (5.1)	413 (4.9)
**Domestic violence**	Not tolerated	3895 (22.4)	1996 (22.4)	1899 (22.3)
	Tolerated	12,593 (72.3)	6427 (72.1)	6166 (72.6)
	Unknown	920 (5.3)	488 (5.5)	432 (5.1)
**Water source**	Improved/treated	2816 (16.2)	1416 (15.9)	1400 (16.5)
	Improved/untreated	6581 (37.8)	3407 (38.2)	3174 (37.4)
	Unimproved/treated	1491 (8.6)	739 (8.3)	752 (8.9)
	Unimproved/untreated	6379 (36.6)	3280 (36.8)	3099 (36.5)
	Unknown	141 (0.8)	69 (0.8)	72 (0.8)
**Sanitation**	Improved	8353 (48.0)	4279 (48.0)	4074 (47.9)
	Unimproved	8933 (51.3)	4568 (51.3)	4365 (51.4)
	Unknown	122 (0.7)	64 (0.7)	58 (0.7)
**Roofing material**	Natural	550 (3.2)	276 (3.1)	274 (3.2)
	Rudimentary	1509 (8.7)	756 (8.5)	753 (8.9)
	Finished	15,282 (87.8)	7839 (88.0)	7443 (87.6)
	Unknown	67 (0.4)	40 (0.4)	27 (0.3)
**Wall material**	Natural	5719 (32.9)	2888 (32.4)	2831 (33.3)
	Rudimentary	1628 (9.4)	850 (9.5)	778 (9.2)
	Finished	9757 (56.0)	5002 (56.1)	4755 (56.0)
	Unknown	304 (1.7)	171 (1.9)	133 (1.6)
**Flooring material**	Natural	16,563 (95.1)	8487 (95.2)	8076 (95.0)
	Rudimentary	231 (1.3)	120 (1.3)	111 (1.3)
	Finished	586 (3.4)	290 (3.3)	296 (3.5)
	Unknown	28 (0.2)	14 (0.2)	14 (0.2)
**Electricity**	Yes	6413 (36.8)	3292 (36.9)	3121 (36.7)
	No	10,995 (63.2)	5619 (63.1)	5376 (63.3)
**Primary cooking fuel**	Wood	14,926 (85.7)	7632 (85.6)	7294 (85.8)
	Straw	2200 (12.6)	1136 (12.7)	1064 (12.5)
	Animal dung	186 (1.1)	92 (1.0)	94 (1.1)
	Other	96 (0.6)	51 (0.6)	45 (0.5)
**Food shortage in past 30 days**	Yes	14,835 (85.2)	7617 (85.5)	7218 (84.9)
	No	2573 (14.8)	1294 (14.5)	1279 (15.1)
**Livestock**	Any	15,705 (90.2)	8028 (90.1)	7677 (90.3)
	Cows/bulls	11,385 (65.4)	5829 (65.4)	5556 (65.4)
	Horses/donkeys/mules	11,635 (66.8)	5919 (66.4)	5716 (67.3)
	Goats	8128 (46.7)	4116 (46.2)	4012 (47.2)
	Sheep	12,629 (72.5)	6401 (71.8)	6228 (73.3)
	Chickens	12,140 (69.7)	6207 (69.7)	5933 (69.8)
**Motorized transport**	Any	9253 (53.2)	4722 (53.0)	4531 (53.3)
	Motorbike/scooter	9218 (53.0)	4710 (52.9)	4508 (53.1)
	Car/truck	117 (0.7)	55 (0.6)	62 (0.7)
**Nearest healthcare center, kilometers**	< 2	3084 (17.7)	1590 (17.8)	1494 (17.6)
	2–4.99	4442 (25.5)	2265 (25.4)	2177 (25.6)
	5–6.99	4126 (23.7)	2123 (23.8)	2003 (23.6)
	7–9.99	3516 (20.2)	1804 (20.2)	1712 (20.1)
	≥10	2240 (12.9)	1129 (12.7)	1111 (13.1)

All values are *n* (%*N*)

**Table 2:** Early childhood mortality rates by sex in the five years prior to the baseline survey

	Neonatal mortality	Infant mortality	Child mortality	Under-five mortality
**Males**	45.1 (41.0–49.6)	86.7 (80.9–92.9)	79.0 (71.2–87.5)	158.8 (149.8–168.3)
**Females**	39.5 (35.5–43.9)	79.2 (73.5–85.3)	72.5 (64.9–80.9)	146.0 (137.0–155.4)
**Total**	42.4 (39.5–45.5)	83.1 (78.9–87.4)	75.8 (70.3–81.7)	152.6 (145.2–159.2)

All values are in units of deaths per 1000 live births (95% confidence interval)

A total of 1213 U5 male deaths occurred at a rate of 158.8 per 1000 live births (95%CI 149.8–168.3). Table 3 shows that the factors most strongly associated with the death of an U5 male were greater distance from a primary health center (adjusted HR [aHR] 1.53 for ≥10 km versus < 2 km, 95%CI 1.25–1.88), lower household wealth quintile (aHR 1.47 for poorest versus wealthiest, 95%CI 1.21–1.78), lower reading ability among women of reproductive age in the household (aHR 1.73 for cannot read versus can read, 95%CI 1.04–2.86), and living in a household with access to electricity (aHR 1.16 for access versus no access, 95%CI 1.00–1.34).

**Table 3:** Household factors associated with male under-five mortality (N = 8911)

Household characteristic		Bivariate hazard ratio (95%CI)	Multivariate hazard ratio (95%CI)
**Ethnicity**	Dogon	1.00	1.00
	Fulani	1.01 (0.75–1.35)	0.82 (0.59–1.15)
	Other	1.02 (0.68–1.52)	0.94 (0.63–1.41)
**Wealth quintile**	Wealthiest	1.00	1.00
	Wealthy	1.23 (1.02–1.48)	**1.26 (1.04–1.53)**
	Middle	1.17 (0.97–1.43)	1.22 (0.99–1.51)
	Poor	1.16 (0.95–1.40)	1.24 (0.99–1.56)
	Poorest	1.42 (1.18–1.70)	**1.47 (1.21–1.78)**
**Decision making contribution of women in household**	Contribute	1.00	1.00
	Do not contribute	1.02 (0.89–1.16)	1.04 (0.91–1.19)
**Highest level of reading ability among women in household**	Can read	1.00	1.00
	Can partly read	1.59 (0.96–2.65)	1.60 (0.95–2.70)
	Cannot read	1.48 (1.02–2.14)	**1.73 (1.04–2.86)**
**Schooling among women in household**	Any schooling	1.00	1.00
	No schooling	1.02 (0.84–1.25)	0.84 (0.62–1.13)
**Polygamy**	Monogamous	1.00	1.00
	Polygamous	1.10 (0.98–1.24)	1.10 (0.98–1.25)
**Domestic violence**	Not tolerated	1.00	1.00
	Tolerated	1.00 (0.86–1.15)	1.01 (0.87–1.17)
**Water source**	Improved/treated	1.00	1.00
	Improved/untreated	0.88 (0.73–1.05)	0.85 (0.71–1.01)
	Unimproved/treated	0.91 (0.72–1.16)	0.87 (0.68–1.11)
	Unimproved/untreated	0.95 (0.79–1.13)	0.88 (0.73–1.06)
**Sanitation**	Improved	1.00	1.00
	Unimproved	1.14 (1.02–1.29)	1.09 (0.96–1.23)
**Roofing material**	Finished	1.00	1.00
	Rudimentary	1.15 (0.94–1.40)	1.13 (0.92–1.39)
	Natural	1.19 (0.87–1.63)	1.11 (0.81–1.51)
**Wall material**	Finished	1.00	1.00
	Rudimentary	0.99 (0.81–1.22)	0.96 (0.78–1.18)
	Natural	1.02 (0.89–1.16)	1.05 (0.92–1.20)
**Flooring material**	Finished	1.00	1.00
	Rudimentary	1.05 (0.54–2.04)	1.05 (0.54–2.06)
	Natural	1.16 (0.81–1.66)	1.10 (0.76–1.59)
**Electricity**	No	1.00	1.00
	Yes	1.09 (0.96–1.23)	**1.16 (1.00–1.34)**
**Primary cooking fuel**	Wood	1.00	1.00
	Straw	1.08 (0.91–1.28)	1.02 (0.85–1.22)
	Animal dung	1.49 (0.93–2.38)	1.50 (0.93–2.42)
	Other	1.27 (0.63–2.53)	1.32 (0.67–2.61)
**Food shortage in past 30 days**	No	1.00	1.00
	Yes	1.11 (0.95–1.31)	1.05 (0.89–1.24)
**Livestock**	No	1.00	1.00
	Yes	1.11 (0.90–1.37)	1.09 (0.86–1.37)
**Motorized transport**	No	1.00	1.00
	Yes	0.96 (0.86–1.08)	0.95 (0.84–1.08)
**Nearest healthcare center, kilometers**	< 2	1.00	1.00
	2–4.99	0.87 (0.71–1.06)	0.84 (0.68–1.02)
	5–6.99	0.85 (0.70–1.04)	0.82 (0.67–1.01)
	7–9.99	1.27 (1.06–1.54)	**1.23 (1.01–1.50)**
	≥10	1.59 (1.31–1.93)	**1.53 (1.25–1.88)**

Significant multivariate hazard ratios are bolded. Significance was defined as the lower bound of the 95%CI being greater than 1 or the upper bound of the 95%CI being less than 1. Children with missing covariate data were included in all analyses but hazard ratios for missing categories are not reported. *CI* confidence interval

There were 1080 U5 female deaths which occurred at a rate of 146.0 per 1000 live births (95%CI 137.0–155.4). Table 4 shows that the household factor most strongly associated with the death of an U5 female was greater distance from a primary health center (aHR 1.59 for ≥10 km versus < 2 km, 95%CI 1.27–1.99).

**Table 4:** Household factors associated with female under-five mortality (*N* = 8497)

Household characteristic		Bivariate hazard ratio (95%CI)	Multivariate hazard ratio (95%CI)
**Ethnicity**	Dogon	1.00	1.00
	Fulani	1.06 (0.81–1.41)	1.01 (0.73–1.41)
	Other	1.18 (0.83–1.68)	1.12 (0.78–1.60)
**Wealth quintile**	Wealthiest	1.00	1.00
	Wealthy	0.98 (0.81–1.19)	0.94 (0.77–1.15)
	Middle	1.09 (0.90–1.33)	1.12 (0.92–1.38)
	Poor	1.00 (0.82–1.22)	1.09 (0.87–1.37)
	Poorest	1.04 (0.86–1.26)	1.07 (0.88–1.31)
**Decision making contribution of women in household**	Contribute	1.00	1.00
	Do not contribute	0.91 (0.79–1.03)	0.96 (0.83–1.10)
**Highest level of reading ability among women in household**	Can read	1.00	1.00
	Can partly read	0.94 (0.60–1.48)	0.93 (0.59–1.46)
	Cannot read	1.05 (0.76–1.44)	0.78 (0.50–1.22)
**Schooling among women in household**	Any schooling	1.00	1.00
	No schooling	1.17 (0.95–1.43)	1.34 (0.97–1.85)
**Polygamy**	Monogamous	1.00	1.00
	Polygamous	1.09 (0.96–1.23)	1.06 (0.93–1.20)
**Domestic violence**	Not tolerated	1.00	1.00
	Tolerated	1.14 (0.98–1.32)	1.13 (0.97–1.31)
**Water source**	Improved/treated	1.00	1.00
	Improved/untreated	1.21 (1.00–1.46)	1.18 (0.97–1.43)
	Unimproved/treated	1.31 (1.02–1.68)	1.20 (0.93–1.55)
	Unimproved/untreated	1.11 (0.91–1.34)	1.05 (0.85–1.28)
**Sanitation**	Improved	1.00	1.00
	Unimproved	1.07 (0.95–1.21)	1.05 (0.93–1.20)
**Roofing material**	Finished	1.00	1.00
	Rudimentary	1.06 (0.86–1.31)	1.09 (0.88–1.35)
	Natural	1.05 (0.74–1.48)	1.03 (0.72–1.47)
**Wall material**	Finished	1.00	1.00
	Rudimentary	0.90 (0.72–1.13)	0.94 (0.75–1.17)
	Natural	0.97 (0.85–1.11)	0.98 (0.86–1.13)
**Flooring material**	Finished	1.00	1.00
	Rudimentary	1.05 (0.52–2.14)	1.07 (0.51–2.23)
	Natural	1.15 (0.81–1.63)	1.10 (0.77–1.56)
**Electricity**	No	1.00	1.00
	Yes	1.10 (0.97–1.25)	1.12 (0.97–1.30)
**Primary cooking fuel**	Wood	1.00	1.00
	Straw	1.17 (0.98–1.40)	1.12 (0.93–1.35)
	Animal dung	1.39 (0.84–2.32)	1.33 (0.77–2.31)
	Other	1.14 (0.42–3.12)	1.22 (0.44–3.39)
**Food shortage in past 30 days**	No	1.00	1.00
	Yes	1.18 (1.01–1.38)	1.10 (0.94–1.30)
**Livestock**	No	1.00	1.00
	Yes	0.96 (0.78–1.18)	0.91 (0.73–1.13)
**Motorized transport**	No	1.00	1.00
	Yes	1.06 (0.94–1.20)	1.07 (0.94–1.23)
**Nearest healthcare center, kilometers**	< 2	1.00	1.00
	2–4.99	1.06 (0.86–1.30)	1.04 (0.84–1.29)
	5–6.99	1.00 (0.81–1.24)	0.98 (0.79–1.22)
	7–9.99	1.40 (1.14–1.72)	**1.37 (1.11–1.70)**
	≥10	1.67 (1.34–2.08)	**1.59 (1.27–1.99)**

Significant multivariate hazard ratios are bolded. Significance was defined as the lower bound of the 95%CI being greater than 1 or the upper bound of the 95%CI being less than 1. Children with missing covariate data were included in all analyses but hazard ratios for missing categories are not reported. *CI* confidence interval

All covariates in our male and female analyses had a variance inflation factor < 3 indicating multicollinearity was not an issue in our final models.

## Discussion

U5 mortality is very high in Bankass. In our male and female analyses, greater distance from a primary health center was consistently associated with a high rate of U5 mortality. In our male analysis, poorer wealth quintile, poorer reading ability among women of reproductive age in the household, and having access to electricity were also significantly associated with U5 mortality.

We estimated an U5 mortality rate of 152.6 per 1000 live births for the five years prior to our baseline survey. Although the U5 mortality rate in Mopti is high compared with the rest of Mali (130 per 1000 live births versus 101 per 1000 live births in 2018 [3]), our findings indicate rates are particularly high in Bankass. Consistent with regional and national data [3], we also found that U5 mortality rates were higher among males than females.

Large distances between home and healthcare have previously been found to reduce the utilization of health services [4–6] and have been linked to increased child mortality in other parts of sub-Saharan Africa [7–10]. Our findings support this earlier work and reinforce the primary motivation for our clinical trial evaluating the benefit of community health workers conducting proactive case detection and management in rural Mali, as compared to passive site-based care. Elsewhere, community case management has proven to increase care-seeking outside the home [30], and reduce U5 mortality [21, 31–33].

A large volume of literature has described the high rate of U5 mortality among poor households compared with wealthy households in low- and middle-income settings [34]. The findings in our male analysis reiterate this body of work. However, in our female analysis, wealth quintile was not associated with mortality. This is consistent with households in resource-limited countries tending to invest more into male child health [24], as greater investment is likely to result in a clearer distinction between levels of wealth. It is also possible that the high prevalence of poverty in Bankass (over 75% of households in our sample fell within the nation’s poorest wealth quintile when wealth index was standardized to the 2018 Mali DHS [3]) contributed to a general lack of distinction between levels of wealth.

The significant associations we found between U5 mortality and poor maternal reading ability, and U5 mortality and access to electricity, were only seen for males. Why girls appear to be less impacted warrants further investigation. Higher levels of maternal education and literacy have been shown to reduce the probability of infant and child mortality in sub-Saharan Africa [11, 12]. Educated women have stronger cognitive, comprehension, and communication skills, which may support healthier behaviors that ultimately lead to lower child mortality [12]. Access to electricity in the home is generally considered a marker of improved living standards and has been associated with lower child mortality in low- and middle-income countries [35]. In Bankass, there is no electricity grid. Hence, households must rely on fuelled generators, solar power, and batteries. Some households may use funds to maintain access to electricity while reducing their budget for healthcare. However, this association requires further exploration.

The main limitation of this study is that many responses were subjective or subject to recall bias. Birth histories are particularly subject to recall bias as mothers may be more likely to omit information about births that occurred in the distant past or births of children who died [36]. Nevertheless, birth histories are one of the most reliable ways to obtain birth data in settings where vital records are lacking (as they are in Mali) [37]. Given the remoteness of Bankass, it is also likely there was some imprecision in the geographical coordinates documented. Further, Euclidian distance is an imperfect indicator of geographic barriers to care as it does not consider topographic characteristics that could affect the true distance travelled. Nevertheless, Euclidian distance is well correlated with true distance travelled [38], and the association we found between greater distance from a primary health provider and a high probability of U5 mortality is consistent with earlier work [7–10]. While our regression analyses assessed the association between household factors and U5 mortality, other factors (e.g., individual-level factors such as genetic susceptibility, environmental level factors such as exposure to pollution) may contribute to the high U5 mortality rate in Bankass. Our study concentrated on household factors as these are likely to be amenable to successful policy intervention. Finally, we were missing data for some of our model covariates. Our modelling adopted the well-accepted approach of including a missing category for these covariates. However, it should be acknowledged that data may not have been missing at random and could therefore have introduced bias in our estimates. Importantly, the volume of missing data was small.

## Conclusions

U5 mortality is very high in Bankass and is associated with living a greater distance from healthcare and several other household factors that may be amenable to intervention or facilitate program targeting. Health policy-makers should consider these findings when developing future interventions aimed at curbing U5 mortality in the region and other parts of rural Mali.

## Supplementary Information

**Additional file 1:.** DEMOGRAPHICS SURVEYS – Baseline and Year One.

## Data Availability

Muso Inc. (https://www.musohealth.org/) own the data used for this study and granted access to the authors. These data are not publicly available to maintain participant privacy but may be available from the corresponding author upon reasonable request.

## Abbreviations

aHR: Adjusted hazard ratio
CI: Confidence interval
DHS: Demographic and Health Surveys
HR: Hazard ratio
U5: Under-five
