# Supplementary material for: Household factors and under-five mortality in Bankass, Mali: results from a cross-sectional survey
Source: BMC Public Health. 2021 Jan 29;21:244. doi: 10.1186/s12889-021-10242-9 (PMC7845123; doi:10.1186/s12889-021-10242-9)
Supplement: Supplementary file 1 — Additional file 1:. DEMOGRAPHICS SURVEYS – Baseline and Year One. [file 12889_2021_10242_MOESM1_ESM.pdf]

DEMOGRAPHICS SURVEY - Baseline

Bankass

HOUSEHOLD CENSUS:

(1) LIST OF MEMBERS

H101. Date

- ENTER DD/MM/YYYY, with or without slashes

DATE

H102. Interviewer ID

INTERVIEWER ID

H103. Language of Interview

1. Bamanankan	2. Tingu	3. Tomoso	4. Peulh	5. French	6. Other-specify (H103S. Specify (String 100))

H104. Area (NAME)

ANAME (H104S. Précisez (String 100))

H105. Village (NAME)

VNAME (H105S. Précisez (String 100))

H106. Hamlet (NAME or N/A)

HNAME (H106S. Précisez (String 100))	N/A

H107. Concession No.

- ENTER the concession number (X/X/X/X), 4 digits

CONCESSIONNO

H108. Household No.

- ENTER the household number (X/X), 2 digits

HOUSEHOLDNO

Household Members Loop (Max = 50)

Identify all household members. A member of the household is someone who usually lives in the household, who shared the meals in the household for at least one year, and who has no other residence. Someone who has recently moved and does not plan to return within a year is not considered a household member.

H109. RECORD Member ID

- Head/Chief = 000
- Woman = 100
- BEGIN with the head of the household
- ENTER the member's identification number (X/X/X)

MEMBERID

H110. First & Family Name

NAME (H110S. Specify (String 100))

H111. Gender

1. Male	2. Female

H112. Age

How old is [NAME]?

Years-Specify (0-100)	Months-Specify (0-12)	Days-Specify (0-31)

H113. What is [NAME]'s date of birth?

- ENTER the date (DD/MM/YYYY), with or without slashes.

DOB

H113CHKPT: WHETHER RECORDED DOB MATCHES AGE

1. RECORDED DATE OF BIRTH DOES NOT MATCH AGE (H112 – H113 ≠ 0)	2. RECORDED DATE OF BIRTH DOES MATCH AGE (H112 – H113 = 0)	→ GO TO H115
↓		

* If the recorded date of birth does not match the age, you must correct the inconsistency. To do this, ask for clarification on age, date of birth, or both. It is important to understand that one or the other of the two pieces of information may be incorrect. Do not assume, for example, that it is always the date of birth that was given correctly and that it is the age that is incorrect. The date or age, or date and age may not be correct.
If the difference between age and DDN is not equal to 0, probe:

H114. I have recorded that (NAME) is X months / years, and that he / she was born in (MONTH / YEAR). I want to make sure that I recorded the exact age of (NAME).

(NAME) is how many months / years old?

- ENTER years

  [0-99]

- ENTER months

  [0-12]

- ENTER days

  [0-31]

(NAME) was born in what month / year?

- ENTER year

  [1900-2100]

- ENTER month

  [00-12]

- ENTER day

  [00-31]

H115. Relationship to Head of Household

1. Head of household	2. Spouse	3. Daughter / son	4. Father / mother	5. Brother / sister	6. Son-in-law / daughter-in-law
7. Grandchild	8. Grandparent	9. Cousin	10. Nephew / niece	11. Uncle / aunt	12. Visitor	13. Foster child / adopted
14. Other-specify (H115S. Specify (String 100))					

H116. Status of Household Member

- Present = Physically present in the village during the survey period in the village
- Absent/travel = Absent from the village or traveling during the survey period in the village

1. Present	2. Absent / Travel

End of Household Members Loop

HOUSEHOLD CENSUS:

(2) MORTALITY

H201. In the last five years, that is, since the last cereal harvest before ATT's coup d'état until now, has anyone in your household died?

1. Yes	2. No → GO TO 104	8. Do not Know → GO TO 104	9. No Response → GO TO 104

↓

Mortality Loop (Max = 20)

H202. Beginning with the most recent death, please, tell me:

The name of the person?

- ENTER FIRST NAME & FAMILY NAME

NAME (String 100)
FAMILYNAME (String 100)

H203. Relationship to head of household?

1. Head of household	2. Spouse	3. Daughter / son	4. Father / mother	5. Brother / sister	6. Son-in-law / daughter-in-law
7. Grandchild	8. Grandparent	9. Cousin	10. Nephew / niece	11. Uncle / aunt	12. Visitor
13. Foster child / adopted	14. Other-specify (H203S. Specify (String 100))				

H204. What is the date of death of [NAME]?

- ENTER BOTH THE MONTH AND YEAR OF DEATH. Both fields are mandatory
- ENTER the month of death (mark the month in 2 digits, 1 to 12)
- If unknown month, ENTER 88
- If no response, ENTER 99

01-12, 88, 99

- ENTER the year of death (mark the year in 4 digits, like 1960)
- If unknown year, ENTER 8888.
- If no response, ENTER 9999.

1900-2100, 8888, 9999

H205. What is the date of birth of [NAME]?

- ENTER day
- If unknown day, ENTER 88.
- If no response, ENTER 99.

01-12, 88, 99

- ENTER month
- If unknown month, ENTER 88.
- If no response, ENTER 99.

01-31, 88, 99

- ENTER year
- If unknown month, ENTER 8888.
- If no response, ENTER 9999.

1900-2100, 8888, 9999

H206. How old was (NAME) when he died?

- ALL FIELDS ARE MANDATORY
- ENTER years of age
- MARK 00 years if the child is under one year
- If unknown number of years, ENTER 88
- If no response, ENTER 99

00-99

- ENTER months of age
- MARK 00 months if the child is less than one month old.
- If unknown number of months, ENTER 88
- If no response, ENTER 99

00-99

- ENTER days of age
- If unknown number of days, ENTER 88.
- If no response for age, ENTER 99.

00-99

H207. CHKPT: WHETHER RECORDED DOB MATCHES AGE

1. RECORDED DATE OF DEATH DOES NOT MATCH AGE (H205– H206 ≠ 0)	2. RECORDED DATE OF DEATH DOES MATCH AGE (H205 – H206= 0)	→ GO TO H208
↓		

If the difference between age and DDN is not equal to 0, probe:

H207B. I recorded that (NAME) was X months / years, and that he / she died in (MONTH / YEAR). I want to make sure that I recorded the exact age of (NAME) at the time of death. How old was (NAME) when he died?

- ENTER years
- If unknown years, ENTER 88
- If no response, enter 99

0-99

- ENTER months
- If unknown months, ENTER 88
- If no response, ENTER 99

0-99

- ENTER days
- If unknown days, ENTER 88
- If no response, ENTER 99

0-99

H207C. I want to make sure that I have entered the date of death of (NAME). What is the date of death of (NAME)?

- ENTER month (mark the month in 2 digits, 1 to 12)
- If unknown month, ENTER 88
- If no response, ENTER 99

| 01-12, 88, 99 |

- ENTER year (mark the year in 4 digits, like 1960)
- If unknown year, ENTER 8888
- If no response, ENTER 9999

| 1900-2100, 8888, 9999 |

H207D. I want to make sure that I have entered the date of birth of (NAME). What is the date of birth of (NAME)?

- ENTER day
- If unknown day, ENTER 88
- If no response, ENTER 99

| 01-31, 88, 99 |

- ENTER month
- If unknown month, ENTER 88
- If no response, ENTER 99

| 01-12, 88, 99 |

- ENTER year
- If unknown year, ENTER 8888
- If no response, ENTER 9999

| 1900-2100, 8888, 9999 |

H208. If child, is the child's biological mother currently living in the household?

| 1. Yes | 2. No → GO TO H210 | 8. Do not Know → GO TO H210 | 9. No Response → GO TO H210 |

↓

H209. Mark the last three digits of the mother's ID.

| 000-999 |

H210. Is the biological father of the child currently living in the household?

| 1. Yes | 2. No | 8. Do not Know | 9. No Response |

→ GO TO H212 → GO TO H212 → GO TO H212

↓

H211. Mark the father's ID.

000-999

H212. Was (NAME) male or female?

1. Male → GO TO H216 | 2. Female ↓

H213. Was she pregnant when she died?

1. Yes | 2. No | 8. Do not Know | 9. No Response

H214. Did she die during childbirth?

1. Yes → GO TO H216 | 2. No ↓ | 8. Do not Know ↓ | 9. No Response ↓

H215. Did she die within two months of giving birth or ending the pregnancy (abortion)?

1. Yes | 2. No | 8. Do not Know | 9. No Response

H216. In the last five years, that is, from the last cereal harvest before the ATT coup d'éta to date, did anyone else who lived in your household die?

1. Yes → GO TO H202 | 2. No ↓ | 8. Do not Know ↓ | 9. No Response ↓

<u>End of Mortality Loop (Max = 20)</u>

104. How many people does your household currently have?

- This is the household and not the concession. Count those who usually live in your household
- If the number of people in the household is unknown, ENTER 88.
- If there is no response, ENTER 99.

0-99

HOUSEHOLD CENSUS:
(3) HOUSEHOLD CHARACTERISTICS

116A. What is the main source of drinking water for members of your household?

- If piped water, SPECIFY:

  11. Piped into dwelling | 12. Piped into yard/plot | 13. Piped to neighbor | 14. Public tap / standpipe

  21. Tube well or borehole

- If dug well, SPECIFY:

  31. Protected well | 32. Unprotected well

- If water from spring, SPECIFY:

  41. Protected spring | 42. Unprotected spring

- If other, SPECIFY:
- NOTE: Surface water could be river, dam, lake, pond, stream, canal, irrigation channel

  51. Rainwater | 61. Tanker truck | 71. Cart with small tank | 81. Surface water | 91. Bottled water

  96. Other-specify (116AS. Specify (String 100)) | 88. Do not know | 99. No response

116B. Do you do anything to the water to make it safer to drink?

1. Yes | 2. No → GO TO 117A | 8. Do not Know → GO TO 117A | 9. No Response → GO TO 117A

↓

116C. What do you usually do to make the water safer to drink?

- ENTER all that apply
- PROBE: Anything else?

  1. Boil | 2. Add bleach/chlorine | 3. Strain through a cloth | 4. Use water filter (ceramic/sand/composite/etc)

  5. Solar disinfection | 6. Let it stand and settle | 7. Other-specify (116CS. Specify (String 100)) | 88. Do not Know

  99. No Response

116D. How often do you treat water to drink?

1. Always | 2. Often | 3. Sometimes | 4. Rarely | 88. Do not Know | 99. No Response

117A. What kind of toilet facility do members of your household usually use?

- If flush or pour flush toilet, SPECIFY:

11. Flush to piped sewer system	12. Flush to septic tank	13. Flush to pit latrine	14. Flush to somewhere else
15. Flush, don't know where			

- If pit latrine, SPECIFY:

21. Ventilated improved pit latrine	22. Pit latrine with slab	23. Pit latrine without slab/open pit

- If other, SPECIFY:

31. Composting toilet	41. Bucket toilet	51. Hanging toilet	61. No facility/bush/field
96. Other-specify (117AS. Specify (String 100))	88. Do not Know	99. No response	

117B. Where is this toilet facility located?

1. In own dwelling	2. In own yard/plot	3. Elsewhere

117C. Do you share this toilet facility with other households?

1. Yes	2. No

118. Does your household have:

- Electricity?

1. Yes	2. No

- A radio?

1. Yes	2. No

- A television ?

1. Yes	2. No

- A mobile phone ?

1. Yes	2. No

- A non-mobile phone?

1. Yes	2. No

- A refrigerator ?

1. Yes	2. No

- An antenna TV5?

1. Yes	2. No

- Subscription to Cable?

1. Yes	2. No

- A washing machine?

1. Yes	2. No

- A stove or a gas or electric cooker?

1. Yes	2. No

- An improved home?

1. Yes	2. No

- A video / CD / DVD player?

1. Yes	2. No

- An air conditioner?

1. Yes	2. No

- A computer?

1. Yes	2. No

- Internet at home?

1. Yes	2. No

119. What type of fuel does your household mainly use for cooking?

01. Electricity	02. LPG	03. Natural Gas	04. Biogas	05. Kerosene	06. Coal, lignite	07. Charcoal	08. Wood

09. Straw / shrubs / grass | 10. Agricultural crop | 11. Animal Dung | 95. No food cooked in household

96. Other-specify (119S. Specify (String 100)) | 88. Do not Know | 99. No Response

120. OBSEVE MAIN MATERIAL OF THE FLOOR OF THE DWELLING. RECORD OBSERVATION.

- If natural floor, SPECIFY:

  11. Earth/Sand | 12. Dung

- If rudimentary floor, SPECIFY:

  21. Wooden planks | 22. Palms / bamboo

- If finished floor, SPECIFY:

  31. Parquet or polished wood | 32. Vinyl or asphalt strips | 33. Ceramic tiles | 34. Cement | 35. Carpet

  96. Other-specify (120S. Specify (String 100))

121. Does any member of this household own:

- A watch ?

  1. Yes | 2. No

- A plow?

  1. Yes | 2. No

- A bicycle ?

  1. Yes | 2. No

- A motorcycle or a scooter?

  1. Yes | 2. No

- An animal-drawn cart?

  1. Yes | 2. No

- A canoe / or fishing nets?

  1. Yes | 2. No

- A tractor ?

  1. Yes | 2. No

- A car or truck?

1. Yes	2. No

- A boat with a motor ?

1. Yes	2. No

122. How many of the following animals does this household own?

- NONE = 00
- 95 OR MORE = 95
- DO NOT KNOW = 88
- NO RESPONSE = 99
- Livestock?

00-99

- Dairy cows or bulls?

00-99

- Horses, donkeys or mules?

00-99

- Goats?

00-99

- Sheep?

00-99

- Chickens?

00-99

123. In this household, how many rooms / places are used for sleeping?

- ENTER AND SPECIFY ALL THAT APPLY:

Rooms (ENTER 00-99)	Places (ENTER 00-99)

124. In the last 30 days, was there ever no food to eat of any kind in your house because of a lack of resources to get food?

1. Yes ↓ | 2. No → GO TO 126 | 8. Do not Know → GO TO 126 | 9. No Response → GO TO 126

125. How often did this happen in the last 30 days?

1. Rarely (1-2 times) | 2. A few times (3-10 times) | 3. Often (more than 10 times)

126. In the past 30 days, did you or any household member go to sleep at night hungry because there was not enough food?

1. Yes ↓ | 2. No → GO TO 128 | 8. Do not Know → GO TO 128

127. How often did this happen in the last 30 days?

1. Rarely (1-2 times) | 2. A few times (3-10 times) | 3. Often (more than 10 times)

128. In the past 30 days, did you or any household member go a whole day and night without eating anything because there was not enough food?

1. Yes ↓ | 2. No → GO TO 130 | 8. Do not Know → GO TO 130

129. How often did this happen in the last 30 days?

1. Rarely (1-2 times) | 2. A few times (3-10 times) | 3. Often (more than 10 times)

WOMEN'S SURVEY SCREENING

IDENTIFICATION OF THE WOMAN INTERVIEWED

C1. ENTER the date

- ENTER DD/MM/YYYY, with or without slashes

DATE

C2. ENTER name and surname of the interviewer:

INTNAME (C2S. Specify (String 100))

C3. Investigator number:

- ENTER investigator number (\X\X\)

INVESTIGATOR NUMBER

C4. Name of the village

VNAME (C4S. Specify (String 100))

C5. Name and surname of the head of the family

CHEFNAME (C5S. Specify (String 100))

C6. Telephone number of the head of the family

- ENTER the telephone number of the head of the family (XXXX/XXXX), with or without slash

CHEFPHONENO

C7. Name and surname of the woman

NAME (C7S. Specify (String 100))

C8. Age (in years) of woman

00-99

C9. Name and surname of the woman's husband

HUSNAME (C9S. Specify (String 100))

C10. Telephone number of woman's husband

- ENTER the telephone number of the woman's husband XXXX/XXXX, with or without slash

HUSPHONENO

INFORMATION ON THE SCREENING OF THE WOMAN INTERVIEWED

E1. Is the woman aged 15-49 years?

1. Yes
2. No

E2. Permanent resident?

- NB: A permanent resident is someone who usually lives in the household, who shared meals in the household for at least one year, and who has no other residence. Someone who has recently moved and does not plan to return within the year is not considered a permanent resident

1. Yes
2. No

E3. Is the woman expected to remain in the study area during the next three years?

1. Yes
2. No

E4. Is the woman willing to participate and complete the study program?

1. Yes
2. No

E5CKPT. ELIG CHECKPOINT: WHETHER PARTICIPANT IS ELIGIBLE OR NOT

- If No to one of questions 1, 2, 3 and 4 the woman is not eligible for study. So do not continue the interview
- If yes to all questions 1, 2, 3 and 4 then the woman is eligible

1. Yes, Eligible ↓
2. Not Eligible →GO TO E8

E6. Is the woman eligible?

1. Yes
2. No

E7. If eligible, is she included in the study?

1. Yes	2. No

→GO TO E9

E8. If not included, give reasons:

a. Declined consent

1. Yes	2. No

b. Need to consult family

1. Yes	2. No

c. Not convinced

1. Yes	2. No

d. Family refused consent

1. Yes	2. No

→END OF INTERVIEW

E9. Randomized village

1. Pro-CCM	2. I-CCM

Pro-CCM Randomized Controlled Trial Survey Tool
V10112016

Section 1: Demographic Details

101. Have you ever attended school (madrasah or French)?

1. Yes ↓	2. No → GO TO 103	8. Do not Know → GO TO 103	9. No response → GO TO 103

102. What is the highest level of school you have completed:
- Primary 1 (1 cycle), primary 2 (2nd cycle), secondary (high school, technical) or higher?

1. Primary 1 (1st cycle)	2. Primary 2 (2nd cycle)	3. Secondary (high school / technical)	4. More than secondary
8. Do not Know	9. No Response		

103. Now I would like you to read this sentence to me.
- SHOW CARD TO RESPONDENT.
- IF THE RESPONDENT CANNOT READ WHOLE SENTENCE, PROBE: Can you read any part of the sentence to me?
- If no card with required language, SPECIFY which language.

1. Cannot read at all	2. Able to read only part of the sentence	3. Able to read whole sentence
4. No card with required language-specify (103S. Specify (String 100))	5. Blind / visually impaired	

105. What religion do you practice?

1. Muslim	2. Catholic	3. Methodist	4. Evangelical	5. Other Christian religion	6. Animist
7. Other religions-specify (105S. Specify (String 100))	8. Without Religion	88. Do not Know	99. No Response		

106. What is your ethnicity?

1. Dogon	2. Peulh	3. Bambara	4. Mossi	5. Malinke	6. Sarakole / soninke / marka	7. Sonraï	8. Tamachek / Bella
9. Sénoufo / Minianka	10. Bobo	11. Boso	12. Other Malian ethnic-specify (106S. Specify (String 100))	13. CEDEAO country			
14. Other African countries-specify (106S. Specify (String 100))	15. Other non-African countries-specify (106S. Specify (String 100))						
88. Do not know	99. No response						

107. Are you currently married or living together with a man as if married?

1. Yes, currently married → GO TO 110 | 2. Yes, living with a man →GO TO 110

3. No, not in union ↓ | 8. Do not Know ↓ | 9. No Response ↓

108. Have you ever been married or lived together with a man as if married?

1. Yes, formerly married ↓ | 2. Yes, lived with a man ↓ | 3. No →GO TO 130

109. What is your marital status now: are you widowed, divorced or separated?

1. Widowed →GO TO 114 | 2. Divorced →GO TO 114 | 3. Separated →GO TO 114

110. Is your (husband/partner) living with you now or is he staying elsewhere?

1. Living with her →GO TO 111 | 2. Living with her sometimes ↓ | 3. Staying elsewhere →GO TO 111

8. Do not Know ↓ | 9. No Response ↓

110B. HUSBAND/PARTNER [YES, MARRIED/YES, LIVING WITH]
During the month, how much time does your [husband / partner] live with you in this household?

1. 25% | 2. 50% | 3. 75%

110C. Where is his other home?

1. In this village | 2. In another village-specify (110CS. Specify (String 100))

111. Does your (husband / partner) have other wives or does he live with other women as if married?

1. Yes ↓ | 2. No →GO TO 114 | 8. Do not Know ↓ | 9. No Response ↓

112. Including yourself, in total, how many wives or live-in partners does he have?

- ENTER total number of wives and live-in partners
- If unknown, ENTER "88"
- If no response, ENTER "99"

00-99

113. Are you the first, second, ... wife?

- ENTER rank
- If rank unknown, ENTER "88"
- If no response, ENTER "99"

00-99

114. What is the highest level of school your (husband/partner) has completed:

- Primary 1 (1 cycle), primary 2 (2nd cycle), secondary (high school, technical) or higher?

1. Primary 1 (1st cycle)	2. Primary 2 (2nd cycle)	3. Secondary (high school / technical)	4. More than secondary
88. Do not Know	99. No Response		

115. What is your (husband's/partner's) occupation? That is, what kind of work does he mainly do?

1. Cultivator	2. Breeder	3. Trader	4. Butcher	5. Bricklayer	6. Official	7. Contractor
8. Other-specify (115S. Specify (String 100))	88. Do not Know	99. No Response				

130INTRO. As you know, some women take up jobs for which they are paid in cash or kind. Others sell things, have a small business or work on the family farm or in the family business.

- ENTER [1] to continue

1. Continue

130. During the past month, did you do any of these things or any other work for which you were paid in cash or in kind?

1. Yes	2. No	8. Do not Know	9. No Response

131. What is your occupation? That is, what kind of work do you mainly do?

1. Housewife | 2. Small business | 3. Trader | 4. Planter | 5. Official | 6. Contractor | 7. Other-specify (131S. Specify (String 100))

88. Do not Know | 99. No Response

132. Do you do this work for a member of your family, for someone else, or are you self-employed?

1. For family member | 2. For someone else | 3. Self-employed | 8. Do not Know | 9. No Response

133. Do you usually work throughout the year, or do you work seasonally, or only once in a while?

1. Throughout the year | 2. Seasonally / part of the year | 3. Once in a while | 8. Do not Know | 9. No Response

134A. Are you paid in cash or kind for this work or are you not paid at all?

1. Cash only →GO TO 134B | 2. Cash and kind →GO TO 134B | 3. In kind only →GO TO 134C | 4. Not paid →GO TO 136

8. Do not Know →GO TO 135 | 9. No Response →GO TO 135

134B. How much money do you get for this job?

- ENTER the value in FCFA:
- If value unknown, ENTER "8888"
- If no response, ENTER "9999"

0000-9999

134BCKPT.

1. If 134A=1 →GO TO 134D | 2. If 134A=2 OR 134A=3 ↓

134C. How much, in your estimation, is the cash value of this payment in kind?

- ENTER the value in FCFA:
- If value unknown, ENTER "8888"
- If no response, ENTER "9999"

0000-9999

134D. H[illegible]you paid?

1. Daily	2. Weekly	3. Monthly	4. Other-specify (134DS. Specify (String 100))	8. Do not Know	9. No Response

135. Who usually decides how the money you earn will be used?

1. Respondent	2. Husband/Partner	3. Concession leader	4. Respondent and husband/partner jointly	5. Respondent and concession leader jointly
6. Husband and concession leader jointly	7. Other-specify (135S. Specify (String 100))	88. Do not Know	99. No Response	

136. HUSBAND/PARTNER [YES, MARRIED/YES, LIVING WITH]
Who usually decides how your (husband's/partner's) earnings will be used?

1. Respondent	2. Husband/Partner	3. Concession leader	4. Respondent and husband/partner jointly	5. Respondent and concession leader jointly
6. Husband and concession leader jointly	7. Other-specify (136S. Specify (String 100))	88. Do not Know	99. No Response	

137. Who usually makes decisions about health care for yourself?

1. Respondent	2. Husband/Partner	3. Concession leader	4. Respondent and husband/partner jointly	5. Respondent and concession leader jointly
6. Husband and concession leader jointly	7. Other-specify (137S. Specify (String 100))	88. Do not Know	99. No Response	

138. Who usually makes decisions about making major household purchases?

1. Respondent	2. Husband/Partner	3. Concession leader	4. Respondent and husband/partner jointly	5. Respondent and concession leader jointly
6. Husband and concession leader jointly	7. Other-specify (138S. Specify (String 100))	88. Do not Know	99. No Response	

139. Who usually makes decisions about visits to your family or relatives?

1. Respondent	2. Husband/Partner	3. Concession leader	4. Respondent and husband/partner jointly	5. Respondent and concession leader jointly
6. Husband and concession leader jointly	7. Other-specify (139S. Specify (String 100))	88. Do not Know	99. No Response	

140. Sometimes a husband is upset or irritated by the things his wife does. In your opinion, is a husband justified in hitting or beating his wife in the following situations:

[A] If she goes out without telling him?

1. Yes	2. No	8. Do not Know	9. No Response

[B] If she neglects the children?

1. Yes	2. No	8. Do not Know	9. No Response

[C] If she argues with him?

1. Yes	2. No	8. Do not Know	9. No Response

[D] If she refuses to have sex with him?

1. Yes	2. No	8. Do not Know	9. No Response

[E] If she burns the food?

1. Yes	2. No	8. Do not Know	9. No Response

[F] If she uses contraceptives without the husband's consent?

1. Yes	2. No	8. Do not Know	9. No Response

[G] If she argues with the husband / partner's parents?

1. Yes	2. No	8. Do not Know	9. No Response

141. Have you ever:

- Been to the market?

1. Yes	2. No	8. Do not Know	9. No Response

- Been there alone?

1. Yes	2. No	8. Do not Know	9. No Response

- Been to the hospital/clinic/doctor?

1. Yes	2. No	8. Do not Know	9. No Response

- Gone there alone?

1. Yes	2. No	8. Do not Know	9. No Response

- Gone to a women's meeting?

1. Yes	2. No	8. Do not Know	9. No Response

- Gone there alone?

1. Yes	2. No	8. Do not Know	9. No Response

- Gone outside the village?

1. Yes	2. No	8. Do not Know	9. No Response

- Gone there alone?

1. Yes	2. No	8. Do not Know	9. No Response

Section 1B: CHW Care

142. Do you know how to contact or find your Community Health Worker in case of need?

1. Yes	2. No	3. I have no CHW	8. Do not Know	9. No Response

143. During the past month, have you met your community health worker for advice or care?

1. Yes	2. No	→GO TO 201	8. Do not Know	→GO TO 201	9. No Response	→GO TO 201

↓

144. If so, where did you meet the CHW?

1. At your house	2. At the CHW site	3. Other-specify (144S. Specify (String 100))	8. Do not Know	9. No Response

145. How many times?

- ENTER number of times
- If unknown number of times, ENTER "88"
- If no response, ENTER "99"

00-99

Section 2: Reproductive health
2A. Family planning

201. Are you or your partner currently doing something or using any method to delay or avoid getting pregnant?

1. Yes ↓ | 2. No →GO TO 218 | 8. Do not Know →GO TO 218 | 9. No Response →GO TO 218

202. What method are you using?

- RECORD ALL MENTIONED.

1. Female Sterilization | 2. Male Sterilization | 3. IUD | 4. Implants | 5. Injectables | 6. Pill | 7. Condom | 8. Female Condom

9. Diaphragm | 10. Foam / Jelly / Spermicidal Tablet | 11. Necklace (Standard Days Method) | 12. LAM (Lactational Amenorrhea Method)

13. Rhythm Method | 14. Withdrawal | 15. Other method-specify (202S. Specify (String 100)) | 88. Do not Know | 99. No Response

203. [CURRENT METHOD] = RESPONSE TO 202
Since what month and year have you been using [CURRENT METHOD] without stopping?

- Instruct the participant to estimate if she does not recall the exact date
- RECORD month
- If unknown, ENTER “88”
- If no response, ENTER “99”

01-12, 88, 99

- ENTER year
- If unknown, ENTER “8888”
- If no response, ENTER “9999”

1900-2100, 8888, 9999

204A. [CURRENT METHOD] = RESPONSE TO 202, [DATE 203] = RESPONSE TO 203
You started using [CURRENT METHOD] in [DATE 203]. Where did you get it at that time?

- If primary health center, SPECIFY which primary health center

1. At home →GO TO 205 | 2. National Hospital →GO TO 205 | 3. Regional Hospital →GO TO 205

4. Community Referral Hospital (Bankass) →GO TO 205 | 5. Primary health center-specify (204AS. Specify (String 100)) →GO TO 205

6. Community Health Worker →GO TO 204B | 7. Hospital / Private Clinic →GO TO 205 | 8. Shop →GO TO 205

9. Nightclub / kiosk / bar →GO TO 205 | 10. Street Vendor →GO TO 205 | 11. Acquaintance / Friend / Parent →GO TO 205

12. Other-specify (204AS. Specify (String 100)) →GO TO 205 | 88. Do not Know →GO TO 205 | 99. No Response →GO TO 205

204B. Where did you meet the CHW to get the method at that time?

1. At respondent's house | 2. CHW Site | 3. Other-specify (204BS. Specify (String 100)) | 8. Do not Know | 9. No Response

205. Has anyone referred you to this place for family planning?

1. Yes ↓ | 2. No →GO TO 207 | 8. Do not Know ↓ | 9. No Response ↓

206. If so, who referred you to this place?

- RECORD ALL MENTIONED

1. CHW | 2. A friend | 3. Husband | 4. Another member of the family | 5. A health worker from primary health center

6. Other-specify (206S. Specify (String 100)) | 88. Do not Know | 99. No Response

207. [CURRENT METHOD] = RESPONSE TO 202

Who advised you to use [CURRENT METHOD] family planning?

- RECORD ALL MENTIONED

1. Respondent | 2. Husband | 3. CHW | 4. Health worker at primary health center | 5. Night club / kiosk / bar

6. Street vendor | 7. Acquaintance / friend / parent | 8. Other-specify (207S. Specify (String 100)) | 88. Do not Know

99. No Response

208A. [CURRENT METHOD] = RESPONSE TO 202
Where did you get [CURRENT METHOD] last time?

- If primary health center, SPECIFY which primary health center

1. At home →GO TO 209
2. National Hospital →GO TO 209
3. Regional Hospital →GO TO 209
4. Community Referral Hospital (Bankass) →GO TO 209
5. Primary health center-specify (208AS. Specify (String 100)) →GO TO 209
6. Community Health Worker →GO TO 208B
7. Hospital / Private Clinic →GO TO 209
8. Shop →GO TO 209
9. Nightclub / kiosk / bar →GO TO 209
10. Street Vendor →GO TO 209
11. Acquaintance / Friend / Parent →GO TO 209
12. Other-specify (208AS. Specify (String 100)) →GO TO 209
88. Do not Know →GO TO 209
99. No Response →GO TO 209

208B. Where did you meet the CHW to get the method last time?

1. At respondent's house →GO TO 214
2. CHW Site ↓
3. Other-specify (208BS. Specify (String 100)) ↓
8. Do not Know ↓
9. No Response ↓

209. [CURRENT METHOD] = RESPONSE TO 202
In total, how much did you pay to get to the health center where you got [CURRENT METHOD] the last time?

- ENTER the value in silver
- If not paid, ENTER "7777"
- If unknown, ENTER "8888"
- If no response, ENTER "9999"

0000-9999

210. [CURRENT METHOD] = RESPONSE TO 202

Approximately how long it takes to get to the health center where you got [CURRENT METHOD] the last time?

1. Less than an hour (less than the time between fitri and saafo) | 2. 1 hour (time between fitri and saafo)

3. 2 hours (time between salifana and star) | 4. ½ day | 5. 1 day | 6. 2 or more days | 88. Do not Know | 99. No Response

211. How long does the time it takes to get to the health center seem to you?

1. Very long | 2. Long | 3. Normal | 4. Short | 5. Very short | 88. Do not Know | 99. No Response

212. Once at the health center, how long did you wait before receiving care?

1. Less than an hour (less than the time between fitri and saafo) | 2. 1 hour (time between fitri and saafo)

3. 2 hours (time between salifana and star) | 4. ½ day | 5. 1 day | 6. 2 or more days | 88. Do not Know | 99. No Response

213. How long does the waiting time seem to you?

1. Very long | 2. Long | 3. Normal | 4. Short | 5. Very short | 88. Do not Know | 99. No Response

214. How long does the family planning consultation take?

1. Less than an hour (less than the time between fitri and saafo) | 2. 1 hour (time between fitri and saafo)

3. 2 hours (time between salifana and star) | 4. ½ day | 5. 1 day | 6. 2 or more days | 88. Do not Know | 99. No Response

215. How much did you pay for the family planning method?

- ENTER value in silver:
- If not paid, ENTER "7777"
- If unknown, ENTER "8888"
- If no response, ENTER "9999"

0000-9999

216. In addition to what you told me, do you have other expenses related to this family planning method?

1. Yes ↓ | 2. No →GO TO 218 | 8. Do not Know →GO TO 218 | 9. No Response →GO TO 218

217. How much have you spent on: ?

A. Other medications

- ENTER value
- If unknown, ENTER "8888"
- If no response, ENTER "9999"

0000-9999

B. Food and accommodations

- ENTER value
- If unknown, ENTER "8888"
- If no response, ENTER "9999"

0000-9999

C. Other expenses

- ENTER value
- If unknown, ENTER "8888"
- If no response, ENTER "9999"

0000-9999

218. Over the last 12 months, were you interested in learning the methods of family planning?

1. Yes | 2. No | 8. Do not Know | 9. No Response

219. Have you consulted with someone to learn more?

1. Yes ↓ | 2. No →GO TO 221 | 8. Do not Know →GO TO 221 | 9. No Response →GO TO 221

220A. Who did you consult with to learn more?

1. Respondent →GO TO 221 | 2. Husband →GO TO 221 | 3. CHW →GO TO 220B

4. Health worker at primary health center →GO TO 221 | 5. Nightclub / kiosk / bar →GO TO 221

6. Street vendor → GO TO 221 | 7. Acquaintance / friend / parent →GO TO 221

8. Other-specify (220AS. Specify (String 100)) →GO TO 221 | 88. Do not Know →GO TO 221 | 99. No Response →GO TO 221

220B. Where did you consult with the CHW?

1. At respondent's house	2. CHW Site	3. Other-specify (220BS. Specify (String 100))	8. Do not Know	9. No Response

221. Over the past 12 months, did you receive a visit from a community health worker who told you about family planning?

1. Yes	2. No	→GO TO 228	8. Do not Know	→GO TO 228	9. No Response	→GO TO 228

↓

222. When was the last visit of the community health worker to talk about family planning?

- ENTER Value

0-50

- ENTER Unit

1. Days	2. Weeks	3. Months

223. What type of health worker visited you to talk about family planning.

- RECORD ALL MENTIONED

1. Doctor	2. Nurse	3. Midwife	4. Matron	5. CHW	6. Traditional birth attendant
7. Other-specify (223S. Specify (String 100))	88. Do not Know	99. No Response			

224. Did the health worker tell you about other family planning methods that you could use?

1. Yes	2. No	8. Do not Know	9. No Response

225. During the family planning consultation, did the health worker offer you a pregnancy test?

1. Yes	2. No	→GO TO 227	8. Do not Know	→GO TO 227	9. No Response	→GO TO 227

↓

226. If you have had a negative pregnancy test, did the health worker propose family planning?

1. Yes	2. No	3. I had a positive pregnancy test	8. Do not Know	9. No Response

227. Were you satisfied with the family planning services delivered by the health worker?

1. Not satisfied	2. Neutral	3. Satisfied	8. Do not Know	9. No Response

2B. Pregnancy and Childbirth

228. Are you pregnant now?

1. Yes	2. No	→GO TO 230	8. Do not Know	→GO TO 230	9. No Response	→GO TO 230

↓

229. How many months pregnant are you?

- RECORD NUMBER OF COMPLETED MONTHS.
- If unknown, ENTER “88”
- If no response, ENTER “99”

0-9, 88, 99

230. When did your last menstrual period start?

- Encourage the participant to make her best estimate if she does not know the exact number.
- If menopause or hysterectomy, ENTER “94”
- If before last birth, ENTER “95”
- If never menstruated, ENTER “96”
- If unknown, ENTER “88”
- If no response, ENTER “99”
- ENTER value

0-99

- ENTER unit

1. Days	2. Weeks	3. Months

231. What name was given to your last child?

- ENTER name of child

NAME (String 100)

232INTRO. [NAME] = RESPONSE TO 231
Now I want to ask you questions about your pregnancy with [NAME]

- ENTER [1] to continue

1. Continue

232. Did you have a test to confirm the pregnancy?

1. Yes ↓ | 2. No →GO TO 236 | 8. Do not Know →GO TO 236 | 9. No Response →GO TO 236

233. Who gave you the first test to confirm pregnancy?

1. Doctor | 2. Nurse | 3. Midwife | 4. Matron | 5. CHW | 6. Traditional birth attendant

7. Other-specify (233S. Specify (String 100)) | 88. Do not Know | 99. No Response

234. Where did you do your pregnancy test?

1. At home | 2. In a health center | 3. Other-specify (234S. Specify (String 100))

235. How much did you pay for your pregnancy test?

- ENTER value in FCFA
- If unknown, ENTER "8888"
- If no response, ENTER "9999"

0-9999

236. Did you see anyone for antenatal care for this pregnancy?

1. Yes ↓ | 2. No →GO TO 243 | 8. Do not Know →GO TO 243 | 9. No Response →GO TO 243

237. Whom did you see?

- Anyone else?
- PROBE TO IDENTIFY EACH TYPE OF PERSON AND RECORD ALL MENTIONED.

1. Doctor | 2. Nurse | 3. Midwife | 4. Matron | 5. CHW | 6. Traditional birth attendant

7. Other-specify (237S. Specify (String 100)) | 88. Do not Know | 99. No Response

238. Where did you receive antenatal care for this pregnancy?

- Anywhere else?
- PROBE TO IDENTIFY THE TYPE OF SOURCE.

1. Your home	2. Other house	3. National Hospital	4. Regional Hospital	5. District Hospital
6. Primary health center-specify (238S. Specify (String 100))	7. Clinic / Private Hospital	8. Pharmacy		
9. Other-specify (238S. Specify (String 100))	88. Do not Know	99. No Response		

239A. Who referred you to get antenatal care?

1. Respondent	→GO TO 240
2. Husband	→GO TO 240
3. CHW	→GO TO 239B
4. Health worker at primary health center	→GO TO 240
5. Nightclub / kiosk / bar	→GO TO 240
6. Street vendor	→GO TO 240
7. Acquaintance / friend / parent	→GO TO 240
8. Other-specify (239AS. Specify (String 100))	→GO TO 240
88. Do not Know	→GO TO 240
99. No Response	→GO TO 240

239B. Where was the reference by the CHW made?

1. At respondent's house	2. CHW Site	3. Other-specify (239BS. Specify (String 100))	8. Do not Know	9. No Response

240. How many months pregnant were you when you first received antenatal care for this pregnancy?

- ENTER value in months
- If unknown, ENTER "88"
- If no response, ENTER "99"

0-9, 88, 99

241. How many times did you receive antenatal care during this pregnancy?

- ENTER value
- If unknown, ENTER "88"
- If no response, ENTER "99"

0-99

242. During one of these prenatal visits, were you told about danger signs, warning signs, or complications in pregnancy?

1. Yes	2. No	8. Do not Know	9. No Response

243. During this pregnancy, did you take medicine to prevent malaria?

1. Yes	2. No	→GO TO 247	8. Do not Know	→GO TO 247	9. No Response	→GO TO 247
↓						

244. What medications did you take?

- RECORD ALL MENTIONED.
- IF TYPE OF MEDICINE IS NOT DETERMINED, SHOW ALL CURRENT ANTIMALARIAL IN THE SURVEY TO RESPONDENT.

1. SP / Fansidar	2. Chloroquine	3. Other-specify (244S. Specify (String 100))	88. Do not Know	99. No Response
↓	→GO TO 247			

245. How many times did you take SP/Fansidar during this pregnancy?

- ENTER number of times
- If unknown, ENTER "88"
- If no response, ENTER "99"

0-99

246. Did you get the SP / Fansidar under supervision of medical staff at the health center?

1. Yes	2. No	8. Do not Know	9. No Response

247. [NAME] = RESPONSE TO 231

Who assisted with the delivery of [NAME]?

- Anyone else ?
- PROBE FOR THE TYPE(S) OF PERSON(S) AND RECORD ALL MENTIONED.
- IF RESPONDENT SAYS NO ONE ASSISTED, PROBE TO DETERMINE WHETHER ANY ADULTS WERE PRESENT AT THE DELIVERY.

1. Doctor	2. Nurse	3. Midwife	4. Matron	5. CHW	6. Traditional birth attendant	7. Friend/Parent	8. Respondent (alone)

9. Other-specify (247S. Specify (String 100))	88. Do not Know	99. No Response

248. [NAME] = RESPONSE TO 231

Where did you give birth to [NAME]?

- PROBE TO IDENTIFY THE TYPE OF SOURCE.
- IF UNABLE TO DETERMINE IF PUBLIC OR PRIVATE SECTOR, WRITE THENAME OF THE PLACE

NAME OF PLACE (String 100)

- If at home, SPECIFY

1. Her home | 2. Other home

- If public sector, SPECIFY

3. National hospital | 4. Regional hospital | 5. Community referral hospital (Bankass) | 6. Maternity home

7. Primary Health Center | 8. Other public sector

- If private medical center, SPECIFY

9. Private Clinic / Hospital | 10. Office of private care | 11. Treatment Room | 12. Pharmacy | 13. Other private sector

- If other, SPECIFY

14. Transit | 15. Other-specify (248S. Specify (String 100)) | 88. Do not Know | 99. No Response

249A. Who referred you to this place for the birth?

- RECORD ALL MENTIONED

1. Respondent →GO TO 250 | 2. Husband →GO TO 250 | 3. CHW →GO TO 249B

4. Health worker at primary health center →GO TO 250 | 5. Nightclub / kiosk / bar →GO TO 250

6. Street vendor → GO TO 250 | 7. Acquaintance / friend / parent →GO TO 250

8. Other-specify (249AS. Specify (String 100)) →GO TO 250 | 88. Do not Know →GO TO 250 | 99. No Response →GO TO 250

249B. Where was the reference by the CHW made?

1. At respondent's house | 2. CHW Site | 3. Other-specify (249BS. Specify (String 100)) | 8. Do not Know | 9. No Response

250. [NAME] = RESPONSE TO 231

How long after (NAME) was delivered did you stay there?

- ENTER value
- If unknown, ENTER “88”
- If no response, ENTER “99”

0-99

- ENTER unit

1. Hours | 2. Days | 3. Weeks

251. [NAME] = RESPONSE TO 231
Was (NAME) delivered by caesarean, that is, did they cut your belly open to take the baby out?

1. Yes | 2. No | 8. Do not Know | 9. No Response

252. I would like to talk to you about checks on your health after delivery, for example, someone asking you questions about your health or examining you. Did anyone check on your health while you were still in the facility?

1. Yes ↓ | 2. No →GO TO 256 | 8. Do not Know ↓ | 9. No Response ↓

253. Who checked on your health at that time?

- RECORD ALL MENTIONED
- PROBE for most qualified person

1. Doctor | 2. Nurse | 3. Midwife | 4. Matron | 5. CHW | 6. Traditional birth attendant

7. Other-specify (253S. Specify (String 100)) | 88. Do not Know | 99. No Response

254. Where did the check take place?

- If primary health center, SPECIFY which one

1. Her home | 2. Other home | 3. National hospital | 4. Regional hospital | 5. Community referral hospital (Bankass) | 6. Maternity home

7. Primary health center-specify (254S. Specify (String 100)) | 8. Other public sector | 9. Private Clinic / Hospital | 10. Office of private care

11. Treatment Room | 12. Pharmacy | 13. Other private sector | 14. Transit | 15. Other-specify (254S. Specify (String 100))

88. Do not Know | 99. No Response

255. How long after delivery did the first check take place?

- ENTER value
- If unknown, ENTER “88”
- If no response, ENTER “99”

0-99

- ENTER unit

1. Hours	2. Days	3. Weeks

256. [NAME] = RESPONSE TO 231
In the two months after [NAME] was born, did a health care provider or a traditional birth attendant check on [NAME]’s health?

1. Yes, health professional (doctor, nurse, midwife) →GO TO 257
2. Yes, traditional midwife →GO TO 257
3. No →GO TO 260
8. Do not Know →GO TO 260
99. No Response →GO TO 260

257. [NAME] = RESPONSE TO 231
How long after delivery was [NAME]’s health first checked?

- ENTER value
- If unknown, ENTER “88”
- If no response, ENTER “99”

0-99

- ENTER unit

Hours	Days	Weeks

258. Who checked on (NAME)’s health at that time?

- RECORD ALL MENTIONED
- PROBE for most qualified person

1. Doctor	2. Nurse	3. Midwife	4. Matron	5. CHW	6. Traditional birth attendant
7. Other-specify (253S. Specify (String 100))	88. Do not Know	99. No Response			

259. Where did the check take place?

- If primary health center, SPECIFY which one

1. Her home | 2. Other home | 3. National hospital | 4. Regional hospital | 5. District Hospital

6. Primary health center-specify (259S. Specify (String 100)) | 7. Private Clinic / Hospital | 8. Pharmacy

9. Other-specify (259S. Specify (String 100)) | 88. Do not Know | 99. No Response

260. [NAME] = RESPONSE TO 231
Did you ever breastfeed [NAME]?

1. Yes ↓ | 2. No →GO TO 301 | 8. Do not Know →GO TO 301 | 9. No Response →GO TO 301

261. [NAME] = RESPONSE TO 231
Are you still breastfeeding [NAME]?

1. Yes | 2. No | 9. No Response

262. [NAME] = RESPONSE TO 231
If not, for how long did you breastfeed [NAME]?

- ENTER value

0-99

- ENTER unit

1. Day | 2. Month | 3. Year

263. For how long did you only breastfeed (NAME), that is to say, without other things to drink?

- ENTER value

0-99

- ENTER unit

1. Day | 2. Month | 3. Year

Section 3: Children, Survival and Mortality

301. Now I would like to ask about all the births you have had during your life. Have you ever given birth?

[1. Yes] [2. No] →GO TO 303

↓

302. How many of the children to whom you gave birth are still alive?

[0-20]

303. Have you ever given birth to a boy or girl who was born alive but later died?

- IF NO, PROBE: Any baby who cried, who made any movement, sound, or effort to breathe, or who showed any other signs of life even if for a very short time?

[1. Yes] [2. No] →GO TO 305 [9. No Response] →GO TO 305

↓

304. How many children have died?

[0-20]

305. ADD ANSWERS TO 302 AND 304, AND ENTER THE TOTAL.

- IF NONE, RECORD '00'.

[0-20]

306A. [_____] = RESPONSE TO 305

Just to make sure that I have this right: you have had in TOTAL [_____] births during your life. Is that correct?

[1. Yes] →GO TO 307 [2. No]

↓

306B. If Q306A = no, enter the correct number of total births

[0-20]

306C. If Q306A = no, enter the correct number of births of children still alive

[0-20]

306D. If Q306A = no, enter the correct number of children who died

0-20

307INTRO. Now I would like to record the names of all your births, whether still alive or not, starting with the first one you had.

- RECORD NAMES OF ALL THE BIRTHS IN 212.
- RECORD TWINS AND TRIPLETS AS SEPARATE.
- ENTER [1] to continue

1. Continue

Birth History Loop (Max = 30)

307. What name was given to your [first / next] baby?

- ENTER THE NAME.

NAME (String 100)

308. [NAME] = RESPONSE TO 307
On what day, month, and year was [NAME] born?

- ENTER day
- If unknown, ENTER "88"
- If no response, ENTER "99"

01-31, 88, 99

- ENTER month
- If unknown, ENTER "88"
- If no response, ENTER "99"

01-12, 88, 99

- ENTER year
- If unknown, ENTER "8888"
- If no response, ENTER "9999"

1900-2100, 8888, 9999

309. [NAME] = RESPONSE TO 307
Is [NAME] a boy or girl?

1. Boy	2. Girl

310. [NAME] = RESPONSE TO 307
Is [NAME] still alive?

1. Yes	2. No	→GO TO 312

↓

311. [NAME] = RESPONSE TO 307
IF ALIVE: How old was [NAME] at [NAME]'s last birthday?

- Enter the age in years, months and days.
- All fields are required.
- Mark 00 years if the child is less than one year, 00 months if the child is less than one month
- ENTER years:
- If unknown, ENTER "88"
- If no response, ENTER "99"

0-40, 88, 99

- ENTER months:
- If unknown, ENTER "88"
- If no response, ENTER "99"

0-12, 88, 99

- ENTER days:
- If unknown, ENTER "88"
- If no response, ENTER "99"

0-31, 88, 99

312. [NAME] = RESPONSE TO 307
IF DECEASED: What was the date of death of [NAME]?

- Mark MONTH and YEAR. The two fields are mandatory
- ENTER month as “MM”
- If unknown, ENTER “88”
- If no response, ENTER “99”

01-12, 88, 99

- ENTER year as “YYYY”
- If unknown, ENTER “8888”
- If no response, ENTER “9999”

1900-2100, 8888, 9999

313. [NAME] = RESPONSE TO 307, HE/SHE = [BOY / GIRL]
IF DECEASED: How old was [NAME] when [he / she] died?

- Enter the age in years, months and days.
- All fields are required.
- Mark 00 years if the child was less than one year, 00 months if the child was less than one month
  - ENTER years:
  - If unknown, ENTER “88”
  - If no response, ENTER “99”

0-40, 88, 99

  - ENTER months:
  - If unknown, ENTER “88”
  - If no response, ENTER “99”

0-12, 88, 99

  - ENTER days:
  - If unknown, ENTER “88”
  - If no response, ENTER “99”

0-31, 88, 99

314. [NAME OF PREVIOUS BIRTH] = RESPONSE TO 307_X-1 [NAME] = RESPONSE TO 307_X
Were there any other live births between [NAME OF PREVIOUS BIRTH] and [NAME], including any children who died after birth?

1. Yes	2. No

End of Birth History Loop

315CKPT. WHETHER NUMBER OF BIRTHS IS CORRECT

1. RECORDED NUMBER OF BIRTHS IS INCONSISTENT WITH LOOPED RESPONSES ↓	2. RECORDED NUMBER OF BIRTHS IN TABLE IS CONSISTENT WITH RESPONSES IN LOOP	→ GO TO 301

COMPARE WITH THE NUMBER OF BIRTHS RECORDED IN THE TABLE ABOVE AND MARK:
NUMBERS ARE EQUAL? DIFFERENT (PROBE AND CORRECT)?

Section 4: Diseases Of Children

Diarrhea

401. [NAME]
Has [NAME] had diarrhea in the last 2 weeks?

1. Yes | 2. No →GO TO 500 | 8. Do not Know →GO TO 500 | 9. No Response →GO TO 500

402. Was there blood in the stool?

1. Yes | 2. No | 8. Do not Know | 9. No Response

403. Did you seek advice or treatment for the diarrhea from any source?

1. Yes →GO TO 405 | 2. No ↓ | 8. Do not Know ↓ | 9. No Response ↓

404. Why did you not get advice or treatment?

- PROBE: Other reasons?
- RECORD ALL MENTIONED

1. I could not pay the fee
2. I could not cross the distance to the health center
3. I do not make decisions about seeking health care
4. I arrived at the health center, but there was no equipment, material, or infrastructure to give me care
5. The prescribed medication was not available
6. I arrived at the health center, but I did not find staff qualified enough to give me care
7. I did not know what to do
8. I did not think it was serious enough
9. I did not have enough time
10. Other-specify (404S. Specify (String 100))
88. Do not Know
99. No Response

→GO TO 500

405. Where did you seek advice or treatment? Please list all sources in the order that you got them.

- PROBE: Anywhere else?
- PROBE TO IDENTIFY THE SOURCE
- If primary health center, SPECIFY which one

1. At my house | 2. National hospital | 3. Regional hospital | 4. Community referral hospital (Bankass)

5. Primary health center-specify (405S. Specify (String 100)) | 6. Private Clinic / Hospital | 7. Pharmacy | 8. At CHW | 9. Home of CHW

10. At a traditional healer | 11. At home, a travelling seller | 12. Shop | 13. Other-specify (405S. Specify (String 100))

88. Do not Know | 99. No Response

406. HE/SHE = [BOY / GIRL]

Did someone evaluate the child before [he / she] went to the health center? If so, who was it?

0. No →GO TO 410 | 1. Myself →GO TO 410 | 2. Husband →GO TO 410 | 3. Other women →GO TO 410

4. Other close relatives →GO TO 410 | 5. Friends / neighbors →GO TO 410 | 6. CHW →GO TO 407 | 7. Doctor →GO TO 410

8. Nurse →GO TO 410 | 9. Midwife →GO TO 410 | 10. Matron →GO TO 410 | 11. Traditional healer →GO TO 410

12. Drug seller →GO TO 410 | 88. Do not Know →GO TO 410 | 99. No Response →GO TO 410

407. Where was the evaluation conducted?

1. At home →GO TO 408 | 2. CHW Site →GO TO 409 | 3. Other-specify (407S. Specify (String 100)) →GO TO 409

8. Do not Know →GO TO 409 | 9. No Response →GO TO 409

408. How much time does it seem to take the CHW to arrive at your house?

1. Very long | 2. Long | 3. Normal | 4. Short | 5. Very short | 88. Do not Know | 99. No Response

409. How long did the evaluation last?

1. Less than an hour (less than the time between fitri and saafo)	2. 1 hour (time between fitri and saafo)	3. 2 hours (time between salifana and star)

4. ½ day	5. 1 day	6. 2 or more days	88. Do not Know	99. No Response

409CKPT.

1. If 405=2-7 OR 10-13 ↓	2. If 405=1, 8, 9, 88 OR 99 →GO TO 414

410. How much time did it seem to take to get to the health center?

- If you went to several health centers, this is the last facility you attended.

1. Very long	2. Long	3. Normal	4. Short	5. Very short	88. Do not Know	99. No Response

411. At the health center, how long did it take before you received treatment?

1. Less than an hour (less than the time between fitri and saafo)	2. 1 hour (time between fitri and saafo)

3. 2 hours (time between salifana and star)	4. ½ day	5. 1 day	6. 2 or more days	88. Do not Know	99. No Response

412. How did the waiting time seem to you?

1. Very long	2. Long	3. Normal	4. Short	5. Very short	88. Do not Know	99. No Response

413. How long did the medical consultation take?

1. Less than an hour (less than the time between fitri and saafo)	2. 1 hour (time between fitri and saafo)

3. 2 hours (time between salifana and star)	4. ½ day	5. 1 day	6. 2 or more days	88. Do not Know	99. No Response

414. Did you take time off work to bring your child to the clinic? That is to say, the work you do that generates income. If so, how many days?

- If no leave taken, ENTER 0
- If unknown, ENTER 88
- If no response, ENTER 99

0-99

415. Wh[illegible]g care for your child, which of the following expenses did you obtain?

1. Medication / treatment

1. Yes	2. No	8. Do not Know	9. No Response

2. Laboratory / diagnostic tests

1. Yes	2. No	8. Do not Know	9. No Response

3. Other health expenses

1. Yes	2. No	8. Do not Know	9. No Response

4. Food / Accommodation

1. Yes	2. No	8. Do not Know	9. No Response

5. Other expenses

1. Yes	2. No	8. Do not Know	9. No Response

415CKPT.

1. If ALL 415==1 ↓	2. If ANY 415==2, 8, or 9	→GO TO 417

416. If Yes: How much did you pay for all these things?

- ENTER value in FCFA
- If unknown, ENTER 8888
- If no response, ENTER 9999

0-9999

417. What treatment did your child receive?

- RECORD ALL MENTIONED

1. ORS	2. Antibiotic tablet or syrup	3. Antimotility tablet or syrup	4. Zinc tablet or syrup
5. Other / unknown tablet or syrup	6. Injectable antibiotic	7. Non-injectable antibiotic	8. Unknown injection
9. (IV) Intravenous	10. Home remedy / herbal medicine	11. Other-specify (417S. Specify (String 100))	
88. Do not Know	99. No Response		

418a A

418a_1. [NAME]

How long after the onset of diarrhea, did [NAME] start taking the ORS?

0. Same day | 1. Next day | 2. Two days after diarrhea | 3. Three days or more after diarrhea | 88. Do not Know | 99. No Response

418a_2. [NAME]
How long after the onset of diarrhea, did [NAME] start taking the antibiotic tablet or syrup?

0. Same day | 1. Next day | 2. Two days after diarrhea | 3. Three days or more after diarrhea | 88. Do not Know | 99. No Response

418a_3. [NAME]
How long after the onset of diarrhea, did [NAME] start taking the antimotility tablet or syrup?

0. Same day | 1. Next day | 2. Two days after diarrhea | 3. Three days or more after diarrhea | 88. Do not Know | 99. No Response

418a_4. [NAME]
How long after the onset of diarrhea, did [NAME] start taking the zinc tablet or syrup?

0. Same day | 1. Next day | 2. Two days after diarrhea | 3. Three days or more after diarrhea | 88. Do not Know | 99. No Response

418a_5. [NAME]
How long after the onset of diarrhea, did [NAME] start taking the other / unknown tablet or syrup?

0. Same day | 1. Next day | 2. Two days after diarrhea | 3. Three days or more after diarrhea | 88. Do not Know | 99. No Response

418a_6. [NAME]
How long after the onset of diarrhea, did [NAME] start taking the injectable antibiotic?

0. Same day | 1. Next day | 2. Two days after diarrhea | 3. Three days or more after diarrhea | 88. Do not Know

99. No Response

418a_7. [NAME]
How long after the onset of diarrhea, did [NAME] start taking the non-injectable antibiotic?

0. Same day | 1. Next day | 2. Two days after diarrhea | 3. Three days or more after diarrhea | 88. Do not Know
99. No Response

418a_8. [NAME]
How long after the onset of diarrhea, did [NAME] start taking the unknown injection?

0. Same day | 1. Next day | 2. Two days after diarrhea | 3. Three days or more after diarrhea | 88. Do not Know
99. No Response

418a_9. [NAME]
How long after the onset of diarrhea, did [NAME] start taking the (IV) intraveneous drug?

0. Same day | 1. Next day | 2. Two days after diarrhea | 3. Three days or more after diarrhea | 88. Do not Know
99. No Response

418a_10. [NAME]
How long after the onset of diarrhea, did [NAME] start taking the home remedy / herbal medicine?

0. Same day | 1. Next day | 2. Two days after diarrhea | 3. Three days or more after diarrhea | 88. Do not Know
99. No Response

418a_11. [NAME]
How long after the onset of diarrhea, did [NAME] start taking the other drug?

0. Same day | 1. Next day | 2. Two days after diarrhea | 3. Three days or more after diarrhea | 88. Do not Know
99. No Response

418a_88[illegible]
How long after the onset of diarrhea, did [NAME] start taking __________?

0. Same day | 1. Next day | 2. Two days after diarrhea | 3. Three days or more after diarrhea | 88. Do not Know

99. No Response

418a_99. [NAME]
How long after the onset of diarrhea, did [NAME] start taking __________?

0. Same day | 1. Next day | 2. Two days after diarrhea | 3. Three days or more after diarrhea | 88. Do not Know

99. No Response

Fever

500. Has [NAME] been ill with a fever at any time during the last 2 weeks?

1. Yes | 2. No →GO TO 600 | 8. Do not Know →GO TO 600 | 9. No Response →GO TO 600

501. Did you seek advice or treatment for the fever from any source?

1. Yes →GO TO 503 | 2. No | 8. Do not Know →GO TO 503 | 9. No Response →GO TO 503

502. Why did you not get advice or treatment?

- PROBE: Other reasons?
- RECORD ALL MENTIONED

1. I could not pay the fee | 2. I could not cross the distance to the health center | 3. I do not make decisions about seeking health care

4. I arrived at the health center, but there was no equipment, material, or infrastructure to give me care

5. The prescribed medication was not available | 6. I arrived at the health center, but I did not find staff qualified enough to give me care

7. I did not know what to do | 8. I did not think it was serious enough | 9. I did not have enough time

10. Other-specify (502S. Specify (String 100)) | 88. Do not Know | 99. No Response

→GO TO 600

503. Where did you seek advice or treatment? Please list all sources in the order that you got them.

- PROBE: Anywhere else?
- PROBE TO IDENTIFY THE SOURCE
- If primary health center, SPECIFY which one

1. At my house | 2. National hospital | 3. Regional hospital | 4. Community referral hospital (Bankass)

5. Primary health center-specify (503S. Specify (String 100)) | 6. Private Clinic / Hospital | 7. Pharmacy | 8. At CHW | 9. Home of CHW

10. At a traditional healer | 11. At home, a travelling seller | 12. Shop | 13. Other-specify (503S. Specify (String 100))

88. Do not Know | 99. No Response

504. At [illegible] blood taken from (NAME)'s finger or heel for testing? If so, who took the blood?

0. No | 1. Myself | 2. Husband | 3. Other women | 4. Other close relatives | 5. Friends / neighbors
6. CHW | 7. Doctor | 8. Nurse | 9. Midwife | 10. Matron | 11. Traditional healer | 12. Drug seller
88. Do not Know | 99. No Response

505. Did someone evaluate the child before (he/she) went to the health center? If so, who was it?

0. No →GO TO 509 | 1. Myself →GO TO 509 | 2. Husband →GO TO 509 | 3. Other women →GO TO 509
4. Other close relatives →GO TO 509 | 5. Friends / neighbors →GO TO 509 | 6. CHW →GO TO 506 | 7. Doctor →GO TO 509
8. Nurse →GO TO 509 | 9. Midwife →GO TO 509 | 10. Matron →GO TO 509 | 11. Traditional healer →GO TO 509
12. Drug seller →GO TO 509 | 88. Do not Know →GO TO 509 | 99. No Response →GO TO 509

506. Where was the evaluation conducted?

1. At home →GO TO 507 | 2. CHW Site →GO TO 508 | 3. Other-specify (506S. Specify (String 100)) →GO TO 508
8. Do not Know →GO TO 508 | 9. No Response →GO TO 508

507. How much time does it seem to take the CHW to arrive at your house?

1. Very long | 2. Long | 3. Normal | 4. Short | 5. Very short | 88. Do not Know | 99. No Response

508. How long did the evaluation last?

1. Less than an hour (less than the time between fitri and saafo) | 2. 1 hour (time between fitri and saafo)
3. 2 hours (time between salifana and star) | 4. ½ day | 5. 1 day | 6. 2 or more days | 88. Do not Know | 99. No Response

508CKPT.

1. If 503=2-7 OR 10-13 ↓ | 2. If 503=1, 8, 9, 88 OR 99 →GO TO 513

509. How much time did it seem to take to get to the health center?

- If you went to several health centers, this is the last facility you attended.

1. Very long	2. Long	3. Normal	4. Short	5. Very short	88. Do not Know	99. No Response

510. At the health center, how long did it take before you received treatment?

1. Less than an hour (less than the time between fitri and saafo)	2. 1 hour (time between fitri and saafo)

3. 2 hours (time between salifana and star)	4. ½ day	5. 1 day	6. 2 or more days	88. Do not Know	99. No Response

511. How did the waiting time seem to you?

1. Very long	2. Long	3. Normal	4. Short	5. Very short	88. Do not Know	99. No Response

512. How long did the medical consultation take?

1. Less than an hour (less than the time between fitri and saafo)	2. 1 hour (time between fitri and saafo)

3. 2 hours (time between salifana and star)	4. ½ day	5. 1 day	6. 2 or more days	88. Do not Know	99. No Response

513. Did you take time off work to bring your child to the clinic? That is to say, the work you do that generates income. If so, how many days?

- If no leave taken, ENTER 0
- If unknown, ENTER 88
- If no response, ENTER 99

0-99

514. While seeking care for your child, which of the following expenses did you obtain?

1. Medication / treatment

1. Yes | 2. No | 8. Do not Know | 9. No Response

2. Laboratory / diagnostic tests

1. Yes | 2. No | 8. Do not Know | 9. No Response

3. Other health expenses

1. Yes | 2. No | 8. Do not Know | 9. No Response

4. Food / Accommodation

1. Yes | 2. No | 8. Do not Know | 9. No Response

5. Other expenses

1. Yes | 2. No | 8. Do not Know | 9. No Response

514CKPT.

1. If ALL 514==1 ↓ | 2. If ANY 514==2, 8, or 9 →GO TO 516

515. If Yes: How much did you pay for all these things?

- ENTER value in FCFA
- If unknown, ENTER 8888
- If no response, ENTER 9999

0-9999

516. What treatment did your child receive?

- RECORD ALL MENTIONED

1. SP / Fansidar | 2. Chloroquine | 3. Amodiaquine | 4. Artemisinin Combination Therapy (ACT) | 5. Quinine tablet | 6. Quinine injection

7. Artesunate injection | 8. Artemether injection | 9. Other antipaludienne-specify (516S. Specify (String 100)) | 10. Paracetamol | 11. Ibuprofen

11. Other-specify (516S. Specify (String 100)) | 88. Do not Know | 99. No Response

Fever Medication Loop

517a. [NAME], [INSERT DRUG 516] = RESPONSE TO 516

How long after the onset of fever, did (NAME) start taking the [INSERT DRUG 516]?

- Ask this question for all medications selected in Q516

0. Same day	1. Next day	2. Two days after fever	3. Three days or more after fever	88. Do not Know
99. No Response				

517aCKPT.

1. No more medications selected in 516	2. Another medication selected in 516	→ GO TO 517a
↓		

End of Loop

Cough / Difficulty Breathing

600. [NAME]
Has [NAME] been ill with a cough at any time during the last 2 weeks?

1. Yes | 2. No | 8. Do not Know | 9. No Response

601. [NAME]
When [NAME] was ill with a cough, did [NAME] have fast, short, rapid breaths or difficulty breathing at any time?

1. Yes | 2. No | 8. Do not Know | 9. No Response

602. Did you seek advice or treatment for the diarrhea from any source?

1. Yes →GO TO 604 | 2. No ↓ | 8. Do not Know ↓ | 9. No Response ↓

603. Why did you not get advice or treatment?

- PROBE: Other reasons?
- RECORD ALL MENTIONED

1. I could not pay the fee
2. I could not cross the distance to the health center
3. I do not make decisions about seeking health care
4. I arrived at the health center, but there was no equipment, material, or infrastructure to give me care
5. The prescribed medication was not available
6. I arrived at the health center, but I did not find staff qualified enough to give me care
7. I did not know what to do
8. I did not think it was serious enough
9. I did not have enough time
10. Other-specify (603S. Specify (String 100))
88. Do not Know
99. No Response

→GO TO 700a

604. Where did you seek advice or treatment? Please list all sources in the order that you got them.

- PROBE: Anywhere else?
- PROBE TO IDENTIFY THE SOURCE
- If primary health center, SPECIFY which one

1. At my house | 2. National hospital | 3. Regional hospital | 4. Community referral hospital (Bankass)

5. Primary health center-specify (604S. Specify (String 100)) | 6. Private Clinic / Hospital | 7. Pharmacy | 8. At CHW | 9. Home of CHW

10. At a traditional healer | 11. At home, a travelling seller | 12. Shop | 13. Other-specify (604S. Specify (String 100)) | 88. Do not Know

99. No Response

605. HE/SHE = [BOY / GIRL]

Did someone evaluate the child before [he / she] went to the health center? If so, who was it?

0. No →GO TO 609 | 1. Myself →GO TO 609 | 2. Husband →GO TO 609 | 3. Other women →GO TO 609

4. Other close relatives →GO TO 609 | 5. Friends / neighbors →GO TO 609 | 6. CHW →GO TO 606 | 7. Doctor →GO TO 609

8. Nurse →GO TO 609 | 9. Midwife →GO TO 609 | 10. Matron →GO TO 609 | 11. Traditional healer →GO TO 609

12. Drug seller →GO TO 609 | 88. Do not Know →GO TO 609 | 99. No Response →GO TO 609

606. Where was the evaluation conducted?

1. At home ↓ | 2. CHW Site →GO TO 608 | 3. Other-specify (606S. Specify (String 100)) ↓ | 8. Do not Know ↓ | 9. No Response ↓

607. How much time does it seem to take the CHW to arrive at your house?

1. Very long | 2. Long | 3. Normal | 4. Short | 5. Very short | 88. Do not Know | 99. No Response

608. How long did the evaluation last?

1. Less than an hour (less than the time between fitri and saafo) | 2. 1 hour (time between fitri and saafo)

3. 2 hours (time between salifana and star) | 4. ½ day | 5. 1 day | 6. 2 or more days | 88. Do not Know | 99. No Response

608CKPT.

1. If 604=2-7 OR 10-13 ↓	2. If 604=1, 8, 9, 88 OR 99	→GO TO 613

609. How much time did it seem to take to get to the health center?
   - If you went to several health centers, this is the last facility you attended.

1. Very long	2. Long	3. Normal	4. Short	5. Very short	88. Do not Know	99. No Response

610. At the health center, how long did it take before you received treatment?

1. Less than an hour (less than the time between fitri and saafo)	2. 1 hour (time between fitri and saafo)

3. 2 hours (time between salifana and star)	4. ½ day	5. 1 day	6. 2 or more days	88. Do not Know	99. No Response

611. How did the waiting time seem to you?

1. Very long	2. Long	3. Normal	4. Short	5. Very short	88. Do not Know	99. No Response

612. How long did the medical consultation take?

1. Less than an hour (less than the time between fitri and saafo)	2. 1 hour (time between fitri and saafo)

3. 2 hours (time between salifana and star)	4. ½ day	5. 1 day	6. 2 or more days	88. Do not Know	99. No Response

613. Did you take time off work to bring your child to the clinic? That is to say, the work you do that generates income. If so, how many days?
- If no leave taken, ENTER 0
- If unknown, ENTER 88
- If no response, ENTER 99

0-99

614. While seeking care for your child, which of the following expenses did you obtain?
   1. Medication / treatment

1. Yes	2. No	8. Do not Know	9. No Response

2. Laboratory / diagnostic tests

1. Yes	2. No	8. Do not Know	9. No Response

3. Other health expenses

1. Yes	2. No	8. Do not Know	9. No Response

4. Food / Accommodation

1. Yes	2. No	8. Do not Know	9. No Response

5. Other expenses

1. Yes	2. No	8. Do not Know	9. No Response

614CKPT.

1. If ALL 614==1 ↓	2. If ANY 614==2, 8, or 9 →GO TO 616

615. If Yes: How much did you pay for all these things?

- ENTER value in FCFA
- If unknown, ENTER 8888
- If no response, ENTER 9999

0-9999

616. What treatment did your child receive?

- RECORD ALL MENTIONED

1. Amoxicillin	2. Another antibiotic-specify (616S. Specify (String 100))	2. Antitussive
3. Decongestant (taste in the nostrils)	4. Local applications (ie, cream, ointment, butter)	5. Antimalarial
6. Paracetamol	7. Ibuprofen	8. Other-specify (616S. Specify (String 100))
88. Do not Know	99. No Response	

617a. 6
617a_1. [NAME]
How long after the onset of diarrhea, did [NAME] start taking the amoxicillin?

0. Same day	1. Next day	2. Two days after diarrhea	3. Three days or more after diarrhea	88. Do not Know	99. No Response

617a_2. [NAME]
How long after the onset of diarrhea, did [NAME] start taking the other antibiotic?

0. Same day	1. Next day	2. Two days after diarrhea	3. Three days or more after diarrhea	88. Do not Know	99. No Response

617a_2. [NAME]
How long after the onset of diarrhea, did [NAME] start taking the antitussive?

0. Same day	1. Next day	2. Two days after diarrhea	3. Three days or more after diarrhea	88. Do not Know	99. No Response

617a_3. [NAME]
How long after the onset of diarrhea, did [NAME] start taking the decongestant?

0. Same day	1. Next day	2. Two days after diarrhea	3. Three days or more after diarrhea	88. Do not Know	99. No Response

617a_4. [NAME]
How long after the onset of diarrhea, did [NAME] start taking the local application?

0. Same day	1. Next day	2. Two days after diarrhea	3. Three days or more after diarrhea	88. Do not Know	99. No Response

617a_5. [NAME]
How long after the onset of diarrhea, did [NAME] start taking the antimalarial?

0. Same day	1. Next day	2. Two days after diarrhea	3. Three days or more after diarrhea	88. Do not Know	99. No Response

617a_6. [NAME]
How long after the onset of diarrhea, did [NAME] start taking the paracetamol?

0. Same day	1. Next day	2. Two days after diarrhea	3. Three days or more after diarrhea	88. Do not Know	99. No Response

617a_7. [NAME]
How long after the onset of diarrhea, did [NAME] start taking the ibuprofen?

0. Same day	1. Next day	2. Two days after diarrhea	3. Three days or more after diarrhea	88. Do not Know	99. No Response

617a_8. [NAME]
How long after the onset of diarrhea, did [NAME] start taking the other drug?

0. Same day	1. Next day	2. Two days after diarrhea	3. Three days or more after diarrhea	88. Do not Know	99. No Response

617a_88. [NAME]
How long after the onset of diarrhea, did [NAME] start taking the ________?

0. Same day	1. Next day	2. Two days after diarrhea	3. Three days or more after diarrhea	88. Do not Know	99. No Response

617a_99. [NAME]
How long after the onset of diarrhea, did [NAME] start taking the ________?

0. Same day	1. Next day	2. Two days after diarrhea	3. Three days or more after diarrhea	88. Do not Know	99. No Response

Other diseases

700a. [NAME]
Has [NAME] had any other types of illnesses or injuries the last two weeks?

1. Yes	2. No	8. Do not Know	9. No Response

700b. If so, what type of illness or injury?

1. Skin infection (skin)	2. Intestinal parasite	3. Bilharziose (blood in urine)	4. Conjunctivitis (eye infection)
5. Fractures (bone)	6. Wounds	7. Other-specify (700bS. Specify (String 100))	

701. Did you seek advice or treatment for the diarrhea from any source?

1. Yes →GO TO 703	2. No ↓	8. Do not Know ↓	9. No Response ↓

702. Why did you not get advice or treatment?

- PROBE: Other reasons?
- RECORD ALL MENTIONED

1. I could not pay the fee	2. I could not cross the distance to the health center	3. I do not make decisions about seeking health care
4. I arrived at the health center, but there was no equipment, material, or infrastructure to give me care		
5. The prescribed medication was not available	6. I arrived at the health center, but I did not find staff qualified enough to give me care	
7. I did not know what to do	8. I did not think it was serious enough	9. I did not have enough time
10. Other-specify (702S. Specify (String 100))	88. Do not Know	99. No Response

→GO TO 800

703. Where did you seek advice or treatment? Please list all sources in the order that you got them.

- PROBE: Anywhere else?
- PROBE TO IDENTIFY THE SOURCE
- If primary health center, SPECIFY which one

1. At my house	2. National hospital	3. Regional hospital	4. Community referral hospital (Bankass)
5. Primary health center-specify (703S. Specify (String 100))	6. Private Clinic / Hospital	7. Pharmacy	8. At CHW	9. Home of CHW
10. At a traditional healer	11. At home, a travelling seller	12. Shop	13. Other-specify (703S. Specify (String 100))	88. Do not Know
99. No Response

704. HE/SHE = [BOY / GIRL]
Did someone evaluate the child before [he/she] went to the health center? If so, who was it?

0. No →GO TO 708	1. Myself →GO TO 708	2. Husband →GO TO 708	3. Other women →GO TO 708
4. Other close relatives →GO TO 708	5. Friends / neighbors →GO TO 708	6. CHW →GO TO 705	7. Doctor →GO TO 708
8. Nurse →GO TO 708	9. Midwife →GO TO 708	10. Matron →GO TO 708	11. Traditional healer →GO TO 708
12. Drug seller →GO TO 708	88. Do not Know →GO TO 708	99. No Response →GO TO 708

705. Where was the evaluation conducted?

| 1. At home ↓ | 2. CHW Site →GO TO 707 | 3. Other-specify (705S. Specify (String 100)) ↓ | 8. Do not Know ↓ | 9. No Response ↓ |

706. How much time does it seem to take the CHW to arrive at your house?

| 1. Very long | 2. Long | 3. Normal | 4. Short | 5. Very short | 88. Do not Know | 99. No Response |

707. How long did the evaluation last?

| 1. Less than an hour (less than the time between fitri and saafo) | 2. 1 hour (time between fitri and saafo) |
| 3. 2 hours (time between salifana and star) | 4. ½ day | 5. 1 day | 6. 2 or more days | 88. Do not Know | 99. No Response |

70

1. If 703=2-7 OR 10-13 ↓	2. If 703=1, 8, 9, 88 OR 99 →GO TO 712

708. How much time did it seem to take to get to the health center?
   - If you went to several health centers, this is the last facility you attended.

1. Very long	2. Long	3. Normal	4. Short	5. Very short	88. Do not Know	99. No Response

709. At the health center, how long did it take before you received treatment?

1. Less than an hour (less than the time between fitri and saafo)	2. 1 hour (time between fitri and saafo)

3. 2 hours (time between salifana and star)	4. ½ day	5. 1 day	6. 2 or more days	88. Do not Know	99. No Response

710. How did the waiting time seem to you?

1. Very long	2. Long	3. Normal	4. Short	5. Very short	88. Do not Know	99. No Response

711. How long did the medical consultation take?

1. Less than an hour (less than the time between fitri and saafo)	2. 1 hour (time between fitri and saafo)

3. 2 hours (time between salifana and star)	4. ½ day	5. 1 day	6. 2 or more days	88. Do not Know	99. No Response

712. Did you take time off work to bring your child to the clinic? That is to say, the work you do that generates income. If so, how many days?

- If no leave taken, ENTER 0
- If unknown, ENTER 88
- If no response, ENTER 99

0-99

713. While seeking care for your child, which of the following expenses did you obtain?

1. Medication / treatment

1. Yes | 2. No | 8. Do not Know | 9. No Response

2. Laboratory / diagnostic tests

1. Yes | 2. No | 8. Do not Know | 9. No Response

3. Other health expenses

1. Yes | 2. No | 8. Do not Know | 9. No Response

4. Food / Accommodation

1. Yes | 2. No | 8. Do not Know | 9. No Response

5. Other expenses

1. Yes | 2. No | 8. Do not Know | 9. No Response

713CKPT.

1. If ALL 713==1 ↓ | 2. If ANY 713==2, 8, or 9 →GO TO 715

714. If Yes: How much did you pay for all these things?

- ENTER value in FCFA
- If unknown, ENTER 8888
- If no response, ENTER 9999

0-9999

715. What treatment did your child receive?

- RECORD ALL MENTIONED

1. Antibiotic | 2. Antimalarial | 3. Paracetamol | 4. Ibuprofen | 5. Medicinal Plant

6. Another medicine-specify (715S. Specify (String 100)) | 88. Do not Know | 99. No Response

Illness or Injury Medication Loop

716a. [NAME], [INSERT DRUG 715] = RESPONSE TO 715

How long after the illness or injury started did [NAME] start taking the [INSERT DRUG 715]?

- Ask this question for all medications selected in Q715

0. Same day	1. Next day	2. Two days after diarrhea	3. Three days or more after diarrhea	88. Do not Know
99. No Response				

716aCKPT.

1. No more medications selected in 715 ↓	2. Another medication selected in 715	→ GO TO 716a

End of Loop

Access to essential care for the respondent

800. Have you had an illness or injury in the last six months?

- Clarification: unrelated to pregnancy or childbirth

1. Yes ↓ | 2. No →END OF INTERVIEW | 8. Do not Know →END OF INTERVIEW | 9. No Response →END OF INTERVIEW

801. Did you seek advice or treatment for the diarrhea from any source?

1. Yes →GO TO 803 | 2. No ↓ | 8. Do not Know ↓ | 9. No Response ↓

802. Why did you not get advice or treatment?

- PROBE: Other reasons?
- RECORD ALL MENTIONED

1. I could not pay the fee
2. I could not cross the distance to the health center
3. I do not make decisions about seeking health care
4. I arrived at the health center, but there was no equipment, material, or infrastructure to give me care
5. The prescribed medication was not available
6. I arrived at the health center, but I did not find staff qualified enough to give me care
7. I did not know what to do
8. I did not think it was serious enough
9. I did not have enough time
10. Other-specify (802S. Specify (String 100))
88. Do not Know
99. No Response

→END OF INTERVIEW

803. Where did you seek advice or treatment? Please list all sources in the order that you got them.

- PROBE: Anywhere else?
- PROBE TO IDENTIFY THE SOURCE
- If primary health center, SPECIFY which one

1. At my house | 2. National hospital | 3. Regional hospital | 4. Community referral hospital (Bankass)

5. Primary health center-specify (805S. Specify (String 100)) | 6. Private Clinic / Hospital | 7. Pharmacy | 8. At CHW | 9. Home of CHW

10. At a traditional healer | 11. At home, a travelling seller | 12. Shop | 13. Other-specify (803S. Specify (String 100))

88. Do not Know | 99. No Response

804. Did someone evaluate you before you went to the health center? If so, who was it?

0. No →GO TO 808 | 1. Myself →GO TO 808 | 2. Husband →GO TO 808 | 3. Other women →GO TO 808

4. Other close relatives →GO TO 808 | 5. Friends / neighbors →GO TO 808 | 6. CHW →GO TO 805 | 7. Doctor →GO TO 808

8. Nurse →GO TO 808 | 9. Midwife →GO TO 808 | 10. Matron →GO TO 808 | 11. Traditional healer →GO TO 808

12. Drug seller →GO TO 808 | 88. Do not Know →GO TO 808 | 99. No Response →GO TO 808

805. Where was the evaluation conducted?

1. At home ↓ | 2. CHW Site →GO TO 807 | 3. Other-specify (805S. Specify (String 100)) ↓ | 8. Do not Know ↓ | 9. No Response ↓

806. How much time did it seem to take the CHW to arrive at your house?

1. Very long | 2. Long | 3. Normal | 4. Short | 5. Very short | 88. Do not Know | 99. No Response

807. How long did the evaluation last?

1. Less than an hour (less than the time between fitri and saafo) | 2. 1 hour (time between fitri and saafo)

3. 2 hours (time between salifana and star) | 4. ½ day | 5. 1 day | 6. 2 or more days | 88. Do not Know | 99. No Response

807CKPT.

1. If 803=2-7 OR 10-13 ↓	2. If 803=1, 8, 9, 88 OR 99 →GO TO 812

808. How much time did it seem to take to get to the health center?
  - If you went to several health centers, this is the last facility you attended.

1. Very long	2. Long	3. Normal	4. Short	5. Very short	88. Do not Know	99. No Response

809. At the health center, how long did you wait for care?

1. Less than an hour (less than the time between fitri and saafo)	2. 1 hour (time between fitri and saafo)

3. 2 hours (time between salifana and star)	4. ½ day	5. 1 day	6. 2 or more days	88. Do not Know	99. No Response

810. How did the waiting time seem to you?

1. Very long	2. Long	3. Normal	4. Short	5. Very short	88. Do not Know	99. No Response

811. How long did the medical consultation take?

1. Less than an hour (less than the time between fitri and saafo)	2. 1 hour (time between fitri and saafo)

3. 2 hours (time between salifana and star)	4. ½ day	5. 1 day	6. 2 or more days	88. Do not Know	99. No Response

812. Did you take time off from work to go to the clinic? That is to say, the work you do that generates income. If so, how many days?

- If no leave taken, ENTER 0
- If unknown, ENTER 88
- If no response, ENTER 99

0-99

813. While seeking care for your child, which of the following expenses did you obtain?

1. Medication / treatment

1. Yes	2. No	8. Do not Know	9. No Response

2. Laboratory / diagnostic tests

1. Yes	2. No	8. Do not Know	9. No Response

3. Other health expenses

1. Yes	2. No	8. Do not Know	9. No Response

4. Food / Accommodation

1. Yes	2. No	8. Do not Know	9. No Response

5. Other expenses

1. Yes	2. No	8. Do not Know	9. No Response

813CKPT.

1. If ALL 813==1 ↓	2. If ANY 813==2, 8, or 9	→END OF INTERVIEW

814. If Yes: How much did you pay for all these things?

- ENTER value in FCFA
- If unknown, ENTER 8888
- If no response, ENTER 9999

0-9999

END OF INTERVIEW

Before submitting the survey, return the census and ensure that the husband of the woman surveyed was identified if it is a usual resident of the household.

DEMOGRAPHICS SURVEY – YEAR ONE

Bankass

HOUSEHOLD CENSUS:

(1) LIST OF MEMBERS

C1. Date

- ENTER DD/MM/YYYY, with or without slashes

DATE

C1A. Interviewer ID

INTERVIEWER ID

C2. Language of Interview

1. Bamanankan	2. Tingu	3. Tomoso	4. Peulh	5. French	6. Other-specify (C2S. Specify (String 100))

C3. Health Area

AREANAME (C3S. Specify (String 100))

C4. Village Name

VILLAGENAME (C4S. Specify (String 100))

C5. Hamlet Name

HAMLETNAME (C5S. Specify (String 100))

C6A. Concession No.

- ENTER X/X/X/X, 4 digits

CONCESSIONNO

<u>HOUSEHOLD CENSUS</u>
List each household, starting with the head of the concession's household.

C6B. Household No.

- ENTER X/X, 2 digits

HOUSEHOLDNO

(1) LIST OF MEMBERS
<u>Household Members Loop (Max = 18)</u>
List all the permanent members of the household, starting with the head of the household. Circle among the options or mark the answer.

C7. Record Member ID

- Chief = 000
- Woman=100, 200
- Enfant = 101, 102, 201
- Another Member = 001, 002

MEMBERID

C8A. First Name

- Start with the head of the household.

FIRSTNAME (C8AS. Specify (String 100))

- Record a phone number for each household member. If no one in the household has a telephone, give the number of a neighbor or a close acquaintance.
- ENTER XXX/XXX/XXX with or without slashes

PHONE NUMBER

C8B. Family Name

FAMILYNAME (C8BS. Specify (String 100))

C9. Was the individual given a number in the baseline survey?

1. Yes	2. No

C10. Status

- Is [NAME] physically present, absent, or deceased in the village during the survey period?
- ENTER [1] to continue

1. Continue

C10A. Enter the status of the household member.

1. Present →GO TO C11 | 2. Absent | 3. Deceased →GO TO D1 | 4. Not in this Concession →GO TO C11 | 5. New Marriage →GO TO C11

↓

C10B. Why?

1. Permanent Migration | 2. Temporary Displacement | 3. Other-specify (C10BS. Specify (String 100))

C11CKPT. DOB

1. If C9==1 | 2. If C9==2 →GO TO C11A

↓

C11A. Is this date of birth correct?

1. Yes →GO TO C13 | 2. No →GO TO C11A

C11B. What is [NAME]'s date of birth?

C11A_Jour: Enter Day

- If unknown, enter "88"
- If no response, enter "99"

01-31, 88, 99

C11A_Mois: Enter Month

- If unknown, enter "88"
- If no response, enter "99"

01-12, 88, 99

C11A_Année: Enter Year

- If unknown, enter "8888"
- If no response, enter "9999"

1900-2100, 8888, 9999

C12. What is the source of the date of birth?

1. Memory	2. Official Document

C13. (Not asked of the head of household) During which season was this person born?

1. Heat (March, April, May)	2. Sowing of millet / early rain (June)	3. Sowing peanut and maize / first crop (July)	4. Chocho / heavy rain / second crop (August)
5. Corn harvest (September)	6. Harvest of millet and peanuts (October, November)	7. The Cold (December, January, February)	
88. Do not Know	99. No Response		

C14. Age

- Enter the age in years if 1 year of age or older. Enter the age in number of months and days if less than 1 year of age.
- Mark 00 if the child is less than one year, 00 months if the child is less than one month old.
- How old is [NAME]?

Years-Specify (0-100)	Months-Specify (0-12)	Days-Specify (0-31)

C15CKPT. Relationship to Head of Household

1. If C9==1 →GO TO C15B	2. If C9==2 ↓

C15A. Choose the most accurate response option:

1. Head of household	2. Spouse	3. Daughter / son	4. Father / mother	5. Brother / sister	6. Son-in-law / daughter-in-law	
7. Grandchild	8. Grandparent	9. Cousin	10. Nephew / niece	11. Uncle / aunt	12. Visitor	13. Foster child / adopted
14. Other-specify (C15AS. Specify (String 100))						

C15B. Is the relationship to the head of the household correct?

1. Yes →GO TO C16	2. No →GO TO C15A

C16. Gender

1. Male | 2. Female

C17. Are there any members in your household, such as children, adopted children, recently married women, or others who have been added to your household since the last survey, that is to say, since the period of harvest at the time of the cold?

1. Yes, there are some →CENSUS THE NEW PERSON (GO TO C7-C16) | 2. No, there are none ↓

C18. Select the person who answered the household census.

End of Household Members Loop

Questions for people who said "migration" in the status question

M1. Where did [NAME] go?

1. Elsewhere in Bankass district-specify (M1S. Specify (String 100)) | 2. Mopti urban area out of Bankass-specify (M1S. Specify (String 100))

3. Mopti rural area out of Bankass-specify (M1S. Specify (String 100)) | 4. Bamako | 5. Elsewhere in Mali-specify (M1S. Specify (String 100))

6. Other country-specify (M1S. Specify (String 100))

M2. When did [NAME] go?

- Survey of the month looking for the seasons (rain, harvest, hot, etc.)

Month-specify (M2S. Specify (String 100)) | Year-specify (M2S. Specify (String 100))

M3. Why did [NAME] leave?

1. Looking for work | 2. To work (work already obtained) | 3. Marriage | 4. Divorce | 5. Adoption | 6. Education

7. Family Relocation | 8. Other-specify (M3S. Specify (String 100))

M4. Is [NAME] sending money to the members of the household since he left?

1. Yes | 2. No

→GO TO C11 (DDN)

↓

M4A. How often?

1. More than once a month	2. Once a month	3. Once every two months	4. Once a quarter	5. Twice a year	6. Once a year

→GO TO C11 (DOB)

M4B. If yes how much?

- Do not know = 88
- Without answer = 99

Value-specify (M4BS. Specify (String 100))

After the list of members, ask the following questions for each household:

K1A. From the last survey to now, that is, since the time of harvest at the time of cold, did anyone in your household give birth?

- Start with the most recent childbirth.

1. Yes | 2. No →GO TO K2A

↓

K1B. Is the baby still alive, or did he die after birth?

1. Alive → View the list of registered members (to select the child) with a field to add a new one (C7 to C16)

2. Deceased → Fill C7 to C10, and after the mortality section for this child

K1B_ID. Census ID for baby

BABYID

K1C. From the last survey to now, that is to say, since the time of harvest at the time of cold, did anyone else in your household give birth?

1. Yes →GO TO K1B & K1C | 2. No →GO TO K2A

K2A. From the last survey to now, that is, since the time of harvest at the time of cold, in your household has a child been adopted by anyone?

- Start with the most recent adoption.

1. Yes → View the list of registered members (to select the child) with a field to add a new one (C7 to C16)

2. No → GO TO K3A

K2B_ID. Census ID for adopted child

ADOPTED_ID

K3A. From the last survey to now, that is to say, since the period of harvest at the time of cold, did anyone in your household get married?

- These are women who are married elsewhere or men who have had a wife in your household. Start with the most recent marriage.

1. Yes ↓ 2. No →GO TO K4A

K3B. Is this a new person who has arrived in your household or a person who has gone elsewhere?

1. New Person → View the list of registered members (to select the child) with a field to add a new one (C7 to C16)

2. Gone Elsewhere → GO TO K3D

K3C. Was this person censused in the study last year (does she have a Muso health card)?

1. Yes → NOTE THE WOMAN'S STUDY NUMBER (SEE HER ENROLLMENT CARD) 2. No ↓

K3D. Give the name and family name of the person concerned, and specify the destination and date of departure.

K3D_prenom: First Name

FIRSTNAME (K3D_prenomS. Specify (String 100))

K3D_ nom: Family name

FAMILYNAME (K3D_nomS. Specify (String 100))

Date of departure:
K3D_mois: Month

Month-specify (K3D_moisS. Specify (String 100))

K3D_annee: Year

Year-specify (K3D_anneeS. Specify (String 100))

Destination: Where did [NAME] go?

- MARK THE PERSON'S ID (THE ID ON THE HOUSEHOLD CARD)

1. Elsewhere in Bankass district-specify (K3D_1S. Specify (String 100))
2. Mopti urban area out of Bankass-specify (K3D_2S. Specify (String 100))
3. Mopti rural area out of Bankass-specify (K3D_3S. Specify (String 100))
4. Bamako
5. Elsewhere in Mali-specify (K3D_5S. Specify (String 100))
6. Other country-specify (K3D_6S. Specify (String 100))

K3E. Did this person come / go with a child?

1. Yes → Display the list of registered members (to select the child) with a field to add a new one (C7 to C16)
2. No ↓

K3F. Did this person come / go with another child?

1. Yes → Display the list of registered members (to select the child) with a field to add a new one (C7 to C16)
2. No ↓

K3G. From the last survey to now, that is, since the time of harvest at the time of cold, did anyone else in your household get married?

1. Yes →RETURN TO K3B
2. No ↓

K4A. From the last survey to now, that is, since the time of harvest at the time of cold, are there other people who have been added to your household and who have not been censused?

- It is also the people added to the household who died later.

1. Yes ↓
2. No →GO TO K5

K4B. Was this person enlisted in the study last year (does she have a Muso health card)?

1. Yes
2. No

→ NOTE THE WOMAN'S STUDY NUMBER (SEE HER ENROLLMENT CARD)

↓

K4C. Is the person alive or dead?

1. Alive → View the list of registered members (to select the child) with a field to add a new one (C7 to C16)

2. Deceased → Fill C7 to C10, and after the mortality section for this child

K4C_ID. CensusID of the new household member

NEWMEMBER_ID

K4D. From the last survey to now, that is, since the time of the harvest at the time of cold, are there other people who have joined your household and who have not been mentioned?

1. Yes →REPEAT C7 to C16 2. No

↓

K5. The interviewer must read the list of all members of the household to be sure nothing has been omitted.

- Is there a member who has been forgotten to count in your household?

1. Yes →FILL C7-C16 2. No

↓

K5_ID. CensusID for other new household member

NEWMEMBER_ID

HOUSEHOLD CENSUS:
(2) MORTALITY

D1. Since the last survey, that is, since the time of the harvest at the time of cold, has anyone in your household died?

- Note that this includes new births after the last survey who died before today's survey.

1. Yes ↓	2. No → GO TO 116	8. Do not Know → GO TO 116	9. No Response → GO TO 116

Mortality Loop (Max = 20)
Beginning with the most recent death, please, tell me:

D2. The first name and family name of the person?

- ENTER FIRST NAME

FIRSTNAME (String 100)

- ENTER FAMILY NAME

FAMILYNAME (String 100)

D3. Relationship to head of household?

1. Head of household	2. Spouse	3. Daughter / son	4. Father / mother	5. Brother / sister	6. Son-in-law / daughter-in-law
7. Grandchild	8. Grandparent	9. Cousin	10. Nephew / niece	11. Uncle / aunt	12. Visitor
13. Foster child / adopted					
14. Other-specify (D3S. Specify (String 100))					

D4. What is the date of death of [NAME]?

- ENTER BOTH THE MONTH AND YEAR OF DEATH
- ENTER the month of death in 2 digits, 1 to 12.
- If unknown month, ENTER 88
- If no response, ENTER 99

00-12, 88, 99

- ENTER the year of death in 4 digits, like “1960”
- If unknown year, ENTER 8888.
- If no response, ENTER 9999.

1900-2100, 8888, 9999

D4A. During which season did [NAME] die?

1. Heat (March, April, May)	2. Sowing of millet / early rain (June)	3. Sowing peanut and maize / first crop (July)	4. Chocho / heavy rain / second crop (August)
5. Corn harvest (September)	6. Harvest of millet and peanuts (October, November)	7. The Cold (December, January, February)	

D4B. VERIFICATION OF THE DATE OF DEATH: If the date of death is before December 1, 2016, check that the date is correct.

- I want to make sure that I have entered the correct date of death for [NAME].
- What is the date of death of [NAME]?
- ENTER the month of death in 2 digits, 1 to 12.
- If unknown month, ENTER 88
- If no response, ENTER 99

01-12, 88, 99

- ENTER the year of death in 4 digits, like "1960"
- If unknown year, ENTER 8888.
- If no response, ENTER 9999.

1900-2100, 8888, 9999

D5. What is the date of birth of [NAME]?

- ENTER DAY
- If unknown day, ENTER "88".
- If no response, ENTER "99".

01-31, 88, 99

- ENTER MONTH
- If unknown month, ENTER "88".
- If no response, ENTER "99".

01-12, 88, 99

- ENTER YEAR
- If unknown year, ENTER "88".
- If no response, ENTER "99".

1900-2100, 8888, 9999

D6. How old was (NAME) when he died?

- ALL FIELDS ARE MANDATORY
- ENTER years of age
- MARK 00 years if the child is under one year
- If unknown number of years, ENTER 88
- If no response, ENTER 99

01-99

- ENTER months of age
- MARK 00 months if the child is less than one month old.
- If unknown number of months, ENTER 88
- If no response, ENTER 99

01-12, 88, 99

- ENTER days of age
- If unknown number of days, ENTER 88.
- If no response for age, ENTER 99.

01-31, 88, 99

D7. CHKPT: WHETHER RECORDED DOB MATCHES AGE

1. RECORDED DATE OF DEATH DOES NOT MATCH AGE (D5– D6 ≠ 0) ↓	2. RECORDED DATE OF DEATH DOES MATCH AGE (D5 – D6= 0)	→ GO TO D8

If the difference between age and DDN is not equal to 0, probe:

D7B. I recorded that (NAME) was X months / years, and that he / she died in (MONTH / YEAR). I want to make sure that I recorded the exact age of (NAME) at the time of death. How old was (NAME) when he died?

- ENTER years
- If unknown years, ENTER 88
- If no response, enter 99

01-99

- ENTER months
- If unknown months, ENTER 88
- If no response, ENTER 99

01-12, 88, 99

- ENTER days
- If unknown days, ENTER 88
- If no response, ENTER 99

01-31, 88, 99

D7C. I want to make sure that I have entered the date of death of (NAME). What is the date of death of (NAME)?

- ENTER month
- If unknown month, ENTER 88
- If no response, ENTER 99

01-12, 88, 99

- ENTER year
- If unknown year, ENTER 8888
- If no response, ENTER 9999

1900-2100, 8888, 9999

D7D. I want to make sure that I have entered the date of birth of (NAME). What is the date of birth of (NAME)?

- ENTER day

- If unknown day, ENTER 88
- If no response, ENTER 99

01-31, 88, 99

- ENTER month
- If unknown month, ENTER 88
- If no response, ENTER 99

01-12, 88, 99

- ENTER year
- If unknown year, ENTER 8888
- If no response, ENTER 9999

1900-2100, 8888, 9999

D8CKPT. VERIFICATION: IS IT A CHILD?

1. RECORDED AGE UNDER 18 (D7B<18)	2. RECORDED AGE OVER 18 (D7B>18)	→ GO TO D12
↓		

D8. If child, is the child's biological mother currently living in the household?

1. Yes ↓	2. No → GO TO D10	8. Do not Know → GO TO D10	9. No Response → GO TO D10

D9. Mark the last three digits of the mother's ID.

000-999

D10. Is the biological father of the child currently living in the household?

1. Yes ↓ | 2. No → GO TO D12 | 8. Do not Know → GO TO D12 | 9. No Response → GO TO D12

D11. Mark the last three digits of the father's ID.

000-999

D12. Was (NAME) male or female?

1. Male → GO TO D16 | 2. Female ↓

D13. Was she pregnant when she died?

1. Yes | 2. No | 8. Do not Know | 9. No Response

D14. Did she die during childbirth?

1. Yes → GO TO D16 | 2. No ↓ | 8. Do not Know ↓ | 9. No Response ↓

D15. Did she die within two months of giving birth or ending the pregnancy (abortion)?

1. Yes | 2. No | 8. Do not Know | 9. No Response

D16. From the last survey to now, that is, since the time of the harvest at the time of cold, did anyone who lived in your household die? Note that this includes new births after the last survey who died before today's survey.

1. Yes → GO TO D2 | 2. No →GO TO 116 | 8. Do not Know →GO TO 116 | 9. No Response →GO TO 116

<u>End of Mortality Loop (Max = 20)</u>

HOUSEHOLD CENSUS:
(3) HOUSEHOLD CHARACTERISTICS

Household Number (2 Digits)

00-99

115A. OBSERVE MAIN MATERIAL OF THE ROOF OF THE DWELLING.

- What is the main material of the roof of the dwelling?
- IF POSSIBLE, NOTE YOUR OBSERVATION. IF NOT POSSIBLE TO OBSERVE, ASK THE RESPONDENT IF YOU CAN OBSERVE

11. No Roof	12. Thatch/Palm Leaf	13. Sod	21. Rustic Mat	22. Palm/Bamboo	23. Wood Planks	24. Cardboard
31. Metal	32. Wood	33. Calamine/Cement Fiber	34. Ceramic Tiles	35. Cement	36. Roofing Shingles	
96. Other-specify (115AS. Specify (String 100))						

115B. OBSERVE MAIN MATERIAL OF THE EXTERIOR WALLS OF THE DWELLING.

- What is the main material of the exterior walls of the dwelling?
- IF POSSIBLE, NOTE YOUR OBSERVATION. IF NOT POSSIBLE TO OBSERVE, ASK THE RESPONDENT IF YOU CAN OBSERVE

11. No Walls	12. Cane/Palm/Trunks	13. Dirt	21. Bamboo with Mud	22. Stone with Mud	23. Uncovered Adobe	24. Plywood
25. Cardboard	26. Reused Wood	31. Cement	32. Stone with Lime/Cement	33. Bricks	34. Cement Blocks	35. Covered Adobe
36. Wood Planks/Shingles	96. Other-specify (115BS. Specify (String 100))					

116A. What is the main source of drinking water for members of your household?

- NOTE: Surface water could be river, dam, lake, pond, stream, canal, irrigation channel

11. Piped into dwelling	12. Piped into yard/plot	13. Piped to neighbor	14. Public tap / standpipe	21. Tube well or borehole	
31. Protected well	32. Unprotected well	41. Protected spring	42. Unprotected spring	51. Rainwater	61. Tanker truck
71. Cart with small tank	81. Surface water	96. Other-specify (116AS. Specify (String 100))	88. Do not know	99. No response	

116B. Do you do anything to the water to make it safer to drink?

1. Yes ↓ | 2. No → GO TO 117A | 8. Do not Know → GO TO 117A | 9. No Response → GO TO 117A

116C. What do you usually do to make the water safer to drink?

- ENTER all that apply
- PROBE: Anything else?

1. Boil | 2. Add bleach/chlorine | 3. Strain through a cloth | 4. Use water filter (ceramic/sand/composite/etc)
5. Solar disinfection | 6. Let it stand and settle | 7. Other-specify (116CS. Specify (String 100)) | 88. Do not Know
99. No Response

116D. How often do you treat water to drink?

1. Always | 2. Often | 3. Sometimes | 4. Rarely | 88. Do not Know | 99. No Response

117A. What kind of toilet facility do members of your household usually use?

- If not possible to determine, ask permission to observe the facility.

11. Flush to piped sewer system | 12. Flush to septic tank | 13. Flush to pit latrine | 14. Flush to somewhere else
15. Flush, don't know where | 21. Ventilated improved pit latrine | 22. Pit latrine with slab | 23. Pit latrine without slab/open pit
31. Composting toilet | 41. Bucket toilet | 51. Hanging toilet | 61. No facility/bush/field →GO TO 118
96. Other-specify (117AS. Specify (String 100)) | 88. Do not Know | 99. No response

117B. Where is this toilet facility located?

1. In own dwelling | 2. In own yard/plot | 3. Elsewhere

117C. Do you share this toilet facility with other households?

1. Yes | 2. No | 8. Do not Know | 99. No response

118. Does your household have:

1. Electricity?

1. Yes | 2. No

2. A radio?

1. Yes | 2. No

3. A television ?

1. Yes | 2. No

4. A mobile phone ?

1. Yes | 2. No

5. A refrigerator ?

1. Yes | 2. No

6. An antenna TV5?

1. Yes | 2. No

7. Subscription to Cable?

1. Yes | 2. No

8. An improved home?

1. Yes | 2. No

9. A video / CD / DVD player?

1. Yes | 2. No

119. What type of fuel does your household mainly use for cooking?

01. Electricity | 02. LPG | 03. Natural Gas | 04. Biogas | 05. Kerosene | 06. Coal, lignite | 07. Charcoal | 08. Wood

09. Straw / shrubs / grass | 10. Agricultural crop | 11. Animal Dung | 95. No food cooked in household | 96. Other-specify (119S. Specify (String 100))

88. Do not Know | 99. No Response

120. OBSEVE MAIN MATERIAL OF THE FLOOR OF THE DWELLING. RECORD OBSERVATION.

- If natural floor, SPECIFY:

11. Earth/Sand | 12. Dung

- If rudimentary floor, SPECIFY:

21. Wooden planks | 22. Palms / bamboo

- If finished floor, SPECIFY:

31. Parquet or polished wood | 32. Vinyl or asphalt strips | 33. Ceramic tiles | 34. Cement | 35. Carpet

96. Other-specify (120S. Specify (String 100))

121. Does any member of this household own:

1. A watch ?

1. Yes | 2. No

2. A plow?

1. Yes | 2. No

3. A bicycle ?

1. Yes | 2. No

4. A motorcycle or a scooter?

1. Yes | 2. No

5. An animal-drawn cart?

1. Yes | 2. No

6. A tractor ?

1. Yes | 2. No

7. A car or truck?

1. Yes | 2. No

122. How many of the following animals does this household own?

- NONE = 00
- 95 OR MORE = 95

- DO NOT KNOW = 888
- NO RESPONSE = 999

1. Dairy cows or bulls?

00-94, 95, 888, 999

2. Livestock?

00-94, 95, 888, 999

3. Horses, donkeys or mules?

00-94, 95, 888, 999

4. Goats?

00-94, 95, 888, 999

5. Sheep?

00-94, 95, 888, 999

6. Chickens?

00-94, 95, 888, 999

123. In this household, how many rooms / places are used for sleeping?

Rooms (ENTER 00-99)	Places (ENTER 00-99)

124. In the last 30 days, was there ever no food to eat of any kind in your house because of a lack of resources to get food?

1. Yes ↓ | 2. No → GO TO 126 | 8. Do not Know → GO TO 126 | 9. No Response → GO TO 126

125. How often did this happen in the last 30 days?

1. Rarely (1-2 times)	2. A few times (3-10 times)	3. Often (more than 10 times)

126. In the past 30 days, did you or any household member go to sleep at night hungry because there was not enough food?

1. Yes ↓ | 2. No → GO TO 128 | 8. Do not Know → GO TO 128 | 9. No Response → GO TO 128

127. How often did this happen in the last 30 days?

1. Rarely (1-2 times)	2. A few times (3-10 times)	3. Often (more than 10 times)

128. In the past 30 days, did you or any household member go a whole day and night without eating anything because there was not enough food?

1. Yes ↓ | 2. No → GO TO 130 | 8. Do not Know → GO TO 130 | 9. No Response → GO TO 130

129. How often did this happen in the last 30 days?

1. Rarely (1-2 times)	2. A few times (3-10 times)	3. Often (more than 10 times)

WOMEN'S SURVEY SCREENING

IDENTIFICATION OF THE WOMAN INTERVIEWED

C1. Date

- ENTER DD/MM/YYYY, with or without slashes

DATE

C1A. Interviewer ID

INTERVIEWER ID

C2. Language of Interview

1. Bamanankan	2. Tingu	3. Tomoso	4. Peulh	5. French	6. Other-specify (C2S. Specify (String 100))

C3. Health Area

AREANAME (C3S. Specify (String 100))

C4. Village Name

VILLAGENAME (C4S. Specify (String 100))

C5. Hamlet Name (name or none)

HAMLETNAME (C5S. Specify (String 100))

C6A. Concession No.

- ENTER XXX/XXX/XXX/XXX, with or without slashes

CONCESSIONNO

- ENTER the first name of the head of the household

FIRSTNAME (C6AS. Specify (String 100))

- ENTER the family name of the head of the household

FAMILYNAME (C6AS. Specify (String 100))

C[illegible]

FIRSTNAME (C8AS. Specify (String 100))

C8B. Family Name of the Woman

FAMILYNAME (C8BS. Specify (String 100))

C7. Record Woman's ID

- ENTER XXX

MEMBERID

INFORMATION ON THE SCREENING OF THE WOMAN INTERVIEWED

E0. Has the woman previously participated in this survey, or is she a new participant?

1. Former Participant →GO TO 103 | 2. New Participant ↓

E1. Is the woman aged 15-49 years?

1. Yes | 2. No

E2. Permanent resident?

1. Yes | 2. No

E3. Is the woman expected to remain in the study area during the next three years?

1. Yes | 2. No

E4. Is the woman willing to participate and complete the study program?

1. Yes | 2. No

E5CKPT. ELIG CHECKPOINT: WHETHER PARTICIPANT IS ELIGIBLE OR NOT

- If no to one of questions 1, 2, 3 and 4 the woman is not eligible for study. So do not continue the interview
- If yes to all questions 1, 2, 3 and 4 then the woman is eligible

1. Yes, Eligible ↓ | 2. Not Eligible →GO TO E7

E5. Is the woman eligible?

1. Yes | 2. No

E6. If eligible, is she included in the study?

1. Yes | 2. No

→GO TO 101CKPT

E7. If not included, give reasons:

a. Declined consent

1. Yes | 2. No

b. Need to consult family

1. Yes | 2. No

c. Not convinced

1. Yes | 2. No

d. Family refused consent

1. Yes | 2. No

→END OF INTERVIEW

Section 1: Demographic Details

101CKPT.

1. If C9==1 OR E0==1	→GO TO 103	2. If C9==2 OR E0==2 ↓

101. Have you ever attended school (madrasah or French)?

1. Yes ↓	2. No → GO TO 103	8. Do not Know → GO TO 103	9. No response → GO TO 103

102A. What is the highest level of school you have completed:

- Primary 1 (1 cycle), primary 2 (2nd cycle), secondary (high school, technical) or higher?

1. Primary 1 (1st cycle)	2. Primary 2 (2nd cycle)	3. Secondary (high school / technical)	4. More than secondary
8. Do not Know	9. No Response		

102B. How many years / classes did you complete at this level?

- IF LESS THAN A YEAR HAS BEEN COMPLETED AT THIS LEVEL, SPECIFY
- If unknown, enter "8"
- If no response, enter "9"

CLASS / YEAR	8. Do not Know	9. No Response

103. Now I would like you to read this sentence to me.

- SHOW CARD TO RESPONDENT.
- IF THE RESPONDENT CANNOT READ WHOLE SENTENCE, PROBE: Can you read any part of the sentence to me?

1. Cannot read at all	2. Able to read only part of the sentence	3. Able to read whole sentence
4. No card with required language-specify (103S. Specify (String 100))	5. Blind / visually impaired	

104. How many people does your household currently have?

- This is the household and not the concession. Count those who usually live in your household
- If the number of people in the household is unknown, ENTER 88.
- If there is no response, ENTER 99.

0-99

105. What religion do you practice?

1. Muslim	2. Catholic	3. Methodist	4. Evangelical	5. Other Christian religion	6. Animist
7. Other religions-specify (105S. Specify (String 100))	8. Without Religion	88. Do not Know	99. No Response		

106CKPT.

1. If C9==1 OR E0==1	→GO TO 107	2. If C9==2 OR E0==2 ↓

106. What is your ethnicity?

1. Dogon	2. Peulh	3. Bambara	4. Mossi	5. Malinke	6. Sarakole / soninke / marka	7. Sonraï	8. Tamachek / Bella
9. Sénoufo / Minianka	10. Bobo	11. Boso	12. Other Malian ethnic-specify (106S. Specify (String 100))	13. CEDEAO country			
14. Other African countries-specify (106S. Specify (String 100))	15. Other non-African countries-specify (106S. Specify (String 100))						
88. Do not know	99. No response						

107. Are you currently married or living together with a man as if married?

1. Yes, currently married → GO TO 110A	2. Yes, living with a man →GO TO 110A	3. No, not in union ↓	8. Do not Know ↓	9. No Response ↓

108. Have you ever been married or lived together with a man as if married?

1. Yes, formerly married ↓	2. Yes, lived with a man ↓	3. No →GO TO 130

109. What is your marital status now: are you widowed, divorced or separated?

1. Widowed	→GO TO 130	2. Divorced	→GO TO 130	3. Separated	→GO TO 130

110A. How old were you when you started living with your husband?

- If unknown, ENTER "88"
- If no response, ENTER "99"

Age in Years (0-99)

110B. Is your (husband/partner) living with you now or is he staying elsewhere?

1. Living with her →GO TO 111	2. Living with her sometimes ↓	3. Staying elsewhere ↓	8. Do not Know ↓	9. No Response ↓

110B. HUSBAND/PARTNER [YES, MARRIED/YES, LIVING WITH]
During the month, how much time does your [husband / partner] live with you in this household?

1. 25%	2. 50%	3. 75%

110C. Where is his other home?

1. In this village	2. In another village-specify (110CS. Specify (String 100))

111. Does your (husband / partner) have other wives or does he live with other women as if married?

1. Yes ↓	2. No →GO TO 114A	8. Do not Know →GO TO 114A	9. No Response →GO TO 114A

112. Including yourself, in total, how many wives or live-in partners does he have?

- ENTER total number of wives and live-in partners
- If unknown, ENTER "88"
- If no response, ENTER "99"

01-99

113. Are you the first, second, ... wife?

- Rank:
- If rank unknown, ENTER "88"
- If no response, ENTER "99"

00-99

114A. What i[illegible]st level of school your (husband/partner) has completed:

- Primary 1 (1 cycle), primary 2 (2nd cycle), secondary (high school, technical) or higher?

1. Primary 1 (1st cycle)	→GO TO 114B	2. Primary 2 (2nd cycle)	→GO TO 114B		
3. Secondary (high school / technical)	→GO TO 114B	4. More than secondary	→GO TO 114B	5. Did not go to school	→GO TO 115
8. Do not Know	→GO TO 115	9. No Response	→GO TO 115		

114B. How many years / classes did your husband/partner complete at this level

- IF LESS THAN A YEAR HAS BEEN COMPLETED AT THIS LEVEL, SPECIFY
- If unknown, enter “88”
- If no response, enter “99”

CLASS / YEAR	88. Do not Know	99. No Response

115CKPT.

1. If C9==1 OR E0==1	→GO TO 130	2. If C9==2 OR E0==2 ↓

115. What is your (husband’s/partner’s) occupation? That is, what kind of work does he mainly do?

1. Cultivator	2. Breeder	3. Trader	4. Butcher	5. Bricklayer	6. Official	7. Contractor	8. Other-specify (115S. Specify (String 100))
88. Do not Know	99. No Response						

130. As you know, some women take up jobs for which they are paid in cash or kind. Others sell things, have a small business or work on the family farm or in the family business. During the past month, did you do any of these things or any other work for which you were paid in cash or in kind?

1. Yes	2. No	8. Do not Know	9. No Response

131. What is your occupation? That is, what kind of work do you mainly do?

1. Housewife	2. Small business	3. Trader	4. Planter	5. Official	6. Contractor	7. Other-specify (131S. Specify (String 100))
88. Do not Know	99. No Response					

132. Do you do this work for a member of your family, for someone else, or are you self-employed?

1. For family member	2. For someone else	3. Self-employed	8. Do not Know	9. No Response

13[illegible]. Do you usually work throughout the year, or do you work seasonally, or only once in a while?

1. Throughout the year	2. Seasonally / part of the year	3. Once in a while	4. No	8. Do not Know	9. No Response

134A. Are you paid in cash or kind for this work or are you not paid at all?

1. Cash only	→GO TO 134B	2. Cash and kind	→GO TO 134B	3. In kind only	→GO TO 134C	4. Not paid	→GO TO 136
8. Do not Know	→GO TO 135	9. No Response	→GO TO 135				

134B. How much money do you get for this job?

- ENTER the value in FCFA:
  - If value unknown, ENTER "8888"
  - If no response, ENTER "9999"

  0000-9999

- SPECIFY how often:

1. Daily	2. Weekly	3. Monthly	4. Other-specify (134BS. Specify (String 100))	8. Do not Know	9. No Response

134C. How much, in your estimation, is the cash value of this payment in kind?

- ENTER the value in FCFA:
- If value unknown, ENTER "8888"
- If no response, ENTER "9999"

  0000-9999

- SPECIFY how often:

1. Daily	2. Weekly	3. Monthly	4. Other-specify (134CS. Specify (String 100))	8. Do not Know	9. No Response

135. Who usually decides how the money you earn will be used?

1. Respondent	2. Husband/Partner	3. Concession leader	4. Respondent and husband/partner jointly	5. Respondent and concession leader jointly
6. Husband and concession leader jointly	7. Other-specify (135S. Specify (String 100))	88. Do not Know	99. No Response	

136. HUSBAND/PARTNER [YES, MARRIED/YES, LIVING WITH]

Who usually decides how your (husband's/partner's) earnings will be used?

1. Respondent	2. Husband/Partner	3. Concession leader	4. Respondent and husband/partner jointly	5. Respondent and concession leader jointly
6. Husband and concession leader jointly	7. Other-specify (136S. Specify (String 100))	88. Do not Know	99. No Response	

137. Who usually makes decisions about health care for yourself?

1. Respondent	2. Husband/Partner	3. Concession leader	4. Respondent and husband/partner jointly	5. Respondent and concession leader jointly
6. Husband and concession leader jointly	7. Other-specify (137S. Specify (String 100))	88. Do not Know	99. No Response	

138. Who usually makes decisions about making major household purchases?

1. Respondent	2. Husband/Partner	3. Concession leader	4. Respondent and husband/partner jointly	5. Respondent and concession leader jointly
6. Husband and concession leader jointly	7. Other-specify (138S. Specify (String 100))	88. Do not Know	99. No Response	

139. Who usually makes decisions about visits to your family or relatives?

1. Respondent	2. Husband/Partner	3. Concession leader	4. Respondent and husband/partner jointly	5. Respondent and concession leader jointly
6. Husband and concession leader jointly	7. Other-specify (139S. Specify (String 100))	88. Do not Know	99. No Response	

140. Sometimes a husband is upset or irritated by the things his wife does. In your opinion, is a husband justified in hitting or beating his wife in the following situations:

[A] If she goes out without telling him?

1. Yes	2. No	8. Do not Know	9. No Response

[B] If she neglects the children?

1. Yes	2. No	8. Do not Know	9. No Response

[C] If she argues with him?

1. Yes	2. No	8. Do not Know	9. No Response

[D] If she refuses to have sex with him?

1. Yes	2. No	8. Do not Know	9. No Response

[E] If she burns the food?

1. Yes	2. No	8. Do not Know	9. No Response

[F] If she uses contraceptives without the husband's consent?

1. Yes	2. No	8. Do not Know	9. No Response

[G] If she argues with the husband / partner's parents?

1. Yes	2. No	8. Do not Know	9. No Response

141ACKPT.

1. If C9==1 OR E0==1	→GO TO 141B	2. If C9==2 OR E0==2 ↓

141A. Have you ever:

- Been to the market?

1. Yes	2. No	8. Do not Know	9. No Response

- Been there alone?

1. Yes	2. No	8. Do not Know	9. No Response

- Been to the hospital/clinic/doctor?

1. Yes	2. No	8. Do not Know	9. No Response

- Gone there alone?

1. Yes	2. No	8. Do not Know	9. No Response

- Gone to the cinema?

1. Yes	2. No	8. Do not Know	9. No Response

- Gone there alone?

1. Yes	2. No	8. Do not Know	9. No Response

- Gone outside the village?

1. Yes	2. No	8. Do not Know	9. No Response

- Gone there alone?

1. Yes	2. No	8. Do not Know	9. No Response

141B. Now I am going to read you some statements. Please tell me whether you agree or disagree with each one.

1. It is important that sons have more education than daughters.

1. Yes	2. No	8. Do not Know	9. No Response

2. Girls should only be sent to school if they do not need to help at home.

1. Yes	2. No	8. Do not Know	9. No Response

3. The most important reason why sons should be more educated than girls is that they can better care for their parents when they are older.

1. Yes	2. No	8. Do not Know	9. No Response

4. If there is a limited amount of money to pay for tutoring, it will first be spent on sons.

1. Yes	2. No	8. Do not Know	9. No Response

5. A woman should take care of her own children and not worry about other people's business.

1. Yes	2. No	8. Do not Know	9. No Response

6. Women should leave politics to men.

1. Yes	2. No	8. Do not Know	9. No Response

7. A woman must have a husband or sons or other male parent to protect her.

1. Yes	2. No	8. Do not Know	9. No Response

8. The only thing a woman can really count on in her old age is her sons.

1. Yes	2. No	8. Do not Know	9. No Response

9. A good woman never questions her husband's opinions, even if she is not sure if she agrees with them.

1. Yes	2. No	8. Do not Know	9. No Response

10. When it comes to children's health, it's better to do what the father wants.

1. Yes	2. No	8. Do not Know	9. No Response

11. Girls should be able to work outside the home after having children if they want to.

1. Yes	2. No	8. Do not Know	9. No Response

12. Girls should have the same chance to work outside the home as boys.

1. Yes	2. No	8. Do not Know	9. No Response

13. Girls should be told that an important reason for not having children is to be able to work outside the home and make money.

1. Yes	2. No	8. Do not Know	9. No Response

14. I would like my daughter to be able to work outside the house so that she can provide for herself, as needed.

1. Yes	2. No	8. Do not Know	9. No Response

141C. Indicate the frequency with which you are in the following situations:

1. You were afraid for no reason.

1. Always	2. Sometimes	3. Rarely	4. Never	8. Do not Know	9. No Response

2. You felt restless.

1. Always	2. Sometimes	3. Rarely	4. Never	8. Do not Know	9. No Response

3. You lacked energy.

1. Always	2. Sometimes	3. Rarely	4. Never	8. Do not Know	9. No Response

4. You cried easily.

1. Always	2. Sometimes	3. Rarely	4. Never	8. Do not Know	9. No Response

5. You lost your appetite.

1. Always	2. Sometimes	3. Rarely	4. Never	8. Do not Know	9. No Response

6. You had trouble sleeping.

1. Always	2. Sometimes	3. Rarely	4. Never	8. Do not Know	9. No Response

7. You felt hopeless.

1. Always	2. Sometimes	3. Rarely	4. Never	8. Do not Know	9. No Response

8. You felt depressed.

1. Always	2. Sometimes	3. Rarely	4. Never	8. Do not Know	9. No Response

9. You felt lonely.

1. Always	2. Sometimes	3. Rarely	4. Never	8. Do not Know	9. No Response

10. You worried too much.

1. Always	2. Sometimes	3. Rarely	4. Never	8. Do not Know	9. No Response

11. You spoke to yourself.

1. Always	2. Sometimes	3. Rarely	4. Never	8. Do not Know	9. No Response

12. It was difficult to speak to others.

1. Always	2. Sometimes	3. Rarely	4. Never	8. Do not Know	9. No Response

13. You felt that your heart was broken or hurt.

1. Always	2. Sometimes	3. Rarely	4. Never	8. Do not Know	9. No Response

14. Your mind wandered or you felt distracted.

1. Always	2. Sometimes	3. Rarely	4. Never	8. Do not Know	9. No Response

15. You were easily angered.

1. Always	2. Sometimes	3. Rarely	4. Never	8. Do not Know	9. No Response

Section 1B: CHW Care

142. Do you know how to contact or find your Community Health Worker in case of need?

1. Yes	2. No	3. I have no CHW →GO TO 201	8. Do not Know	9. No Response

143. During the past month, have you met your community health worker for advice or care?

1. Yes ↓	2. No →GO TO 201	8. Do not Know →GO TO 201	9. No Response →GO TO 201

144. If so, where did you meet the CHW?

1. At your house	2. At the CHW site	3. Other-specify (144S. Specify (String 100))	8. Do not Know	9. No Response

145. How many times?

- ENTER number of times
    - If unknown number of times, ENTER "88"
    - If no response, ENTER "99"

00-99

Section 2: Reproductive health
2A. Family planning

201ACKPT.

1. If C9==1 OR E0==1 AND Method==0	→GO TO 201D	2. If C9==2 OR E0==2 ↓

201A. Are you or your partner currently doing something or using any method to delay or avoid getting pregnant?

1. Yes →GO TO 202 | 2. No →GO TO 218B | 8. Do not Know →GO TO 218B | 9. No Response →GO TO 218B

201B. METHOD = 1: In the last survey, you said that you use a method to delay or avoid pregnancy. Do you still use a method (any method)?

1. Yes →GO TO 202 | 2. No ↓ | 8. Do not Know ↓ | 9. No Response ↓

201C. Why did you stop using a method (any method) to delay pregnancy?

1. I wanted to become pregnant →GO TO 218A | 2. I did not need it / I was not sexually active →GO TO 218A

3. The method was no longer available →GO TO 218A | 4. I had side effects →GO TO 218A

5. Other-specify (201CS. Specify (String 100)) →GO TO 218A | 8. Do not Know →GO TO 218A

9. No Response →GO TO 218A

201D. In the last survey, you said that you do not use any method to delay or avoid pregnancy. Have you started using a method (any method) since the last survey, that is, from harvest time at the time of cold?

1. Yes ↓ | 2. No →GO TO 218A | 8. Do not Know →GO TO 218A | 9. No Response →GO TO 218A

202. What method are you using?

- RECORD ALL MENTIONED.
- IF MORE THAN ONE METHOD IS MENTIONED, FOLLOW THE INSTRUCTIONS BEGINNING WITH THE FIRST METHOD FROM THE LIST.

1. Female Sterilization	2. Male Sterilization	3. IUD	4. Implants	5. Injectables	6. Pill	7. Condom	8. Female Condom
9. Diaphragm	10. Foam / Jelly / Spermicidal Tablet	11. Necklace (Standard Days Method)	12. LAM (Lactational Amenorrhea Method)				
13. Rhythm Method	14. Withdrawal	15. Other method-specify (202S. Specify (String 100))	88. Do not Know	99. No Response			

203. [CURRENT METHOD] = RESPONSE TO 202
Since what month and year have you been using [CURRENT METHOD] without stopping?

- Instruct the participant to estimate if she does not recall the exact date
- RECORD month
- If unknown, ENTER "88"
- If no response, ENTER "99"

00-99

- ENTER year
- If unknown, ENTER "8888"
- If no response, ENTER "9999"

2000-9999

204A. [FIRST METHOD] = FIRST RESPONSE TO 202, [DATE 203] = RESPONSE TO 203
You started using [FIRST METHOD] in [DATE 203]. Where did you get it at that time?

- If primary health center, SPECIFY which primary health center

1. At home	→GO TO 205
2. National Hospital	→GO TO 205
3. Regional Hospital	→GO TO 205
4. Community Referral Hospital (Bankass)	→GO TO 205
5. Primary health center-specify (204AS. Specify (String 100))	→GO TO 205
6. Community Health Worker	→GO TO 204B
7. Hospital / Private Clinic	→GO TO 205
8. Shop	→GO TO 205
9. Nightclub / kiosk / bar	→GO TO 205
10. Street Vendor	→GO TO 205
11. Acquaintance / Friend / Parent	→GO TO 205
12. Other-specify (204AS. Specify (String 100))	→GO TO 205
88. Do not Know	→GO TO 205
99. No Response	→GO TO 205

204B. Where did you meet the CHW to get the method at that time?

1. At respondent's house	2. CHW Site	3. Other-specify (204BS. Specify (String 100))	8. Do not Know	9. No Response

205. Has anyone referred you to this place for family planning?

1. Yes ↓ | 2. No →GO TO 207 | 8. Do not Know →GO TO 207 | 9. No Response →GO TO 207

206. If so, who referred you to this place?

- RECORD ALL MENTIONED

1. CHW	2. A friend	3. Husband	4. Another member of the family	5. A health worker from primary health center
6. Other-specify (206S. Specify (String 100))	88. Do not Know	99. No Response		

207. [CURRENT METHOD] = RESPONSE TO 202

Who advised you to use [CURRENT METHOD] family planning?

- RECORD ALL MENTIONED

1. Respondent	2. Husband	3. CHW	4. Health worker at primary health center	5. Nightclub / kiosk / bar
6. Street vendor	7. Acquaintance / friend / parent	8. Other-specify (207S. Specify (String 100))	88. Do not Know	99. No Response

→GO TO 208B

208A. [CURRENT METHOD] = RESPONSE TO 202
Where did you get [CURRENT METHOD] last time?

- If primary health center, SPECIFY which primary health center

1. At home →GO TO 209
2. National Hospital →GO TO 209
3. Regional Hospital →GO TO 209
4. Community Referral Hospital (Bankass) →GO TO 209
5. Primary health center-specify (208AS. Specify (String 100)) →GO TO 209
6. Community Health Worker →GO TO 208B
7. Hospital / Private Clinic →GO TO 209
8. Shop →GO TO 209
9. Nightclub / kiosk / bar →GO TO 209
10. Street Vendor →GO TO 209
11. Acquaintance / Friend / Parent →GO TO 209
12. Other-specify (208AS. Specify (String 100)) →GO TO 209
88. Do not Know →GO TO 209
99. No Response →GO TO 209

208B. Where did you meet the CHW to get the method last time?

1. At respondent's house →GO TO 214
2. CHW Site ↓
3. Other-specify (208BS. Specify (String 100)) ↓
8. Do not Know ↓
9. No Response ↓

209. [CURRENT METHOD] = RESPONSE TO 202
In total, how much did you pay to get to the health center where you got [CURRENT METHOD] the last time?

- ENTER the value in silver
- If not paid, ENTER "7777"
- If unknown, ENTER "8888"
- If no response, ENTER "9999"

0000-9999

210. [CURRENT METHOD] = RESPONSE TO 202

Approximately how long it takes to get to the health center where you got [CURRENT METHOD] the last time?

1. Less than an hour (less than the time between fitri and saafo) | 2. 1 hour (time between fitri and saafo) | 3. 2 hours (time between salifana and star)

4. ½ day | 5. 1 day | 6. 2 or more days | 88. Do not Know | 99. No Response

211. How long does the time it takes to get to the health center seem to you?

1. Very long | 2. Long | 3. Normal | 4. Short | 5. Very short | 88. Do not Know | 99. No Response

212. Once at the health center, how long did you wait before receiving care?

1. Less than an hour (less than the time between fitri and saafo) | 2. 1 hour (time between fitri and saafo) | 3. 2 hours (time between salifana and star)

4. ½ day | 5. 1 day | 6. 2 or more days | 88. Do not Know | 99. No Response

213. How long does the waiting time seem to you?

1. Very long | 2. Long | 3. Normal | 4. Short | 5. Very short | 88. Do not Know | 99. No Response

214. How long does the family planning consultation take?

1. Less than an hour (less than the time between fitri and saafo) | 2. 1 hour (time between fitri and saafo) | 3. 2 hours (time between salifana and star)

4. ½ day | 5. 1 day | 6. 2 or more days | 88. Do not Know | 99. No Response

215. How much did you pay for the family planning method?

- ENTER value in silver (final digit must be 5 or 0):
- If not paid, ENTER "7777"
- If unknown, ENTER "8888"
- If no response, ENTER "9999"

0000-9999

216. In addition to what you told me, do you have other expenses related to this family planning method?

1. Yes ↓ | 2. No →GO TO 218A | 8. Do not Know →GO TO 218A | 9. No Response →GO TO 218A

217A. How much have you spent on other medications

- ENTER value
- If nothing, ENTER “0”
- If unknown, ENTER “8888”
- If no response, ENTER “9999”

0000-9999

217B. How much have you spent on food and accommodations?

- ENTER value
- If nothing, ENTER “0”
- If unknown, ENTER “8888”
- If no response, ENTER “9999”

0000-9999

217C. How much have you spent on other expenses

- ENTER value
- If nothing, ENTER “0”
- If unknown, ENTER “8888”
- If no response, ENTER “9999”

0000-9999

218A. From the last survey to now, that is, since the time of harvest at the time of cold, were you interested in learning the methods of family planning?

[1. Yes] →GO TO 219 [2. No] →GO TO 219 [8. Do not Know] →GO TO 219 [9. No Response] →GO TO 219

218B. Were you interested in learning about family planning methods?

[1. Yes] [2. No] [8. Do not Know] [9. No Response]

219. Have you consulted with someone to learn more?

[1. Yes] [2. No] →GO TO 221 [8. Do not Know] →GO TO 221 [9. No Response] →GO TO 221

↓

220A. Who did you consult with to learn more?

1. Respondent →GO TO 221 | 2. Husband →GO TO 221 | 3. CHW →GO TO 220B
4. Health worker at primary health center →GO TO 221 | 5. Nightclub / kiosk / bar →GO TO 221
6. Street vendor → GO TO 221 | 7. Acquaintance / friend / parent →GO TO 221
8. Other-specify (220AS. Specify (String 100)) →GO TO 221 | 88. Do not Know →GO TO 221 | 99. No Response →GO TO 221

220B. Where did you consult with the CHW?

1. At respondent's house | 2. CHW Site | 3. Other-specify (220BS. Specify (String 100)) | 8. Do not Know | 9. No Response

221. Over the past 12 months, did you receive a visit from a community health worker who told you about family planning?

1. Yes ↓ | 2. No →GO TO 228 | 8. Do not Know →GO TO 228 | 9. No Response →GO TO 228

222. When was the last visit of the community health worker to talk about family planning?

- ENTER Value

0-50

- ENTER Unit

1. Days | 2. Weeks | 3. Months

223. What type of health worker visited you to talk about family planning.

- RECORD ALL MENTIONED

1. Doctor | 2. Nurse | 3. Midwife | 4. Matron | 5. CHW | 6. Traditional birth attendant
7. Other-specify (223S. Specify (String 100)) | 88. Do not Know | 99. No Response

224. Did the health worker tell you about other family planning methods that you could use?

1. Yes | 2. No | 8. Do not Know | 9. No Response

225. During the family planning consultation, did the health worker offer you a pregnancy test?

1. Yes | 2. No | 8. Do not Know | 9. No Response

→GO TO 227 →GO TO 227 →GO TO 227

↓

226. If you have had a negative pregnancy test, did the health worker propose family planning?

1. Yes	2. No	3. I had a positive pregnancy test	8. Do not Know	9. No Response

227. Were you satisfied with the family planning services delivered by the health worker?

1. Not satisfied	2. Neutral	3. Satisfied	8. Do not Know	9. No Response

2B. Pregnancy and Childbirth

228. Are you pregnant now?

1. Yes	2. No	→GO TO 230	8. Do not Know	→GO TO 230	9. No Response	→GO TO 230

↓

229. How many months pregnant are you?
- RECORD NUMBER OF COMPLETED MONTHS.
- If unknown, ENTER "88"
- If no response, ENTER "99"

0-99

230. When did your last menstrual period start?
- *Encourage the participant to make her best estimate if she does not know the exact number.*
- If menopause or hysterectomy, ENTER "94"
- If before last birth, ENTER "95"
- If never menstruated, ENTER "96"
- If unknown, ENTER "88"
- If no response, ENTER "99"
- ENTER value

0-99

- ENTER unit

1. Days	2. Weeks	3. Months

231A. Since the last survey has now, ie, from the time of harvest at the time of cold, did you give birth to a child who is alive or dead?

1. Yes	2. No	→GO TO 301	3. Never been pregnant	→GO TO 301	8. Do not Know	→GO TO 301	9. No Response	→GO TO 301

↓

231B. What name was given to your last child?
- ENTER name of child

NAME (String 100)

232INTRO. [NAME] = RESPONSE TO 231
Now I want to ask you questions about your pregnancy with [NAME]

- ENTER [1] to continue

1. Continue

232. Did you have a test to confirm the pregnancy?

1. Yes ↓ | 2. No →GO TO 236 | 8. Do not Know →GO TO 236 | 9. No Response →GO TO 236

233. Who gave you the first test to confirm pregnancy?

1. Doctor | 2. Nurse | 3. Midwife | 4. Matron | 5. CHW | 6. Traditional birth attendant | 7. Other-specify (233S. Specify (String 100))
88. Do not Know | 99. No Response

234. Where did you do your pregnancy test?

1. At home | 2. In a health center | 3. Other-specify (234S. Specify (String 100))

235. How much did you pay for your pregnancy test?

- ENTER value in FCFA (Final digit must be 5 or 0)
- If not paid, ENTER “0”
- If unknown, ENTER “8888”
- If no response, ENTER “9999”

0-9999

236. Did you see anyone for antenatal care for this pregnancy?

1. Yes ↓ | 2. No →GO TO 243 | 8. Do not Know →GO TO 243 | 9. No Response →GO TO 243

237. Whom did you see?

- Anyone else?
- PROBE TO IDENTIFY EACH TYPE OF PERSON AND RECORD ALL MENTIONED.

1. Doctor | 2. Nurse | 3. Midwife | 4. Matron | 5. CHW | 6. Traditional birth attendant

7. Other-specify (237S. Specify (String 100)) | 88. Do not Know | 99. No Response

238. Where did you receive antenatal care for this pregnancy?

- Anywhere else?
- PROBE TO IDENTIFY THE TYPE OF SOURCE.

1. Your home | 2. Other house | 3. National Hospital | 4. Regional Hospital | 5. District Hospital

6. Primary health center-specify (238S. Specify (String 100)) | 7. Clinic / Private Hospital | 8. Pharmacy

9. Other-specify (238S. Specify (String 100)) | 88. Do not Know | 99. No Response

239A. Who referred you to get antenatal care?

1. Respondent →GO TO 240 | 2. Husband →GO TO 240 | 3. CHW →GO TO 239B

4. Health worker at primary health center →GO TO 240 | 5. Nightclub / kiosk / bar →GO TO 240

6. Street vendor → GO TO 240 | 7. Acquaintance / friend / parent →GO TO 240

8. Other-specify (239AS. Specify (String 100)) →GO TO 240 | 88. Do not Know →GO TO 240 | 99. No Response →GO TO 240

239B. Where was the reference by the CHW made?

1. At respondent's house | 2. CHW Site | 3. Other-specify (239BS. Specify (String 100)) | 8. Do not Know | 9. No Response

240. How many months pregnant were you when you first received antenatal care for this pregnancy?

- ENTER value in months
- If unknown, ENTER “88”
- If no response, ENTER “99”

0-99

241. How many times did you receive antenatal care during this pregnancy?

- ENTER value
- If unknown, ENTER “88”
- If no response, ENTER “99”

0-99

242. During one of these prenatal visits, were you told about danger signs, warning signs, or complications in pregnancy?

1. Yes	2. No	8. Do not Know	9. No Response

243. During this pregnancy, did you take medicine to prevent malaria?

1. Yes	2. No →GO TO 247	8. Do not Know	9. No Response
↓			

244. What medications did you take?

- RECORD ALL MENTIONED.
- IF TYPE OF MEDICINE IS NOT DETERMINED, SHOW ALL CURRENT ANTIMALARIAL IN THE SURVEY TO RESPONDENT.

1. SP / Fansidar	2. Chloroquine	3. Other-specify (244S. Specify (String 100))	88. Do not Know	99. No Response
↓	→ GO TO 247			

245. How many times did you take SP/Fansidar during this pregnancy?

- ENTER number of times
- If unknown, ENTER “88”
- If no response, ENTER “99”

0-99

246. Did you get the SP / Fansidar under supervision of medical staff at the health center?

1. Yes	2. No	8. Do not Know	9. No Response

247. [NAME] = RESPONSE TO 231
Who assisted with the delivery of [NAME]?

- Anyone else ?
- PROBE FOR THE TYPE(S) OF PERSON(S) AND RECORD ALL MENTIONED.
- IF RESPONDENT SAYS NO ONE ASSISTED, PROBE TO DETERMINE WHETHER ANY ADULTS WERE PRESENT AT THE DELIVERY.

1. Doctor	2. Nurse	3. Midwife	4. Matron	5. CHW	6. Traditional birth attendant	7. Friend/Parent	8. Respondent (alone)
9. Other-specify (247S. Specify (String 100))	88. Do not Know	99. No Response					

248. [NAME] = RESPONSE TO 231
Where did you give birth to [NAME]?

- PROBE TO IDENTIFY THE TYPE OF SOURCE.
- IF UNABLE TO DETERMINE IF PUBLIC OR PRIVATE SECTOR, WRITE THENAME OF THE PLACE

NAME OF PLACE (String 100)

1. Her home	→GO TO 252
2. Other home	→GO TO 249A
3. National hospital	→GO TO 249A
4. Regional hospital	→GO TO 249A
5. Community referral hospital (Bankass)	→GO TO 249A
6. Maternity home	→GO TO 249A
7. Primary Health Center	→GO TO 249A
8. Other public sector	→GO TO 249A
9. Private Clinic / Hospital	→GO TO 249A
10. Office of private care	→GO TO 249A
11. Treatment Room	→GO TO 249A
12. Pharmacy	→GO TO 249A
13. Other private sector	→GO TO 249A
14. Transit	→GO TO 249A
15. Other-specify (248S. Specify (String 100))	→GO TO 249A
88. Do not Know	→GO TO 249A
99. No Response	→GO TO 249A

249A. Who referred you to this place for the birth?

- RECORD ALL MENTIONED

1. Respondent →GO TO 250 | 2. Husband →GO TO 250 | 3. CHW →GO TO 249B

4. Health worker at primary health center →GO TO 250 | 5. Nightclub / kiosk / bar →GO TO 250

6. Street vendor → GO TO 250 | 7. Acquaintance / friend / parent →GO TO 250

8. Other-specify (249AS. Specify (String 100)) →GO TO 250 | 88. Do not Know →GO TO 250 | 99. No Response →GO TO 250

249B. Where was the reference by the CHW made?

1. At respondent's house | 2. CHW Site | 3. Other-specify (249BS. Specify (String 100)) | 8. Do not Know | 9. No Response

250. [NAME] = RESPONSE TO 231
How long after (NAME) was delivered did you stay there?

- ENTER value
- If unknown, ENTER "88"
- If no response, ENTER "99"

0-99

- ENTER unit

1. Hours | 2. Days | 3. Weeks

251. [NAME] = RESPONSE TO 231
Was (NAME) delivered by caesarean, that is, did they cut your belly open to take the baby out?

1. Yes | 2. No | 8. Do not Know | 9. No Response

252. I would like to talk to you about checks on your health after delivery, for example, someone asking you questions about your health or examining you. Did anyone check on your health while you were still in the facility?

1. Yes ↓ | 2. No →GO TO 256 | 8. Do not Know →GO TO 256 | 9. No Response →GO TO 256

253. Who checked on your health at that time?

- RECORD ALL MENTIONED

- PROBE for most qualified person

1. Doctor | 2. Nurse | 3. Midwife | 4. Matron | 5. CHW | 6. Traditional birth attendant

7. Other-specify (253S. Specify (String 100)) | 88. Do not Know | 99. No Response

254. Where did the check take place?

- If primary health center, SPECIFY which one

1. Her home | 2. Other home | 3. National hospital | 4. Regional hospital | 5. Community referral hospital (Bankass)

6. Maternity home | 7. Primary health center-specify (254S. Specify (String 100)) | 8. Other public sector | 9. Private Clinic / Hospital

10. Office of private care | 11. Treatment Room | 12. Pharmacy | 13. Other private sector | 14. Transit

15. Other-specify (254S. Specify (String 100)) | 88. Do not Know | 99. No Response

255. How long after delivery did the first check take place?

- ENTER value
- If unknown, ENTER “88”
- If no response, ENTER “99”

0-99

- ENTER unit

1. Hours | 2. Days | 3. Weeks

256. [NAME] = RESPONSE TO 231
In the two months after [NAME] was born, did a health care provider or a traditional birth attendant check on [NAME]’s health?

1. Yes, health professional (doctor, nurse, midwife) →GO TO 257 | 2. Yes, traditional midwife →GO TO 257 | 3. No →GO TO 260

88. Do not Know →GO TO 260 | 99. No Response →GO TO 260

257. [NAME] = RESPONSE TO 231
How long after delivery was [NAME]’s health first checked?

- ENTER value
- If unknown, ENTER “88”
- If no response, ENTER “99”

0-99

- ENTER unit

1. Hours	2. Days	3. Weeks

258. Who checked on (NAME)’s health at that time?
- RECORD ALL MENTIONED
- PROBE for most qualified person

1. Doctor	2. Nurse	3. Midwife	4. Matron	5. CHW	6. Traditional birth attendant
7. Other-specify (258S. Specify (String 100))	88. Do not Know	99. No Response			

259. Where did the check take place?
- If primary health center, SPECIFY which one

1. Her home	2. Other home	3. National hospital	4. Regional hospital	5. District Hospital
6. Primary health center-specify (259S. Specify (String 100))	7. Private Clinic / Hospital	8. Pharmacy		
9. Other-specify (259S. Specify (String 100))	88. Do not Know	99. No Response		

260. [NAME] = RESPONSE TO 231
Did you ever breastfeed [NAME]?

1. Yes	2. No →GO TO 301	8. Do not Know →GO TO 301	9. No Response →GO TO 301

↓

261. [NAME] = RESPONSE TO 231
Are you still breastfeeding [NAME]?

1. Yes	2. No	9. No Response

261A. [NAME] = RESPONSE TO 231
Are you still breastfeeding [NAME] exclusively?

1. Yes	→GO TO 301	2. No	9. No Response

262. [NAME] = RESPONSE TO 231
If not, for how long did you breastfeed [NAME]?

- ENTER value

0-99

- ENTER unit

1. Day	2. Month	3. Year

263. For how long did you only breastfeed (NAME), that is to say, without other things to drink?

- ENTER value

0-99

- ENTER unit

1. Day	2. Month	3. Year

Section 3: Children, Survival and Mortality

301. I would like to confirm, have you ever given birth to children?

1. Yes ↓ | 2. No →GO TO 304

302. Do you have sons or daughters to whom you gave birth who are alive?

1. Yes ↓ | 2. No →GO TO 304

303A. How many sons are alive? IF NONE, ENTER "00"

00-20

303B. And how many girls are alive? IF NONE, ENTER "00"

00-20

303C. THE NUMBER OF LIVING CHILDREN

00-20

304. Have you ever given birth to a boy or girl who was born alive but later died?

- IF NO, PROBE: Any baby who cried, who made any movement, sound, or effort to breathe, or who showed any other signs of life even if for a very short time?

1. Yes ↓ | 2. No →GO TO 306A | 9. No Response ↓

305A. How many boys have died? IF NONE, ENTER "00"

00-20

305B. How many girls have died? IF NONE, ENTER "00"

00-20

305C. THE NUMBER OF DECEASED CHILDREN

==305A+305B

305D. The number of children living elsewhere who were not counted in the past year during the baseline survey

00-20

306A. ADD ANSWERS TO 303C AND 305C, AND ENTER THE TOTAL.

- IF NONE, RECORD '00'.

==303C+305C

306B. [___] = RESPONSE TO 306A
Just to make sure that I have this right: you have had in TOTAL [_____] births during your life. Is that correct?

1. Yes →GO TO 307 | 2. No ↓

306BCKPT.

1. If 306A==0 AND 306B==1 →GO TO 319 | 2. ALL OTHERS ↓

306C. If Q306B = no, enter the correct number of living children

00-20

306D. If Q306B = no, enter the correct number of deceased children

00-20

306E. If Q306A = no, enter the correct number of total children

00-20

307INTRO. List all the births of women including those born alive and who later died. Even children who have died knowing their baptism are concerned.

- ENTER [1] to continue

1. Continue

Birth History Loop (Max = 30)

307. What was the first and family name was given to your [first / next] baby?

- ENTER THE FIRST NAME.

FIRSTNAME (String 100)

- ENTER THE FAMILY NAME

FAMILYNAME (String 100)

308. Was this birth twins?

- If yes, drop-down of all children listed under the woman, with a field to add a new one.
- Ensure that 307 to 315 is collected for TWO children.

1. Yes	2. No

309. [NAME] = RESPONSE TO 307
Is [NAME] a boy or girl?

1. Boy	2. Girl

310A. [NAME] = RESPONSE TO 307
On what day, month, and year was [NAME] born?

- ENTER Day
- If unknown, ENTER "88"
- If no response, ENTER "99"

01-31

- ENTER Month
- If unknown, ENTER "88"
- If no response, ENTER "99"

01-12

- ENTER Year
- If unknown, ENTER "8888"
- If no response, ENTER "9999"

1950-2100

310B. Source of Information

1. Memory	2. Official Document

311. Is he/she still alive?

1. Yes	2. No

→GO TO 313

↓

311A. IF ALIVE: Does he/she live with you?

1. Yes	2. No

312. [NAME] = RESPONSE TO 307
IF ALIVE: How old was [NAME] on his/her last birthday?

- Enter the age in years.
- Mark 00 years if the child is less than one year
- ENTER years:
- If unknown, ENTER "88"
- If no response, ENTER "99"

0-99

313. [NAME] = RESPONSE TO 307
IF DECEASED: What was the date of death of [NAME]?

- Mark MONTH and YEAR. The two fields are mandatory
- ENTER year as ("YYYY"), 4 digits, like 1990
- If unknown, ENTER "8888"
- If no response, ENTER "9999"

0000-9999

- ENTER month as ("MM"), 2 digits, 1 to 12
- If unknown, ENTER "88"
- If no response, ENTER "99"

00-99

313A. [NAME] = RESPONSE TO 307
During what season did [NAME] die?

1. Heat (March, April, May)	2. Sowing of millet / early rain (June)	3. Sowing peanut and maize / first crop (July)	4. Chocho / heavy rain / second crop (August)
5. Corn harvest (September)	6. Harvest of millet and peanuts (October, November)	7. The Cold (December, January, February)	

314. [NAME] = RESPONSE TO 307, HE/SHE = [BOY / GIRL]
IF DECEASED: How old was [NAME] when [he / she] died?

- Enter the age in years, months and days.
- All fields are required.
- Mark 00 years if the child was less than one year, 00 months if the child was less than one month
- IF "12 months" OR "1 YEAR", PROBE: Did (NAME) have her first birthday?
- THEN ASK: Exactly how many nois had (NAME) when (he / she) died?
- RECORD AGE IN NUMBER OF YEARS, MONTHS AND DAYS. ALL FIELDS ARE MANDATORY. MARK 00 YEARS IF 'LENFANT LESS THAN ONE YEAR, 00 MONTH IF CHILD LESS THAN ONE MONTH).
  - ENTER years:
  - If unknown, ENTER "88"
  - If no response, ENTER "99"

    [0-99]

  - ENTER months:
  - If unknown, ENTER "88"
  - If no response, ENTER "99"

    [0-99]

  - ENTER days:
  - If unknown, ENTER "88"
  - If no response, ENTER "99"

    [0-99]

315. [NAME OF PREVIOUS BIRTH] = RESPONSE TO 307_X-1 [NAME] = RESPONSE TO 307_X
Were there any other live births between [NAME OF PREVIOUS BIRTH] and [NAME], including any children who died after birth?

[1. Yes] →CHOOSE CHILD FROM THE LIST [2. No] ↓

<u>End of Birth History Loop</u>

318CKPT. ODK CHECK that the total number of births (305 if 306A == 1; 306B if 306A == 2) is not greater than the number of children in the birth list (307 to 315)

1. TOTAL NUMBER OF BIRTHS (305 IF 306A == 1; 306A == 2) IS GREATER THAN THE NUMBER OF CHILDREN IN THE BIRTH LIST (307 TO 315) ↓	2. TOTAL NUMBER OF BIRTHS (305 IF 306A == 1; 306A == 2) IS NOT GREATER THAN THE NUMBER OF CHILDREN IN THE BIRTH LIST (307 TO 315)	→ GO TO 319

318A. [X] = (306A if 306B == 1 OR 306E IF 306B == 2)
Before, you told me that you had [X] births during your life. You told me about the births of (LIST THE NAMES OF CHILDREN IN 307). Have you had other births during your life, including newborns who were born alive but later died?

1. Yes →COLLECT 307 TO 316 FOR ADDITIONAL CHILDREN

2. No ↓

318B. Record the correct number of births of children still alive.

00-20

318C. Record the correct number of births of children who were born alive but who died not after

00-20

318D. Record the correct number of total births in the woman's life.

00-20

319. Have you ever had a pregnancy that ended in a miscarriage, abortion or stillbirth?

1. Yes

2. No →GO TO 401

320. Among these completed pregnancies in these ways, were there: Miscarriages? Abortions? Stillbirths?

- Miscarriage

1. Yes	2. No

- Abortion

1. Yes	2. No

- Stillbirth

1. Yes	2. No

321. When did the last pregnancy of this kind end? (In which month and year?)

- Mark MONTH and YEAR. The two fields are mandatory
- ENTER month as “MM”
- If unknown, ENTER “88”
- If no response, ENTER “99”

00-99

  - ENTER year as “YYYY”
  - If unknown, ENTER “8888”
  - If no response, ENTER “9999”

0000-9999

322. How many months were you pregnant when the last pregnancy of this kind ended?

0-9

323. How did it end?

1. Miscarriage	2. Abortion	3. Stillbirth

324. Have you had other pregnancies that did not result in a live birth?

1. Yes	2. No

32[illegible] d [illegible]ancy end?

- Mark MONTH and YEAR. The two fields are mandatory
- ENTER month as “MM”
- If unknown, ENTER “88”
- If no response, ENTER “99”

00-99

- ENTER year as “YYYY”
- If unknown, ENTER “8888”
- If no response, ENTER “9999”

0000-9999

326. How many months were you pregnant when the last pregnancy of this kind ended?

0-9

327. How did it end?

1. Miscarriage | 2. Abortion | 3. Stillbirth

328. Have you had other pregnancies that did not result in a live birth?

1. Yes | 2. No

329. When did this pregnancy end?

- Mark MONTH and YEAR. The two fields are mandatory
- ENTER month as “MM”
- If unknown, ENTER “88”
- If no response, ENTER “99”

00-99

- ENTER year as “YYYY”
- If unknown, ENTER “8888”
- If no response, ENTER “9999”

0000-9999

330. How ma[illegible]re you pregnant when the last pregnancy of this kind ended?

0-9

331. How did it end?

1. Miscarriage	2. Abortion	3. Stillbirth

332. Have you had other pregnancies that did not result in a live birth?

1. Yes	2. No

333. When did this pregnancy end?

- Mark MONTH and YEAR. The two fields are mandatory
- ENTER month as "MM"
- If unknown, ENTER "88"
- If no response, ENTER "99"

00-99

- ENTER year as "YYYY"
- If unknown, ENTER "8888"
- If no response, ENTER "9999"

0000-9999

334. How many months were you pregnant when the last pregnancy of this kind ended?

0-9

335. How did it end?

1. Miscarriage	2. Abortion	3. Stillbirth

VERIFY: If there are more pregnancies completed, use a second survey to the point that all completed pregnancies are recorded.

336. IF NO BIRTH, DEATH, MISCARRIAGE, ABORTION, DEATH AMONG THE WOMEN WHO WERE PREGEDED IN THE PREVIOUS SURVEY:

In the last survey, you said that you were pregnant. What was the outcome of this pregnancy?

1. Live Birth	→GO TO 307	2. Stillbirth	3. Termination (miscarriage, abortion)

Section 4: Diseases Of Children

Diarrhea

401. [NAME]
Has [NAME] had diarrhea in the last 2 weeks?

1. Yes | 2. No →GO TO 500 | 8. Do not Know →GO TO 500 | 9. No Response →GO TO 500

402. Was there blood in the stool?

1. Yes | 2. No | 8. Do not Know | 9. No Response

403. Did you seek advice or treatment for the diarrhea from any source?

1. Yes →GO TO 405 | 2. No ↓ | 8. Do not Know ↓ | 9. No Response ↓

404. Why did you not get advice or treatment?

- PROBE: Other reasons?
- RECORD ALL MENTIONED

1. I could not pay the fee
2. I could not cross the distance to the health center
3. I do not make decisions about seeking health care
4. I arrived at the health center, but there was no equipment, material, or infrastructure to give me care
5. The prescribed medication was not available
6. I arrived at the health center, but I did not find staff qualified enough to give me care
7. I did not know what to do
8. I did not think it was serious enough
9. I did not have enough time
10. Other-specify (404S. Specify (String 100))
88. Do not Know
99. No Response

→GO TO 500

405. Where did you seek advice or treatment? Please list all sources in the order that you got them.

- PROBE: Anywhere else?
- PROBE TO IDENTIFY THE SOURCE
- MARK 1, 2, 3 FOR THE ORDER TO VISIT
- IF ONLY 1 (OR 2) LOCATIONS, MARK "WITHOUT RESPONSE" FOR 2ND (OR 3RD) LOCATION
- If primary health center, SPECIFY which one

1. At my house	2. National hospital	3. Regional hospital	4. Community referral hospital (Bankass)

5. Primary health center-specify (405S. Specify (String 100))	6. Private Clinic / Hospital	7. Pharmacy	8. At CHW	9. Home of CHW

10. At a traditional healer	11. At home, a travelling seller	12. Shop	13. Other-specify (405S. Specify (String 100))

88. Do not Know	99. No Response

406. HE/SHE = [BOY / GIRL]
Did someone evaluate the child before [he / she] went to the health center? If so, who was it?

- 0. No →GO TO 410
- 1. Myself →GO TO 410
- 2. Husband →GO TO 410
- 3. Other women →GO TO 410
- 4. Other close relatives →GO TO 410
- 5. Friends / neighbors →GO TO 410
- 6. CHW →GO TO 407
- 7. Doctor →GO TO 410
- 8. Nurse →GO TO 410
- 9. Midwife →GO TO 410
- 10. Matron →GO TO 410
- 11. Traditional healer →GO TO 410
- 12. Drug seller →GO TO 410
- 13. Other-specify (406S. Specify (String 100)) →GO TO 410
- 88. Do not Know →GO TO 410
- 99. No Response →GO TO 410

407. Where was the evaluation conducted?

- 1. At home ↓
- 2. CHW Site →GO TO 409
- 3. Other-specify (407S. Specify (String 100)) ↓
- 8. Do not Know ↓
- 9. No Response ↓

408. How much time does it seem to take the CHW to arrive at your house?

1. Very long	2. Long	3. Normal	4. Short	5. Very short	88. Do not Know	99. No Response

409. How long did the evaluation last?

1. Less than an hour (less than the time between fitri and saafo)	2. 1 hour (time between fitri and saafo)	3. 2 hours (time between salifana and star)

4. ½ day	5. 1 day	6. 2 or more days	88. Do not Know	99. No Response

410. How much time did it seem to take to get to the health center?

- If you went to several health centers, this is the last facility you attended.

1. Very long	2. Long	3. Normal	4. Short	5. Very short	88. Do not Know	99. No Response

411. At the health center, how long did it take before you received treatment?

1. Less than an hour (less than the time between fitri and saafo)	2. 1 hour (time between fitri and saafo)	3. 2 hours (time between salifana and star)

4. ½ day	5. 1 day	6. 2 or more days	88. Do not Know	99. No Response

412. How did the waiting time seem to you?

1. Very long	2. Long	3. Normal	4. Short	5. Very short	88. Do not Know	99. No Response

413. How long did the medical consultation take?

1. Less than an hour (less than the time between fitri and saafo)	2. 1 hour (time between fitri and saafo)	3. 2 hours (time between salifana and star)

4. ½ day	5. 1 day	6. 2 or more days	88. Do not Know	99. No Response

414A. Did you take time off work to bring your child to the clinic? That is to say, the work you do that generates income.

1. Yes	2. No	8. Do not Know	9. No Response

414B. If so, how many days?

- If no leave taken, ENTER 0
- If unknown, ENTER 88
- If no response, ENTER 99

0-99

415. While seeking care for your child, which of the following expenses did you obtain?

1. Medication / treatment

1. Yes	2. No	8. Do not Know	9. No Response

2. Laboratory / diagnostic tests

1. Yes	2. No	8. Do not Know	9. No Response

3. Other health expenses

1. Yes	2. No	8. Do not Know	9. No Response

4. Food / Accommodation

1. Yes	2. No	8. Do not Know	9. No Response

5. Other expenses

1. Yes	2. No	8. Do not Know	9. No Response

415CKPT.

1. If ALL 415==1 ↓	2. If ANY 415==2, 8, or 9	→GO TO 417

416. If Yes: How much did you pay for all these things?

- ENTER value in FCFA
- Value must end in 5 or 0 (an estimation)
- If unknown, ENTER 8888
- If no response, ENTER 9999

0-9999

417. What treatment did your child receive?

- RECORD ALL MENTIONED

1. ORS	2. Antibiotic tablet or syrup	3. Antimotility tablet or syrup	4. Zinc tablet or syrup

5. Other / unknown tablet or syrup	6. Injectable antibiotic	7. Non-injectable antibiotic	8. Unknown injection
9. (IV) Intravenous	10. Home remedy / herbal medicine	11. Other-specify (417S. Specify (String 100))	
88. Do not Know	99. No Response		

418A. [NAME], [FIRST DRUG 417] = FIRST RESPONSE FROM 417
How long after the onset of diarrhea, did [NAME] start taking the [FIRST DRUG 417]?

0. Same day	1. Next day	2. Two days after diarrhea	3. Three days or more after diarrhea	88. Do not Know	99. No Response

418B. [NAME], [SECOND DRUG 417] = SECOND RESPONSE FROM 417
How long after the onset of diarrhea, did [NAME] start taking the [SECOND DRUG 417]?

0. Same day	1. Next day	2. Two days after diarrhea	3. Three days or more after diarrhea	88. Do not Know	99. No Response

418C. [NAME], [THIRD DRUG 417] = THIRD RESPONSE FROM 417
How long after the onset of diarrhea, did [NAME] start taking the [THIRD DRUG 417]?

0. Same day	1. Next day	2. Two days after diarrhea	3. Three days or more after diarrhea	88. Do not Know	99. No Response

Fever

500. Has [NAME] been ill with a fever at any time during the last 2 weeks?

1. Yes ↓ | 2. No →GO TO 600 | 8. Do not Know →GO TO 600 | 9. No Response →GO TO 600

501. Did you seek advice or treatment for the fever from any source?

1. Yes →GO TO 503 | 2. No ↓ | 8. Do not Know ↓ | 9. No Response ↓

502. Why did you not get advice or treatment?

- PROBE: Other reasons?
- RECORD ALL MENTIONED

1. I could not pay the fee
2. I could not cross the distance to the health center
3. I do not make decisions about seeking health care
4. I arrived at the health center, but there was no equipment, material, or infrastructure to give me care
5. The prescribed medication was not available
6. I arrived at the health center, but I did not find staff qualified enough to give me care
7. I did not know what to do
8. I did not think it was serious enough
9. I did not have enough time
10. Other-specify (502S. Specify (String 100))
88. Do not Know
99. No Response

→GO TO 600

503. Where did you seek advice or treatment? Please list all sources in the order that you got them.

- PROBE: Anywhere else?
- MARK 1, 2, 3 FOR THE ORDER TO VISIT
- IF ONLY 1 (OR 2) LOCATIONS, MARK "WITHOUT RESPONSE" FOR 2ND (OR 3RD) LOCATION
- PROBE TO IDENTIFY THE SOURCE
- If primary health center, SPECIFY which one

1. At my house | 2. National hospital | 3. Regional hospital | 4. Community referral hospital (Bankass)

5. Primary health center-specify (503S. Specify (String 100)) | 6. Private Clinic / Hospital | 7. Pharmacy | 8. At CHW | 9. Home of CHW

10. At a traditional healer | 11. At home, a travelling seller | 12. Shop | 13. Other-specify (503S. Specify (String 100))

88. Do not Know | 99. No Response

504. At any time during the illness, did (NAME) have blood taken from (NAME)'s finger or heel for testing? If so, who took the blood?

0. No | 1. Myself | 2. Husband | 3. Other women | 4. Other close relatives | 5. Friends / neighbors

6. CHW | 7. Doctor | 8. Nurse | 9. Midwife | 10. Matron | 11. Traditional healer | 12. Drug seller

13. Other-specify (504S. Specify (String 100)) | 88. Do not Know | 99. No Response

505. Did someone evaluate the child before (he/she) went to the health center? If so, who was it?

0. No →GO TO 509 | 1. Myself →GO TO 509 | 2. Husband →GO TO 509 | 3. Other women →GO TO 509

4. Other close relatives →GO TO 509 | 5. Friends / neighbors →GO TO 509 | 6. CHW →GO TO 506 | 7. Doctor →GO TO 509

8. Nurse →GO TO 509 | 9. Midwife →GO TO 509 | 10. Matron →GO TO 509 | 11. Traditional healer → GO TO 509

12. Drug seller →GO TO 509 | 13. Other-specify (505S. Specify (String 100)) →GO TO 509

88. Do not Know →GO TO 509 | 99. No Response →GO TO 509

506. Where was the evaluation conducted?

1. At home →GO TO 507 | 2. CHW Site →GO TO 508 | 3. Other-specify (506S. Specify (String 100)) →GO TO 508

8. Do not Know →GO TO 508 | 9. No Response →GO TO 508

507. How much time does it seem to take the CHW to arrive at your house?

1. Very long | 2. Long | 3. Normal | 4. Short | 5. Very short | 88. Do not Know | 99. No Response

508. How long did the evaluation last?

1. Less than an hour (less than the time between fitri and saafo) | 2. 1 hour (time between fitri and saafo) | 3. 2 hours (time between salifana and star)

4. ½ day | 5. 1 day | 6. 2 or more days | 88. Do not Know | 99. No Response

509. How much time did it seem to take to get to the health center?

- If you went to several health centers, this is the last facility you attended.

1. Very long | 2. Long | 3. Normal | 4. Short | 5. Very short | 88. Do not Know | 99. No Response

510. At the health center, how long did it take before you received treatment?

1. Less than an hour (less than the time between fitri and saafo) | 2. 1 hour (time between fitri and saafo) | 3. 2 hours (time between salifana and star)

4. ½ day | 5. 1 day | 6. 2 or more days | 88. Do not Know | 99. No Response

511. How did the waiting time seem to you?

1. Very long | 2. Long | 3. Normal | 4. Short | 5. Very short | 88. Do not Know | 99. No Response

512. How long did the medical consultation take?

1. Less than an hour (less than the time between fitri and saafo) | 2. 1 hour (time between fitri and saafo) | 3. 2 hours (time between salifana and star)

4. ½ day | 5. 1 day | 6. 2 or more days | 88. Do not Know | 99. No Response

513A. Did you take time off work to bring your child to the clinic? That is to say, the work you do that generates income.

1. Yes | 2. No | 8. Do not Know | 9. No Response

513B. If so, how many days?

- If no leave taken, ENTER 0
- If unknown, ENTER 88
- If no response, ENTER 99

0-99

514. While seeking care for your child, which of the following expenses did you obtain?

1. Medication / treatment

1. Yes	2. No	8. Do not Know	9. No Response

2. Laboratory / diagnostic tests

1. Yes	2. No	8. Do not Know	9. No Response

3. Other health expenses

1. Yes	2. No	8. Do not Know	9. No Response

4. Food / Accommodation

1. Yes	2. No	8. Do not Know	9. No Response

5. Other expenses

1. Yes	2. No	8. Do not Know	9. No Response

514CKPT.

1. If ALL 514==1 ↓	2. If ANY 514==2, 8, or 9 →GO TO 516

515. If Yes: How much did you pay for all these things?

- ENTER value in FCFA
- Last digit must be a 5 or 0 (estimation)
- If not paid, ENTER 0
- If unknown, ENTER 8888
- If no response, ENTER 9999

0-9999

516. What treatment did your child receive?

- RECORD ALL MENTIONED

1. SP / Fansidar	2. Chloroquine	3. Amodiaquine	4. Artemisinin Combination Therapy (ACT)	5. Quinine tablet	6. Quinine injection
7. Artesunate injection	8. Artemether injection	9. Other antipaludienne-specify (516S. Specify (String 100))	10. Paracetamol	11. Ibuprofen	
12. Other-specify (516S. Specify (String 100))	88. Do not Know	99. No Response			

517a. [NAME], [FIRST DRUG 516] = FIRST RESPONSE TO 516
How long after the onset of fever, did (NAME) start taking the [FIRST DRUG 516]?

0. Same day	1. Next day	2. Two days after fever	3. Three days or more after fever	8. Do not Know

517B. [NAME], [SECOND DRUG 516] = SECOND RESPONSE TO 516
How long after the onset of fever, did (NAME) start taking the [SECOND DRUG 516]?

0. Same day	1. Next day	2. Two days after fever	3. Three days or more after fever	8. Do not Know	9. No Response

517C. [NAME], [THIRD DRUG 516] = THIRD RESPONSE TO 516
How long after the onset of fever, did (NAME) start taking the [THIRD DRUG 516]?

0. Same day	1. Next day	2. Two days after fever	3. Three days or more after fever	8. Do not Know	9. No Response

Cough / Difficulty Breathing

600. [NAME]
Has [NAME] been ill with a cough at any time during the last 2 weeks?

1. Yes ↓	2. No →GO TO 700a	8. Do not Know →GO TO 700a	9. No Response →GO TO 700a

601. [NAME]
When [NAME] was ill with a cough, did [NAME] have fast, short, rapid breaths or difficulty breathing at any time?

1. Yes	2. No	8. Do not Know	9. No Response

602. Did you seek advice or treatment for the diarrhea from any source?

1. Yes →GO TO 604	2. No ↓	8. Do not Know ↓	9. No Response ↓

603. Why did you not get advice or treatment?

- PROBE: Other reasons?
- RECORD ALL MENTIONED

1. I could not pay the fee	2. I could not cross the distance to the health center	3. I do not make decisions about seeking health care
4. I arrived at the health center, but there was no equipment, material, or infrastructure to give me care		
5. The prescribed medication was not available	6. I arrived at the health center, but I did not find staff qualified enough to give me care	
7. I did not know what to do	8. I did not think it was serious enough	9. I did not have enough time
10. Other-specify (603S. Specify (String 100))	88. Do not Know	99. No Response

→GO TO 700a

604. Where did you seek advice or treatment? Please list all sources in the order that you got them.

- PROBE: Anywhere else?
- PROBE TO IDENTIFY THE SOURCE
- MARK 1, 2, 3 FOR THE ORDER TO VISIT
- IF ONLY 1 (OR 2) LOCATIONS, MARK "WITHOUT RESPONSE" FOR 2ND (OR 3RD) LOCATION
- If primary health center, SPECIFY which one

1. At my house | 2. National hospital | 3. Regional hospital | 4. Community referral hospital (Bankass)

5. Primary health center-specify (604S. Specify (String 100)) | 6. Private Clinic / Hospital | 7. Pharmacy | 8. At CHW | 9. Home of CHW

10. At a traditional healer | 11. At home, a travelling seller | 12. Shop | 13. Other-specify (604S. Specify (String 100))

88. Do not Know | 99. No Response

605. HE/SHE = [BOY / GIRL]
Did someone evaluate the child before [he / she] went to the health center? If so, who was it?

0. No →GO TO 609 | 1. Myself →GO TO 609 | 2. Husband →GO TO 609 | 3. Other women →GO TO 609

4. Other close relatives →GO TO 609 | 5. Friends / neighbors →GO TO 609 | 6. CHW →GO TO 606 | 7. Doctor →GO TO 609

8. Nurse →GO TO 609 | 9. Midwife →GO TO 609 | 10. Matron →GO TO 609 | 11. Traditional healer →GO TO 609

12. Drug seller →GO TO 609 | 13. Other-specify (605S. Specify (String 100)) →GO TO 609

88. Do not Know →GO TO 609 | 99. No Response →GO TO 609

606. Where was the evaluation conducted?

1. At home ↓ | 2. CHW Site →GO TO 608 | 3. Other-specify (606S. Specify (String 100)) ↓ | 8. Do not Know ↓ | 9. No Response ↓

607. How much time does it seem to take the CHW to arrive at your house?

1. Very long | 2. Long | 3. Normal | 4. Short | 5. Very short | 88. Do not Know | 99. No Response

608. How long did the evaluation last?

1. Less than an hour (less than the time between fitri and saafo)	2. 1 hour (time between fitri and saafo)	3. 2 hours (time between salifana and star)

4. ½ day	5. 1 day	6. 2 or more days	88. Do not Know	99. No Response

609. How much time did it seem to take to get to the health center?

- If you went to several health centers, this is the last facility you attended.

1. Very long	2. Long	3. Normal	4. Short	5. Very short	88. Do not Know	99. No Response

610. At the health center, how long did it take before you received treatment?

1. Less than an hour (less than the time between fitri and saafo)	2. 1 hour (time between fitri and saafo)	3. 2 hours (time between salifana and star)

4. ½ day	5. 1 day	6. 2 or more days	88. Do not Know	99. No Response

611. How did the waiting time seem to you?

1. Very long	2. Long	3. Normal	4. Short	5. Very short	88. Do not Know	99. No Response

612. How long did the medical consultation take?

1. Less than an hour (less than the time between fitri and saafo)	2. 1 hour (time between fitri and saafo)	3. 2 hours (time between salifana and star)

4. ½ day	5. 1 day	6. 2 or more days	88. Do not Know	99. No Response

613A. Did you take time off work to bring your child to the clinic? That is to say, the work you do that generates income.

1. Yes	2. No	8. Do not Know	9. No Response

613B. If so, how many days?

- If no leave taken, ENTER 0
- If unknown, ENTER 88
- If no response, ENTER 99

0-99

614. While seeking care for your child, which of the following expenses did you obtain?

1. Medication / treatment

1. Yes	2. No	8. Do not Know	9. No Response

2. Laboratory / diagnostic tests

1. Yes	2. No	8. Do not Know	9. No Response

3. Other health expenses

1. Yes	2. No	8. Do not Know	9. No Response

4. Food / Accommodation

1. Yes	2. No	8. Do not Know	9. No Response

5. Other expenses

1. Yes	2. No	8. Do not Know	9. No Response

614CKPT.

1. If ALL 614==1	2. If ANY 614==2, 8, or 9	→GO TO 616
↓		

615. If Yes: How much did you pay for all these things?

- ENTER value in FCFA
- Last digit must be 5 or 0 (estimation)
- If not paid, ENTER 0
- If unknown, ENTER 8888
- If no response, ENTER 9999

0-9999

616. What treatment did your child receive?

- RECORD ALL MENTIONED

1. Amoxicillin	2-1. Another antibiotic-specify (616S. Specify (String 100))	2-2. Antitussive

3. Decongestant	4. Local applications (ie, cream, ointment, butter)	5. Antimalarial		
6. Paracetamol	7. Ibuprofen	8. Other-specify (616S. Specify (String 100))	88. Do not Know	99. No Response

617A. [NAME], [FIRST DRUG 616] = FIRST RESPONSE TO 616
How long after the onset of the cough, did [NAME] start taking the [FIRST DRUG 616]?

0. Same day	1. Next day	2. Two days after diarrhea	3. Three days or more after diarrhea	88. Do not Know	99. No Response

617B. [NAME], [SECOND DRUG 616] = SECOND RESPONSE TO 616
How long after the onset of the cough, did [NAME] start taking the [SECOND DRUG 616]?

0. Same day	1. Next day	2. Two days after diarrhea	3. Three days or more after diarrhea	88. Do not Know	99. No Response

617C. [NAME], [THIRD DRUG 616] = THIRD RESPONSE TO 616
How long after the onset of the cough, did [NAME] start taking the [THIRD DRUG 616]?

0. Same day	1. Next day	2. Two days after diarrhea	3. Three days or more after diarrhea	88. Do not Know	99. No Response

Other diseases

700A. [NAME]
Has [NAME] had any other types of illnesses or injuries the last two weeks?

1. Yes	2. No	8. Do not Know	9. No Response
↓	→GO TO 401 FOR NEXT CHILD OR 800 IF NO MORE		

700B. If so, what type of illness or injury?

1. Skin infection (skin)	2. Intestinal parasite	3. Bilharziose (blood in urine)	4. Conjunctivitis (eye infection)	5. Fractures (bone)
6. Wounds	7. Burns	8. Measles	9. Flu	10. Other-specify (700bS. Specify (String 100))

701. Did you seek advice or treatment for the diarrhea from any source?

1. Yes →GO TO 703	2. No	8. Do not Know	9. No Response
	↓	↓	↓

702. Why did you not get advice or treatment?
- PROBE: Other reasons?
- RECORD ALL MENTIONED

1. I could not pay the fee
2. I could not cross the distance to the health center
3. I do not make decisions about seeking health care
4. I arrived at the health center, but there was no equipment, material, or infrastructure to give me care
5. The prescribed medication was not available
6. I arrived at the health center, but I did not find staff qualified enough to give me care
7. I did not know what to do
8. I did not think it was serious enough
9. I did not have enough time
10. Other-specify (702S. Specify (String 100))
88. Do not Know
99. No Response

→GO TO 800

703. Where did you seek advice or treatment? Please list all sources in the order that you got them.

- PROBE: Anywhere else?
- PROBE TO IDENTIFY THE SOURCE
- MARK 1, 2, 3 FOR THE ORDER TO VISIT
- IF ONLY 1 (OR 2) LOCATIONS, MARK "WITHOUT RESPONSE" FOR 2ND (OR 3RD) LOCATION
- If primary health center, SPECIFY which one

1. At my house | 2. National hospital | 3. Regional hospital | 4. Community referral hospital (Bankass)

5. Primary health center-specify (703S. Specify (String 100)) | 6. Private Clinic / Hospital | 7. Pharmacy | 8. At CHW | 9. Home of CHW

10. At a traditional healer | 11. At home, a travelling seller | 12. Shop | 13. Other-specify (703S. Specify (String 100))

88. Do not Know | 99. No Response

704. HE/SHE = [BOY / GIRL]
Did someone evaluate the child before [he/she] went to the health center? If so, who was it?

0. No →GO TO 708 | 1. Myself →GO TO 708 | 2. Husband →GO TO 708 | 3. Other women →GO TO 708

4. Other close relatives →GO TO 708 | 5. Friends / neighbors →GO TO 708 | 6. CHW →GO TO 705 | 7. Doctor →GO TO 708

8. Nurse →GO TO 708 | 9. Midwife →GO TO 708 | 10. Matron →GO TO 708 | 11. Traditional healer →GO TO 708

12. Drug seller →GO TO 708 | 88. Do not Know →GO TO 708 | 99. No Response →GO TO 708

705. Where was the evaluation conducted?

1. At home ↓ | 2. CHW Site →GO TO 707 | 3. Other-specify (705S. Specify (String 100)) ↓ | 8. Do not Know ↓ | 9. No Response ↓

706. How much time does it seem to take the CHW to arrive at your house?

1. Very long | 2. Long | 3. Normal | 4. Short | 5. Very short | 88. Do not Know | 99. No Response

707. How long did the evaluation last?

1. Less than an hour (less than the time between fitri and saafo) | 2. 1 hour (time between fitri and saafo) | 3. 2 hours (time between salifana and star)

4. ½ day | 5. 1 day | 6. 2 or more days | 88. Do not Know | 99. No Response

708. How much time did it seem to take to get to the health center?

- If you went to several health centers, this is the last facility you attended.

1. Very long | 2. Long | 3. Normal | 4. Short | 5. Very short | 88. Do not Know | 99. No Response

709. At the health center, how long did it take before you received treatment?

1. Less than an hour (less than the time between fitri and saafo) | 2. 1 hour (time between fitri and saafo) | 3. 2 hours (time between salifana and star)

4. ½ day | 5. 1 day | 6. 2 or more days | 88. Do not Know | 99. No Response

710. How did the waiting time seem to you?

1. Very long | 2. Long | 3. Normal | 4. Short | 5. Very short | 88. Do not Know | 99. No Response

711. How long did the medical consultation take?

1. Less than an hour (less than the time between fitri and saafo) | 2. 1 hour (time between fitri and saafo) | 3. 2 hours (time between salifana and star)

4. ½ day | 5. 1 day | 6. 2 or more days | 88. Do not Know | 99. No Response

712A. Did you take time off work to bring your child to the clinic? That is to say, the work you do that generates income.

1. Yes | 2. No | 8. Do not Know | 9. No Response

712B. If so, how many days?

- If no leave taken, ENTER 0
- If unknown, ENTER 88
- If no response, ENTER 99

0-99

713. While seeking care for your child, which of the following expenses did you obtain?

1. Medication / treatment

1. Yes | 2. No | 8. Do not Know | 9. No Response

2. Laboratory / diagnostic tests

1. Yes | 2. No | 8. Do not Know | 9. No Response

3. Other health expenses

1. Yes | 2. No | 8. Do not Know | 9. No Response

4. Food / Accommodation

1. Yes | 2. No | 8. Do not Know | 9. No Response

5. Other expenses

1. Yes | 2. No | 8. Do not Know | 9. No Response

713CKPT.

1. If ALL 713==1 ↓	2. If ANY 713==2, 8, or 9 →GO TO 715

714. If Yes: How much did you pay for all these things?

- ENTER value in FCFA
- Last digit must be 5 or 0 (estimation)
- If not paid, ENTER 0
- If unknown, ENTER 8888
- If no response, ENTER 9999

0-9999

715. What treatment did your child receive?

- RECORD ALL MENTIONED

1. Antibiotic | 2. Antimalarial | 3. Paracetamol | 4. Ibuprofen | 5. Medicinal Plant

6. Another medicine-specify (715S. Specify (String 100)) | 88. Do not Know | 99. No Response

716A. [NAME], [FIRST DRUG 616] = FIRST RESPONSE TO 616
How long after the onset of diarrhea, did [NAME] start taking the [FIRST DRUG 616]?

0. Same day	1. Next day	2. Two days after diarrhea	3. Three days or more after diarrhea	8. Do not Know

716B. [NAME], [SECOND DRUG 616] = SECOND RESPONSE TO 616
How long after the onset of diarrhea, did [NAME] start taking the [SECOND DRUG 616]?

0. Same day	1. Next day	2. Two days after diarrhea	3. Three days or more after diarrhea	88. Do not Know	99. No Response

716C. [NAME], [THIRD DRUG 616] = THIRD RESPONSE TO 616
How long after the onset of diarrhea, did [NAME] start taking the [THIRD DRUG 616]?

0. Same day	1. Next day	2. Two days after diarrhea	3. Three days or more after diarrhea	88. Do not Know	99. No Response

Access to essential care for the respondent

800. Have you had an illness or injury in the last six months?

- Clarification: unrelated to pregnancy or childbirth

1. Yes	2. No →END OF INTERVIEW	8. Do not Know →END OF INTERVIEW	9. No Response →END OF INTERVIEW

↓

801. Did you seek advice or treatment for the diarrhea from any source?

1. Yes →GO TO 803	2. No	8. Do not Know	9. No Response
	↓	↓	↓

802. Why did you not get advice or treatment?

- PROBE: Other reasons?
- RECORD ALL MENTIONED

1. I could not pay the fee
2. I could not cross the distance to the health center
3. I do not make decisions about seeking health care
4. I arrived at the health center, but there was no equipment, material, or infrastructure to give me care
5. The prescribed medication was not available
6. I arrived at the health center, but I did not find staff qualified enough to give me care
7. I did not know what to do
8. I did not think it was serious enough
9. I did not have enough time
10. Other-specify (802S. Specify (String 100))

88. Do not Know

99. No Response

→END OF INTERVIEW

803. Where did you seek advice or treatment? Please list all sources in the order that you got them.

- PROBE: Anywhere else?
- PROBE TO IDENTIFY THE SOURCE
- MARK 1, 2, 3 FOR THE ORDER TO VISIT
- IF ONLY 1 (OR 2) LOCATIONS, MARK "WITHOUT RESPONSE" FOR 2ND (OR 3RD) LOCATION
- If primary health center, SPECIFY which one

1. At my house	2. National hospital	3. Regional hospital	4. Community referral hospital (Bankass)
5. Primary health center-specify (803S. Specify (String 100))	6. Private Clinic / Hospital	7. Pharmacy	8. At CHW
9. Home of CHW	10. At a traditional healer	11. At home, a travelling seller	12. Shop
13. Other-specify (803S. Specify (String 100))	88. Do not Know	99. No Response	

804. Did someone evaluate you before you went to the health center? If so, who was it?

0. No	→GO TO 808
1. Myself	→GO TO 808
2. Husband	→GO TO 808
3. Other women	→GO TO 808
4. Other close relatives	→GO TO 808
5. Friends / neighbors	→GO TO 808
6. CHW	→GO TO 805
7. Doctor	→GO TO 808
8. Nurse	→GO TO 808
9. Midwife	→GO TO 808
10. Matron	→GO TO 808
11. Traditional healer	→GO TO 808
12. Drug seller	→GO TO 808
88. Do not Know	→GO TO 808
99. No Response	→GO TO 808

805. Where was the evaluation conducted?

1. At home	↓
2. CHW Site	→GO TO 807
3. Other-specify (805S. Specify (String 100))	↓
8. Do not Know	↓
9. No Response	↓

806. How much time did it seem to take the CHW to arrive at your house?

1. Very long	2. Long	3. Normal	4. Short	5. Very short	88. Do not Know	99. No Response

807. How long did the evaluation last?

1. Less than an hour (less than the time between fitri and saafo)	2. 1 hour (time between fitri and saafo)	3. 2 hours (time between salifana and star)
4. ½ day	5. 1 day	6. 2 or more days
88. Do not Know	99. No Response	

808. How much time did it seem to take to get to the health center?

- If you went to several health centers, this is the last facility you attended.

1. Very long	2. Long	3. Normal	4. Short	5. Very short	8. Do not Know	9. No Response

809. At the health center, how long did you wait for care?

1. Less than an hour (less than the time between fitri and saafo)	2. 1 hour (time between fitri and saafo)	3. 2 hours (time between salifana and star)

4. ½ day	5. 1 day	6. 2 or more days	88. Do not Know	99. No Response

810. How did the waiting time seem to you?

1. Very long	2. Long	3. Normal	4. Short	5. Very short	88. Do not Know	99. No Response

811. How long did the medical consultation take?

1. Less than an hour (less than the time between fitri and saafo)	2. 1 hour (time between fitri and saafo)	3. 2 hours (time between salifana and star)

4. ½ day	5. 1 day	6. 2 or more days	88. Do not Know	99. No Response

812A. Did you take time off from work to go to the clinic? That is to say, the work you do that generates income.

1. Yes	2. No	8. Do not Know	9. No Response

812B. If so, how many days?

- If no leave taken, ENTER 0
- If unknown, ENTER 88
- If no response, ENTER 99

0-99

813. While seeking care for your child, which of the following expenses did you obtain?

1. Medication / treatment

1. Yes	2. No	8. Do not Know	9. No Response

2. Laboratory / diagnostic tests

1. Yes	2. No	8. Do not Know	9. No Response

3. Other health expenses

1. Yes	2. No	8. Do not Know	9. No Response

4. Food / Accommodation

1. Yes	2. No	8. Do not Know	9. No Response

5. Other expenses

1. Yes	2. No	8. Do not Know	9. No Response

813CKPT.

1. If ALL 813==1 ↓	2. If ANY 813==2, 8, or 9	→END OF INTERVIEW

814. If Yes: How much did you pay for all these things?

- ENTER value in FCFA
- Last digit must be 5 or 0 (estimation)
- If not paid, ENTER 0
- If unknown, ENTER 8888
- If no response, ENTER 9999

0-9999

END OF INTERVIEW

Before submitting the survey, return the census and ensure that the husband of the woman surveyed was identified if it is a usual resident of the household.
